# Supplementary material for: Structure and function of otoferlin, a synaptic protein of sensory hair cells essential for hearing
Source: Sci Adv. 2025 Oct 15;11(42):eady8532. doi: 10.1126/sciadv.ady8532 (PMC12526402; doi:10.1126/sciadv.ady8532)
Supplement: 20251015-1 [file sciadv.ady8532.v1.pdf]

NEUROSCIENCE

Structure and function of otoferlin, a synaptic protein of sensory hair cells essential for hearing

Han Chen[1,2,3,4]†, Constantin Cretu[1,2,4]†, Abigail Trebilcock[1,2,3,4], Natalia Evdokimova[1,2,3,4], Norbert Babai[1,2], Laura Feldmann[1,2,4], Florian Leidner[5], Fritz Benseler[6], Sophia Mutschall[1,2,4], Klara Esch[1,2,3], Csaba Zoltan Kibedi Szabo[7], Vladimir Pena[7], Constantin Pape[4,8,9], Helmut Grubmüller[4,5], Nicola Strenzke[1,10,11,12], Nils Brose[4,6,12], Carolin Wichmann[1,12,13,14], Julia Preobraschenski[1,4,12,15]*, Tobias Moser[1,2,4,9,12]*

Copyright © 2025 The Authors, some rights reserved; exclusive licensee American Association for the Advancement of Science. No claim to original U.S. Government Works. Distributed under a Creative Commons Attribution License 4.0 (CC BY).

Hearing relies upon speedy synaptic transmission of sound information from inner hair cells (IHCs) to spiral ganglion neurons. To accomplish this, IHCs use a sophisticated presynaptic machinery including the multi-C_2 domain protein otoferlin that is affected by human deafness mutations. Otoferlin is essential for IHC exocytosis, but how it binds Ca^{2+} and the target membrane to serve synaptic vesicle (SV) tethering, docking, and fusion remained unclear. Here, we obtained cryo–electron microscopy structures of otoferlin and employed molecular dynamics simulations of membrane binding. We show that membrane binding by otoferlin involves C_2B-C_2G domains and repositions C_2F and C_2G domains. Disruption of Ca^{2+}-binding sites of the C_2D domain in mice altered synaptic sound encoding and eliminated the Ca^{2+} cooperativity of IHC exocytosis, indicating that it requires the binding of several Ca^{2+}-ions by otoferlin. Together, our findings elucidate molecular mechanisms underlying otoferlin-mediated SV docking and support the role of otoferlin as Ca^{2+} sensor of SV fusion in IHCs.

INTRODUCTION

Inner hair cells (IHCs) and spiral ganglion neurons (SGNs) form highly specialized ribbon synapses to achieve indefatigable sound encoding at spike rates of hundreds per second and with submillisecond temporal precision (*1*–*3*). These high functional demands of reliably releasing SVs at even greater rates in synchronicity with the stimulus are thought to have shaped an unconventional molecular synaptic machinery that deviates from synapses of the central nervous system [CNS, (e.g. *4*–*13*)]. However, compared to the well-studied CNS synapses (*14*, *15*), the composition and structure of this machinery remain insufficiently understood (*1*). In particular, the identity of the Ca^{2+} sensor of SV fusion, the role of soluble *N*-ethylmaleimide–sensitive factor attachment protein receptors (SNARE) proteins and SNARE regulators, and, more generally, the SV fusion mechanisms at the IHC-SGN synapse are poorly characterized (*11*–*13*, *16*).

Human deafness mutations leading to auditory synaptopathy (*17*) affect at least two key players of the IHC synaptic machinery, otoferlin and $Ca_V1.3$ Ca^{2+} channels (*18*–*20*). $Ca_V1.3$ Ca^{2+} channels couple the IHC receptor potential to SV exocytosis (*21*–*24*). Otoferlin is a Ca^{2+}-sensitive multi-C_2 domain protein and belongs to the ancient family of ferlins involved in Ca^{2+}-dependent membrane fusion processes (*25*, *26*). Thought to be a core component of the SV-tethering and SV-fusion machinery (*6*, *12*, *27*), otoferlin is anchored through a single C-terminal transmembrane helix, cycles with SVs (*28*, *29*), and hence, it is found on both SVs and the plasma membrane of IHCs (*6*, *13*, *28*, *29*). Otoferlin comprises at least seven C_2 domains (C_2A-C_2G), of which four (C_2C, C_2D, C_2F, and C_2G) are thought to bind Ca^{2+}, involving aspartate residues in their top loops and likely also phospholipids in target membrane (*6*, *30*, *31*). In analogy to synaptotagmins 1 or 2 at CNS synapses (*32*, *33*), otoferlin might act as the Ca^{2+} sensor of SV fusion in IHC (*6*, *30*, *31*, *34*). Physiological data indicate that the cooperative binding of 4 to 5 Ca^{2+} ions to the Ca^{2+} sensor ("Ca^{2+} cooperativity") is required for SV fusion to occur in IHCs (*24*, *35*).

The analysis of mouse mutants for *Otof* and interacting proteins has indicated a multifaceted role of otoferlin in the SV cycle of IHCs (*17*). Beyond its putative role as Ca^{2+} sensor of SV fusion, otoferlin is required for SV replenishment to the readily releasable pool [RRP; (*12*, *27*, *31*, *36*, *37*)], likely acting as an SV-tethering and SV-docking factor (*12*), and for coupling exo- and endocytosis (*29*, *38*, *39*). However, even for the first postulated role as Ca^{2+}-sensor function of otoferlin in SV fusion, unequivocal demonstration has remained challenging. Possible reasons include (i) difficulties to disentangle otoferlin's action in Ca^{2+}-triggered SV fusion versus Ca^{2+}-dependent SV replenishment (*27*, *31*) and (ii) challenges in obtaining complete, high-resolution structures and mechanistic insights into the functions of all otoferlin domains, especially regarding their Ca^{2+}-sensing and membrane remodeling activities (*34*, *40*). To our knowledge, only a

[1]Institute for Auditory Neuroscience and InnerEarLab, University Medical Center Göttingen, Göttingen, Germany. [2]Auditory Neuroscience & Synaptic Nanophysiology Group, Max-Planck-Institute for Multidisciplinary Sciences, Göttingen, Germany. [3]Göttingen Graduate School for Neurosciences and Molecular Biosciences, University of Göttingen, Göttingen, Germany. [4]Cluster of Excellence "Multiscale Bioimaging: from Molecular Machines to Networks of Excitable Cells" (MBExC), University of Göttingen, Göttingen, Germany. [5]Department of Theoretical and Computational Biophysics, Max-Planck-Institute for Multidisciplinary Sciences, Göttingen, Germany. [6]Department of Molecular Neurobiology, Max Planck Institute for Multidisciplinary Sciences, Göttingen, Germany. [7]Research Group Mechanisms and Regulation of pre-mRNA Splicing, The Institute of Cancer Research, London, UK. [8]Institute of Computer Science, University of Göttingen, Göttingen, Germany. [9]CAIMed–Lower Saxony Center for Artificial Intelligence and Causal Methods in Medicine, Göttingen. [10]Auditory Systems Physiology Group, Institute for Auditory Neuroscience and InnerEarLab, University Medical Center Göttingen, Göttingen, Germany. [11]Department of Otolaryngology, University Medical Center Göttingen, Göttingen, Germany. [12]Collaborative Research Center 1690 (CRC1690), University of Göttingen, Göttingen, Germany. [13]Molecular Architecture of Synapses Group, Institute for Auditory Neuroscience and InnerEarLab, University Medical Center Göttingen, Göttingen, Germany. [14]Center for Biostructural Imaging of Neurodegeneration (BIN), University Medical Center Göttingen, Germany. [15]Biochemistry of Membrane Dynamics Group, Institute for Auditory Neuroscience and InnerEarLab, University Medical Center Göttingen, Göttingen, Germany.
*Corresponding author. Email: tmoser@gwdg.de (T.M.); julia.preobraschenski@med.uni-goettingen.de (J.P.)
†These authors contributed equally to this work.

crystal structure of the nonessential C_2A domain (*41*) and optical nanoscopy images of otoferlin (*42*) are available in this regard, while recently cryo–electron microscopy (cryo-EM) structures have been reported for myoferlin and dysferlin (*43*, *44*). Progress in understanding the structure and function of otoferlin is urgently needed, given that its defects cause deafness in humans with hundreds of missense mutations (*45*) that have remained hard if not impossible to interpret. Moreover, only a few years after preclinical proof of concept (*46*, *47*), recent clinical gene therapy trials to remedy otoferlin-related deafness have yielded first promising results (*48*–*50*), and detailed information on the otoferlin structure and function is needed to optimize these translational approaches.

Here, we pursued a multidisciplinary approach to elucidate the long-standing question of how otoferlin operates at IHC synapses. Using high-resolution cryo-EM structures and molecular dynamics (MD) simulations, we reveal how otoferlin engages and remodels lipid membranes for SV docking and SV fusion, highlighting prominent roles for the N-terminal C_2B and the Ca^{2+}-bound C-terminal C_2F and C_2G domains (*31*, *37*, *48*). The multiscale physiology of sound encoding in *Otof* C_2D mouse mutants revealed a reduced SV release probability and loss of physiological Ca^{2+} cooperativity, indicating that otoferlin indeed serves as Ca^{2+} sensor of SV fusion in IHCs.

RESULTS

Cryo-EM structures of mouse otoferlin in the lipid-free and membrane-bound states

We engineered a soluble variant of mouse otoferlin (residues 216 to 1931) for structural studies (Fig. 1A and fig. S1A), which were guided by the recent elucidation of myoferlin and dysferlin structures (*44*). The optimized construct lacks the nonessential N-terminal C_2A (*51*) and the C-terminal transmembrane helix (Fig. 1A) (*44*) while retaining part of the C_2A-C_2B linker (residues 216 to 265), which is important for sample stability. The purified otoferlin variant was well-folded (fig. S1, B and C), retained Ca^{2+}-binding activity (fig. S1D), and interacted with anionic lipid membranes (fig. S1, E and G), showing a stronger preference for Ca^{2+} over Mg^{2+} compared to myoferlin (fig. S1G) (*44*). Size exclusion chromatography (SEC) and mass photometry of soluble otoferlin (216 to 1931) predominantly revealed monomeric species (fig. S1, B and C and F).

To gain insights into how otoferlin engages and remodels lipid membranes, we determined cryo-EM structures of otoferlin (216 to 1931) in the lipid-free (Fig. 1, B and C) and membrane-bound states (Fig. 1, D and E; and figs. S2, A to E, S3, A to C, S4, A to C, and S5, A to D), resolved at global resolutions of ~2.9 to 3.5 Å and ~2.2 to 2.3 Å, respectively (tables S1 and S2). For the membrane-bound structures, we used membrane scaffold protein 2 N2 (MSP2N2)–based nanodiscs as lipid bilayer surrogates (figs. S2, A to C and S3, A to D) (*52*) and assembled the otoferlin-nanodisc complexes in the presence of Ca^{2+}. The presence of both lipids and Ca^{2+} significantly stabilized the otoferlin structure, enabling accurate modeling, in the near-atomic maps, of the C_2B-C_2G domains, several linker regions, multiple Ca^{2+} ions, and phospholipid–binding sites (Fig. 1, D to F; and fig. S2, C to H, S3, D to H, and S6 and S7). Conversely, in the lipid-free otoferlin structures (Fig. 1, B and C, and fig. S4, A to C and S5, A to D), C_2B, C_2F, and C_2G established loose interdomain interfaces, reflecting the increased dynamics of the C-terminal domains, particularly of C_2G (fig. S4, C to F, and fig. S5, E to I).

The nanodisc-bound otoferlin adopts a compact tertiary structure, reminiscent of a closed ring (Fig. 1, D and E; and figs. S3D and S7I; and movie S1), which spans ~128 Å by ~98 Å and closely resembles that formed by the lipid-bound myoferlin (*44*). The individual C_2 domains are arranged sequentially, from the N-terminal C_2B to the C-terminal C_2G domains, around the cavity of the ferlin ring. The well-resolved C_2C and C_2D domains form the top arch of the ring, which is rigidified by the C_2CD-FerA module (Fig. 1, D to F, and fig. S6, D to H), together referred to as the Fer^{core} module (*44*). C_2B and C_2E occupy diametrically opposed positions on the ring and form interfaces with C_2C and C_2D (figs. S6, A to C and S7I), respectively, as well as with the C-terminal C_2F and C_2G domains (Fig. 1, E and F, and fig. S7, C to I).

The tertiary structure of otoferlin in the membrane-bound state appears to be further reinforced by several accessory motifs and linker regions (Fig. 1, E and F). The C_2A-C_2B linker (residues 239 to 265), connecting the N-terminal C_2A (absent from the structure) to C_2B (fig. S6B), adopts an extended conformation, wrapping around the FerA four-helix bundle (residues 736 to 853) and C_2D before reaching the membrane-bound C_2B on the opposite side of the ring (Fig. 1, E and F, and fig. S7I). At the same time, the conserved FerI motif (residues 396 to 428), which links the C_2B and C_2C domains (fig. S6C), engages in a network of contacts with C_2B, C_2F, and the closely positioned anchor loop of C_2E (residues 1317 to 1343 and 1398 to 1421), partially resolved in the nanodisc-bound structures (Fig. 1, E and F, and fig. S7, A and B). These multiple interactions between N- and C-terminal otoferlin modules appear to serve as a platform for the recruitment and positioning of the C-terminal C_2G at the composite membrane interface (Fig. 1, E and F, and fig. S7, G to I). In contrast, in the lipid-free otoferlin structures (Fig. 1, B and C, and fig. S5, E to I), C_2G is poorly resolved and samples the conformational space between C_2B and C_2F, as also observed in lipid-free myoferlin and dysferlin (fig. S8, A and B) (*44*).

Membrane-binding interfaces of otoferlin

Irrespective of the used nanodisc composition, otoferlin engaged the lipid bilayer asymmetrically through a multidomain interface formed by the N-terminal C_2B and the C-terminal C_2F-C_2G (Fig. 2, A, C, and E; figs. S3D and S7I; and movie S2). The three domains are positioned close to each other on the nanodisc surface and their top loops (L1 and L3) project on the same side to deeply insert into the lipid membrane (Fig. 2C). As a result of these interactions, the ferlin ring appears tilted by ~40° with respect to the nanodisc plane (Fig. 2, C to E, and fig. S3D). Hence, the membrane arrangement of otoferlin differs from myoferlin structures whose C_2 domains are positioned in plane with the nanodisc membrane and establish more extensive contacts (fig. S8, C and D) through C_2C and the inner DysF domains (*44*).

However, same as in myoferlin structures (*44*), C_2B lacks the characteristic L1-L3 aspartate residues required for Ca^{2+} coordination. Consequently, C_2B engages the membrane via its concave side and binds through multiple residues positioned at the tip of the long L3 loop (Fig. 2B) in a Ca^{2+}-independent manner. The hydrophobic residues (L341 and L342) of C_2B appear to penetrate and thereby modulate the nanodisc bilayer structure, whereas the interacting basic residues (R343 and K339) establish superficial contacts at the surface, possibly with unresolved PS headgroups (Fig. 2B). In contrast, both C_2F and C_2G (Fig. 2, D and F) are oriented toward the nanodisc membrane with their top surfaces, allowing for the aspartate

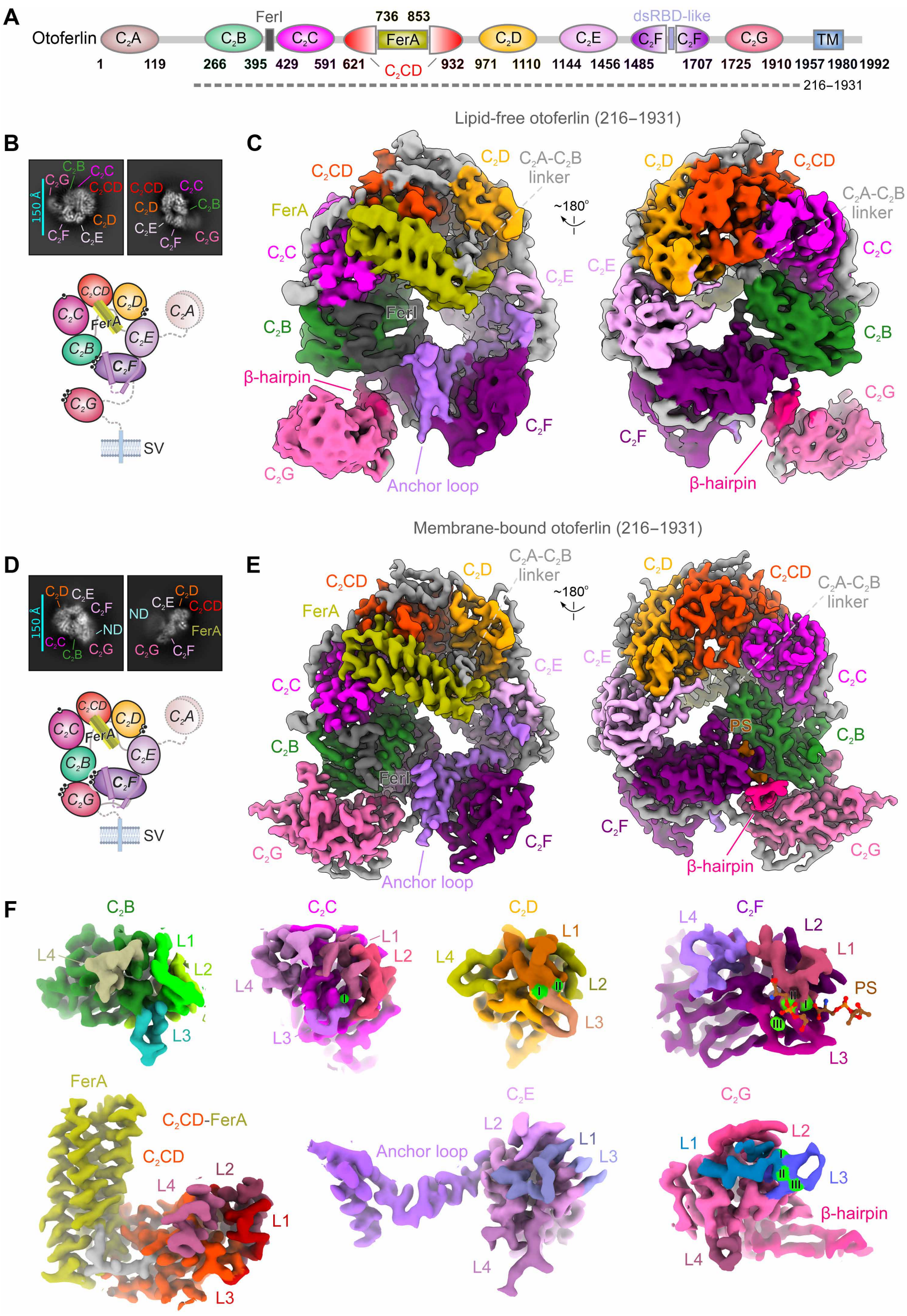

Fig. 1. Cryo-EM structures of mouse otoferlin in the lipid-free and membrane-bound states. (**A**) Schematic domain composition of mouse otoferlin based on the cryo-EM structures. The structural motifs of otoferlin are depicted as colored circles or boxes (domain colour scheme used throughout the manuscript), while the FerI motif and linker regions are colored in gray. The borders of the engineered otoferlin construct (residues 216 to 1931) used for structural studies are indicated with a dashed line. (**B**) Selected two-dimensional (2D) classes averages of otoferlin (216 to 1931) in the lipid-free, Ca^{2+}-bound state. The assigned otoferlin domains are labeled. The bottom panel shows a schematic representation of the structure, including a hypothetical position for the missing C_2A domain. (**C**) Overall cryo-EM maps of otoferlin (216 to 1931) in the lipid-free, Ca^{2+}-bound state. The cryo-EM map of otoferlin (class 2, low-pass–filtered to 3.9 Å) is colored based on the modeled domains, and the key structural elements are indicated. (**D**) 2D class averages and structure schematic of mouse otoferlin in the membrane-bound state. The MSP2N2 nanodisc (ND, cyan) density is indicated, along with the visible structural motifs. (**E**) Cryo-EM map of otoferlin (216 to 1931) in the membrane (nanodisc)-bound state, resolved at ~2.2-Å resolution. The unsharpened map (see also fig. S3C for the sharpened map) is shown in two orientations, with the structural motifs of otoferlin color-coded as in (C). (**F**) Cryo-EM maps of the individual C_2 domains of otoferlin, resolved in the lipid-bound state. The top loop-bound Ca^{2+} ions (green spheres, I to III) and two phosphatidylserines (PSs, brown) are viewed from the membrane-binding side.

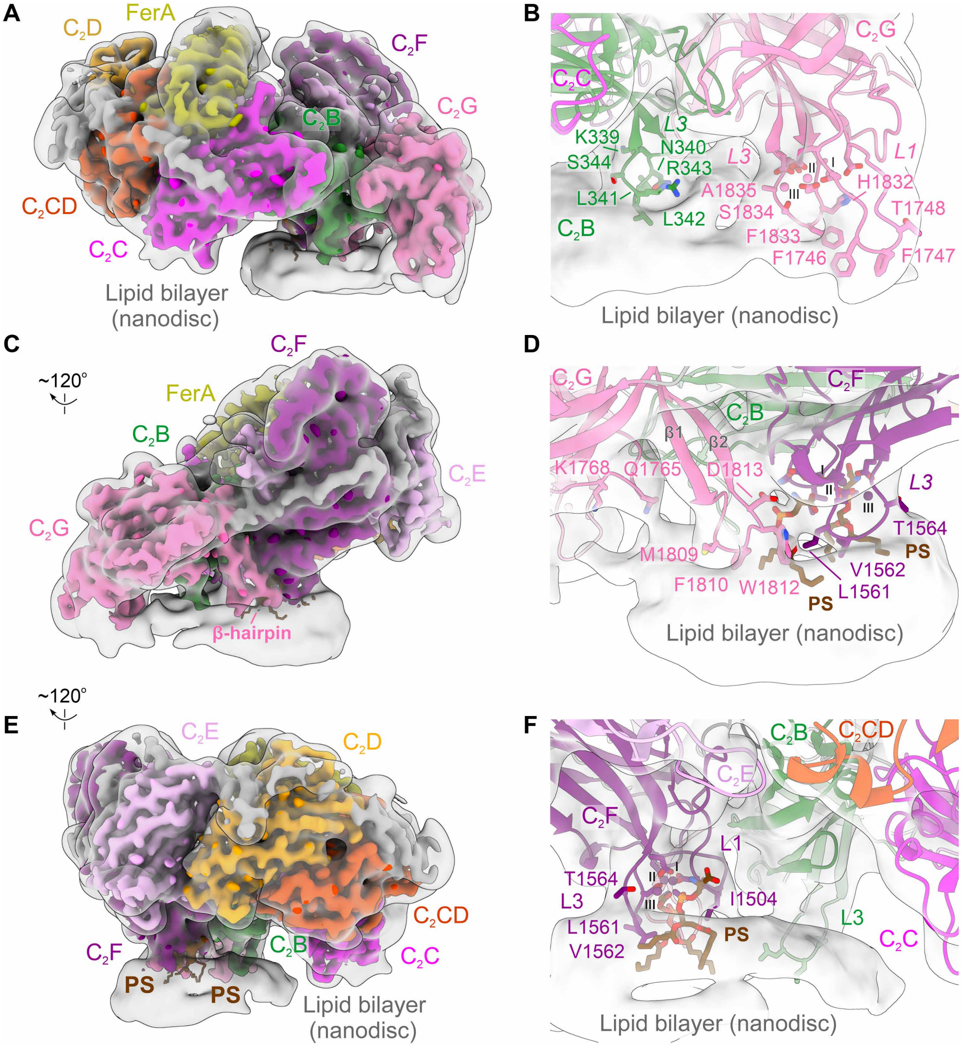

Fig. 2. The multidomain membrane-binding interface of otoferlin. (**A**) The high-resolution cryo-EM map of mouse otoferlin (216 to 1931) bound to a lipid nanodisc [comprising 30% PS and 10% PI(4,5)P_2] and viewed from its N-terminal side. The map has been colored as in Fig. 1E, and to enable the visualization of the ordered nanodisc regions, a transparent, low-pass–filtered (to 8 Å) map of the otoferlin-nanodisc complex has been superimposed onto the high-resolution map. (**B**) Lipid nanodisc contact interfaces of the C_2B and C_2G domains, oriented as in (A). The otoferlin model is depicted as cartoon. The nanodisc density is shown as a low-pass–filtered (to 8 Å) cryo-EM map. The interface and Ca^{2+}-coordinating residues are shown as sticks. The modeled Ca^{2+}-binding sites are indicated with roman numerals (I to III). (**C**) Composite lipid-binding interface formed by the C_2F and C_2G domains. The otoferlin maps are displayed as in (A). (**D**) Close-up view of the membrane interacting β-hairpin of C_2G and the L3 loop of C_2F. The otoferlin residues projecting toward the surface of the lipid nanodisc are shown as sticks, and the model is fitted inside the low-passed (to 8 Å) map of the otoferlin-nanodisc complex. The two recruited phosphatidylserine (PS) headgroups are depicted as sticks and colored brown. (**E**) C_2C, C_2D, and C_2E do not engage the lipid membrane in the otoferlin-nanodisc complex. The cryo-EM otoferlin-nanodisc maps are rendered and colored as in (A). (**F**) C_2F recruits two PS headgroups, originating from the nanodisc bilayer to its three Ca^{2+}-binding sites. The lipid interface residues are depicted as sticks and the PS molecules are colored brown. See also (D).

residues of L1 and L3 to coordinate Ca^{2+} and engage the lipid bilayer directly through the bound ions, consistent with these domains playing important roles in late-stage SV exocytosis (*34*, *53*). In both C_2F and C_2G, the bound Ca^{2+} ions form pockets for interaction with phospholipids and likely orient the L1 (C_2G) and L3 (C_2F and C_2G) loop residues to facilitate their membrane binding and insertion (Fig. 2, D and F), altogether resulting in a complex network of membrane contacts. Apart from the Ca^{2+}-facilitated membrane contacts, C_2F and C_2G are concomitantly engaged through a lipid-induced tertiary interface formed between the β-hairpin motif of C_2G and the L4 loop of C_2F. In addition, the β-hairpin of C_2G itself binds the lipid membrane through several hydrophobic residues (W1812, F1810, and M1809) while also framing the PS-binding pocket of C_2F (Fig. 2D).

Ca^{2+}- and lipid-binding sites of otoferlin

Biophysical studies of isolated C_2 domains of otoferlin estimated that two to three Ca^{2+} ions are bound per domain and that the domains have different Ca^{2+} sensitivity for binding the acidic phospholipids PS and PI(4,5)P_2 (*30*, *54*, *55*). In the high-resolution lipid-bound structures, we could model nine Ca^{2+} ions (C_2C: 1, C_2D: 2, C_2F: 3, and C_2G: 3) of which six, bound to C_2F and C_2G, are part of the

lipid interface of otoferlin (Fig. 3, A to C and E). Moreover, we could resolve two PS molecules of the nanodisc (Fig. 1F), contributing to the coordination of the three Ca^{2+} by the C_2F domain (Fig. 3D).

The structural data confirm the predicted Ca^{2+} coordination by top loop aspartates D1558 (D1563 in human) and D1560 (D1565 in human) of C_2F (Fig. 3C and fig. S7F). Together with D1565 (human D1570), these were replaced by alanine to disrupt Ca^{2+} binding in the *Otof*^{TDA} mouse mutant (triple aspartate to alanine, TDA), which abolished Ca^{2+} influx–triggered exocytosis in IHCs despite sizable (~60%) otoferlin levels (*34*). Here, we performed electron

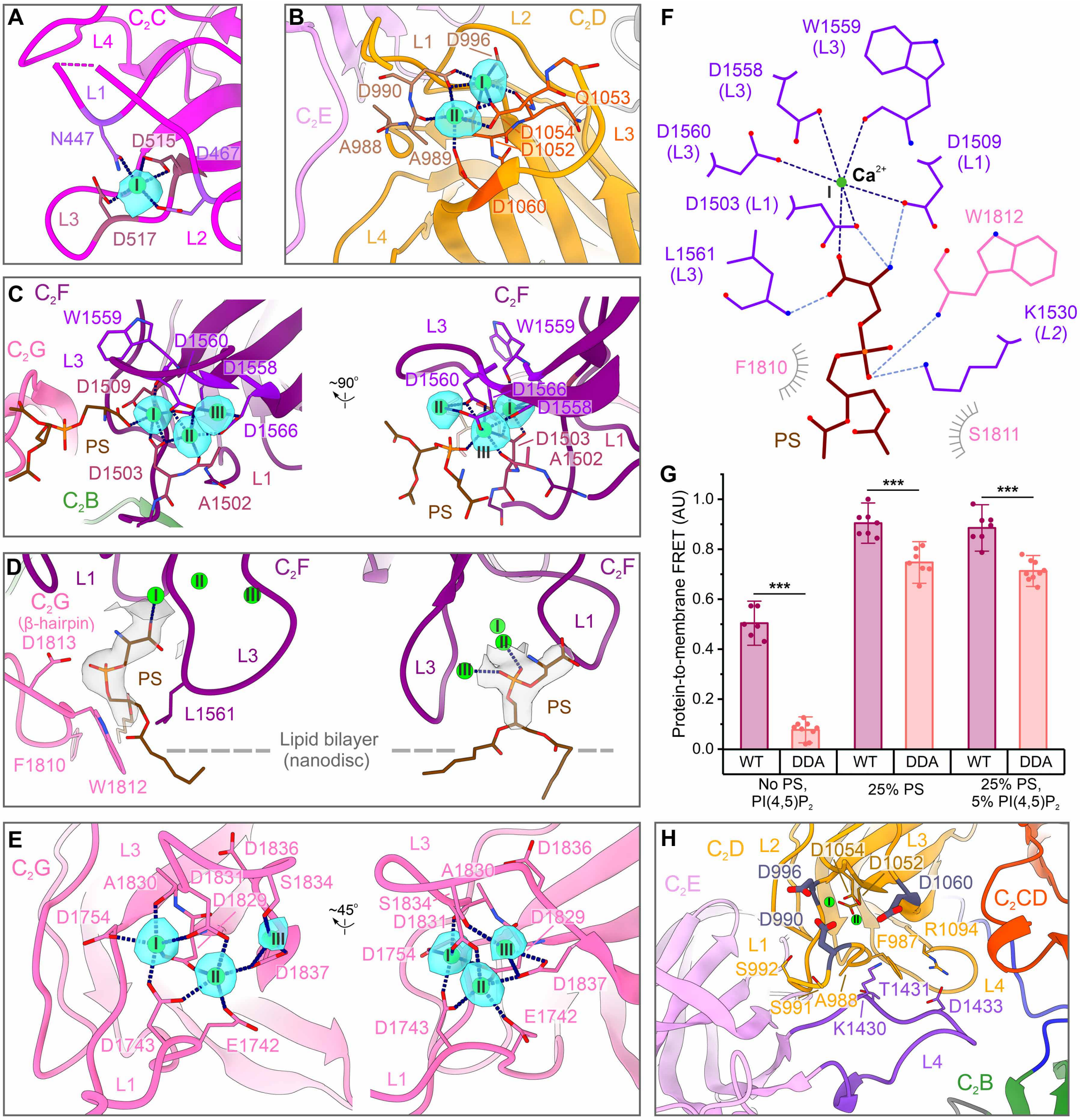

Fig. 3. Ca^{2+}- and lipid-binding sites modeled in otoferlin's membrane-bound structure. (**A**) Ca^{2+} binding by C_2C. Ca^{2+}-binding residues are depicted as sticks. The normalized cryo-EM difference map (cyan, 2σ) is contoured around the Ca^{2+} ion. (**B**) Two Ca^{2+} sites of C_2D. The cryo-EM difference map (cyan, 5σ) is contoured around the Ca^{2+} ions. (**C**) Ca^{2+} ions of C_2F. The three Ca^{2+} sites are shown together with the cryo-EM difference map (cyan, 5σ). (**D**) C_2F binds two PS molecules, originating from the lipid nanodisc. The cryo-EM density map (grey surface) is contoured around the modeled ligands. (**E**) C_2G coordinates three Ca^{2+} ions in the membrane-bound state. The model and the normalized cryo-EM difference map (cyan, 5σ) are displayed. (**F**) Schematic depicting the PS recognition by C_2F. The polar contacts to the glycerophosphate moiety are colored in light blue. The hydrophobic interactions are indicated as grey half-circles. (**G**) Liposome-binding activity of otoferlin (216 to 1931)–D1829A/D1831A [DDA, "double aspartate (D) to alanine (A)"] mutant of C_2G. The WT protein was analyzed in parallel. The measurements were repeated $n = 6$ (WT, in the presence of liposomes lacking PS and $PI(4,5)P_2$, "no PS, $PI(4,5)P_2$"), $n = 9$ (DDA, 25 mol% PS and 5 mol% $PI(4,5)P_2$ liposomes or "no PS, $PI(4,5)P_2$" liposomes), or $n = 7$ (remaining measurements). The data were normalized to the maximum Dansyl-PE emission (fig. S1G), and error bars indicate the SD (±1.5 SD). Two-sample comparisons were performed using an unpaired two-tailed *t* test. Significant differences between otoferlin WT and DDA samples are indicated as $***P < 0.001$. (**H**) Tertiary interface between C_2D and the extended L4 loop of C_2E, which might be sensitive to Ca^{2+}. C_2D residues substituted in the mouse models (see also Fig. 6) are indicated in dark blue. The two Ca^{2+} sites of C_2D are shown as spheres and colored green. AU, arbitrary unit.

tomography of IHC synapses in conventionally embedded organs of Corti of *Otof*^{TDA} mice and did not find an obvious SV-docking deficit (fig. S9). This might suggest that TDA-otoferlin retains sufficient membrane binding activity but is yet unable to promote efficient SV fusion. However, we cannot exclude a deficit in tight SV docking or Ca^{2+}-dependent priming that could be essential for SVs to gain full fusion competence (*56*). C_2F Ca^{2+} coordination further involves D1509, A1502 (backbone carbonyl) and D1503 residues of L1 and D1558 and W1559 (backbone carbonyl) residues of L3 (Fig. 3C). Same as in myoferlin structures (*44*), the two PS headgroups bound by C_2F complete the coordination shells of the first and third Ca^{2+} ions. The side chains of the two bound PS are only partially embedded into the lipid nanodisc, suggesting that they were "pulled" from the nanodisc bilayer by C_2F (Fig. 3, D and F, and fig. S7E). The PS at the first Ca^{2+}-binding site appears to establish additional hydrophobic interactions with residues F1810 and W1812 of C_2G's β-hairpin that line the binding pocket and, likely, stabilize the bound PS. This dual PS (Fig. 3F) recognition mechanism used by C_2F appears unique and likely evolved to facilitate a high degree of membrane remodelling (Fig. 3, D and F, and figs. S7E and S8E).

Same as C_2F, C_2G coordinates three Ca^{2+} ions through residues of the L1 (E1742, D1743, and D1754) and L3 [D1829 and A1830 (backbone carbonyl), D1831, S1834, and D1837] loops (Fig. 3E and fig. S7H). D1837 (along with D1836) was substituted by alanine in *Otof*^{DDA} mice (*53*) that showed reduced (~60%) basolateral otoferlin levels in IHC, abolished IHC exocytosis, and deafness. The top loops of C_2G are rich in aromatic residues [F1746 (L1), F1747 (L1), F1833 (L3), and Y1775 (L2)] and histidines [H1776 (L2) and H1832 (L2)], which all appear to either insert into the membrane or position close to it. As no Ca^{2+}-recruited phospholipids could be modeled (fig. S7, G and H), this suggests that membrane binding by C_2G involves a Ca^{2+}-independent component.

To discern between possible membrane-binding mechanisms, we introduced neutralizing substitutions in two key Ca^{2+}-binding aspartates of the C_2G domain, D1829 and D1831, and tested the liposome-binding activity of the D1829A/D1831A otoferlin mutant using a fluorescence-based assay (Fig. 3G). Although the otoferlin mutant retained significant lipid-binding activity through the C_2B and C_2F domains, the assay (Fig. 3G and fig. S1G) revealed a Ca^{2+}-sensitive reduction in liposome binding compared with the wild-type (WT) protein, particularly when the tested membranes lacked anionic phospholipids (0.08 ± 0.03 versus 0.50 ± 0.06, $P = 1.64 \times 10^{-10}$, $P < 0.001$). Therefore, Ca^{2+} binding by C_2G may still be needed for efficient membrane interaction, consistent with the strong exocytosis deficit in *Otof*^{DDA} IHCs (*53*). Potentially, Ca^{2+} binding to C_2G could rigidify and, consequently, help orient the long top loops of the domain toward the membrane plane, without conferring strict PS specificity.

Different from C_2F and C_2G, the Ca^{2+}-binding top loops of C_2C and C_2D in the membrane-bound otoferlin structure point away from the nanodisc membrane (Figs. 2E and 3, A and B, but see results of MD simulation below). In our structure, we observe that C_2C binds one Ca^{2+} by D467 and N447 residues of L1 and D515 and D517 residues of L3, substituted by alanine in *Otof*$^{D515-517A}$ mice (*31*), for which changes in the Ca^{2+} dependencies of SV fusion and replenishment were reported. Last, C_2D domain coordinates two Ca^{2+} ions mainly via the top loop aspartates: D990, D996 (L1), D1052 and Q1053 (backbone carbonyl) (L3) form the first Ca^{2+}-binding site, A989 (backbone carbonyl), D990 (LL1), and D1054 and D1060 (L3) coordinate the second Ca^{2+} ion (Fig. 3, B and H, and fig. S6, G and H). As in myoferlin structures (fig. S8, A to D), L1 of C_2D is closely positioned to the extended L4 of the adjacent C_2E (Fig. 3H and fig. S6H). This represents the only tertiary interface of otoferlin which might be sensitive to Ca^{2+} binding, raising the possibility that C_2D Ca^{2+} binding could serve a structural role and modulate the conformational states of the ferlin ring during the SV cycle. Therefore, we have studied the consequences of progressively disrupting the C_2D Ca^{2+}-binding sites on IHC exocytosis and synaptic sound encoding in mouse mutants (see below).

Conformational changes of otoferlin upon membrane binding

Whereas the top arch of the ferlin ring (C_2B-C_2D) is similar in the resolved otoferlin structures, the C-terminal C_2F and C_2G are rearranged in the membrane-bound state, generating a more compact "closed" conformation (see also movie S3). Comparison between the lipid-free "open" state and the nanodisc-bound closed state (see also movie S4), indicates C_2F moved locally by ~9 Å in the direction of C_2B and C_2E. At the same time, the C_2G domain, which samples multiple conformations in the lipid-free state (fig. S5, E to I), underwent a large-scale displacement by ~21 to 34 Å toward the nanodisc plane (fig. S5, C and E). In its new pose, C_2G established new contact interfaces, absent in the lipid-free state, with the convex surface of C_2B (Fig. 4B), the FerI motif (Fig. 4C), and C_2F via the β-hairpin (Fig. 4D), tailing a total ~901 Å^2. The combined domain movements appear to facilitate the in-plane positioning of the membrane-facing top loops of C_2B (L3), C_2F (L3), and C_2G (L1 and L3) and allow their cooperative interaction with the nanodisc through a multidomain binding interface. The formation of the new tertiary contacts, involving C_2G, appears to also rigidify the overall structure and fix the, otherwise dynamic, C_2B-C_2F and C_2E-C_2F interfaces (fig. S5, E to G), further promoting stable membrane binding.

Compared to myoferlin structures (*44*), the observed conformational transition of otoferlin is less complex in nature, and except C_2G, all interacting domains are posed to engage the membrane, without requiring significant rearrangement from the lipid-free state (fig. S8, A to D). It is, therefore, possible that the prepositioning of C_2B, likely through the specific C_2A-C_2B linker region (residues 239 to 265) and, to a large extent, of C_2F has evolved to support the fast timescales of otoferlin-dependent SV exocytosis in hair cells (milliseconds) (*57*–*59*). In contrast, dysferlin/myoferlin-dependent fusion events occur in tens of seconds (*60*) and might not face the need for fast and reversible tethering and docking of vesicles. Likely, for a similar reason, the membrane-proximal C_2G of otoferlin is highly dynamic in the lipid-free state, a feature that might increase the target membrane capture radius of otoferlin to promote both fast SV docking and SV undocking, which we postulate to support the high rates of otoferlin-dependent SV replenishment. Further supporting this hypothesis, the computational analysis of otoferlin's conformational space in the Ca^{2+}-bound lipid-free state indicates that "closed-like" conformations are sampled in the absence of a membrane, together with additional, likely, less stable, intermediate states (figs. S4, C to E and S5, E and F). In these states, the C_2G domain occupies both peripheral positions (farther away from C_2B) and appearing to transiently engage C_2F and closely approach the N-terminal C_2B (figs. S4D and S5, E and F). In contrast, only a minority of otoferlin particles (~2.5%) from the nanodisc-bound dataset display alternative conformations of C_2G (fig. S2C).

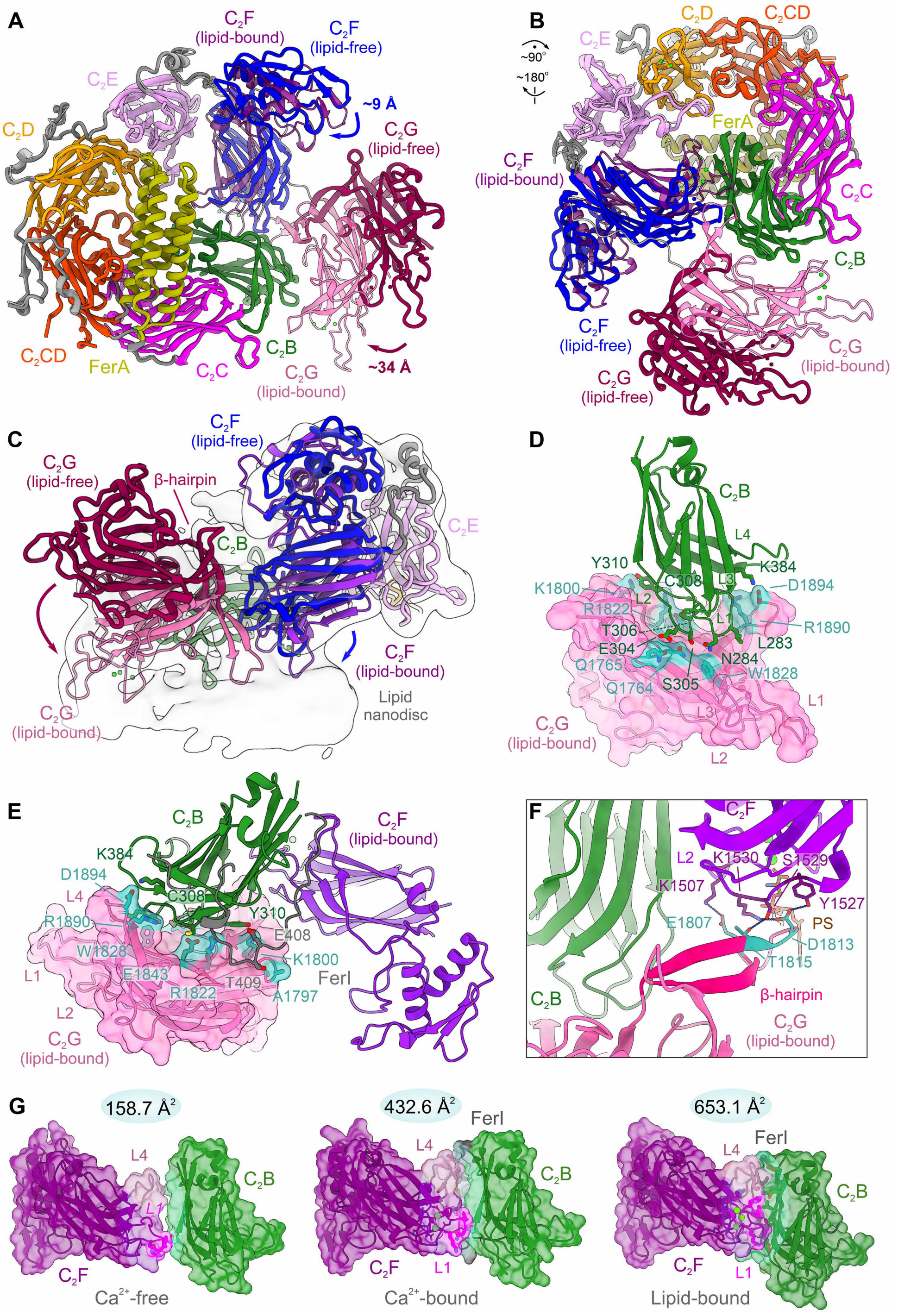

Fig. 4. Conformational states of otoferlin. (**A**) Structural superposition of lipid-free (open) and nanodisc-bound (closed) states of otoferlin (216 to 1931). The closed-state model is depicted as a transparent cartoon. (**B**) C_2F (blue) and C_2G (dark red) domains undergo a large-scale repositioning upon binding to a nanodisc bilayer. The superimposed lipid-free and lipid-bound models of otoferlin are shown from the membrane-binding side. (**C**) Repositioned C_2F and C_2G domains in the closed state are engaged in extended contacts with the lipid nanodisc. The otoferlin models in the closed and open states are shown together with a low-pass–filtered map (to 8 Å) of the otoferlin-nanodisc complex. The C_2F and C_2G domains in the lipid-free state are colored blue and dark red, respectively. Their movement upon nanodisc binding is indicated by arrows. (**D**) New tertiary interface between the C_2B and C_2G domains established in the closed (membrane-bound) state of otoferlin. The C_2B and C_2G residues engaged in both polar and hydrophobic interactions (within 3.5 Å) are depicted as sticks. C_2G is shown as a transparent solvent excluded surface. (**E**) Contact interfaces between C_2B, C_2G, and the FerI motif in the closed state of otoferlin: Interacting residues within a 3.5-Å distance are depicted as sticks. The FerI motif is colored gray, and C_2G is shown as a transparent solvent excluded surface. (**F**) Polar contacts between the β-hairpin motif of otoferlin and the top loop L2 of C_2F observed in the lipid-bound state of otoferlin. Polar contacts are depicted as dashed blue lines, whereas the neighboring C_2B is colored green. (**G**) C_2F-C_2B interface areas in the three resolved otoferlin states. C_2B and C_2F are shown as transparent, solvent-excluded surfaces. The estimated interface areas indicated above. Domain models are depicted as cartoons, and key interface residues are shown as sticks.

To assess the effect of Ca^{2+} binding on the conformational transitions of otoferlin, we obtained an additional ~3.5-Å cryo-EM structure of otoferlin (216 to 1931) in the absence of Ca^{2+} ions and lipids (fig. S5, A to D). The Ca^{2+}-free structure closely resembled the Ca^{2+}-bound lipid-free otoferlin (Fig. 1C and fig. S5, G to I) but showed a reduced C_2B-C_2F interface area (~159 Å^2 versus ~433 Å^2 and ~653 Å^2 in the Ca^{2+}-bound and lipid-bound states, respectively, Fig. 4G and fig. S5H). Moreover, otoferlin did not sample closed-like conformations in the absence of Ca^{2+} (figs. S4E and S5, B to D). The FerI motif, folded between C_2B and C_2F-C_2G in the Ca^{2+}-bound state, was destabilized and no longer engaged the L4 and the base of the L1 loop of C_2F, possibly reflecting an increased dynamics of C_2F's top loops in the absence of Ca^{2+} and lipids (root mean square deviation ~ 3.08 Å compared to the Ca^{2+}-bound structure; fig. S5, G to I). In contrast, the tertiary organization of the Fer^{core} module (C_2C-C_2D) and C_2B were not affected by Ca^{2+} or PS binding (fig. S5, F to I). From these analyses, we conclude that Ca^{2+} binding might be needed to promote tighter interactions between the N-terminal C_2B and the C-terminal C_2F-C_2G domains, leading to a closed otoferlin state, which we could evidence in the absence of lipid membranes (fig. S4, C and E). Consequently, the selection of the closed state of otoferlin—where C_2B, C_2F, and C_2G are engaged in stable tertiary interfaces—is likely achieved through a membrane binding mechanism from an ensemble of sampled conformations in the lipid-free state.

Molecular dynamics simulations of the interaction of otoferlin with the target membrane

We cannot exclude that the other C_2 domains interact with the target membrane en route to SV docking or SV fusion. The role of the C_2C domain in preparing SVs for fusion (*31*, *37*) indicates that the domain might be required in interaction with the membrane and/or active zone (AZ) proteins for a membrane-proximal SV to become fusion competent. There is biochemical evidence of lipid binding by several C_2 domains [e.g., (*25*, *50*)]. Moreover, it is possible that we missed lipid interactions of other C_2 domains, e.g., in case they caused extensive remodeling and hence destabilization of the nanodisc membrane. To gain more detailed insight into the nature and driving forces of the interactions between otoferlin and the membrane, we used atomistic MD simulations.

We used the membrane- and Ca^{2+}-bound cryo-EM structure as a starting point and placed it so that its center of mass was ~4.5 nm above the membrane. The membrane was modeled with CharmmGUI (*61*) and contained anionic lipids PS (30%) and PI(4,5)P_2 (10%) mimicking the composition of the nanodisc. Otoferlin formed extensive contacts with the lipid membrane during the first microsecond of simulation (fig. S10). Internal distances between otoferlin residues differed by less than 5 Å from the original cryo-EM conformation, excluding disordered loops and the mobile C_2G domain (fig. S10). The simulations reproduced the interactions between anionic lipids and the C_2B, C_2F, and C_2G domains observed in the cryo-EM structure (Fig. 2). However, different from the cryo-EM structure, otoferlin formed a more extensive lipid interface in MD simulations involving all modeled C_2 domains. Although all C_2 domains were in contact with the membrane, most contacts were made by C_2C and the C-terminal domains (Fig. 5B). In contrast to the top loops of other C_2 domains, C_2C formed an amphiphilic helix, which got submerged into the membrane (Fig. 5A). C_2E formed a prominent membrane interface and strongly engaged with PI(4,5)P_2 (Fig. 5E), which was mediated by the multiple basic residues in the top loops rather than by Ca^{2+} (Fig. 5D). C_2D also engaged the membrane with its top loops, although the number of lipid contacts formed was lower than for the other Ca^{2+}-binding C_2 domains (Fig. 5B). Although C_2D bound two Ca^{2+} ions, it did not bind PI(4,5)P_2 or PS more often than phosphatidyl choline (PC; Fig. 5E). The Ca^{2+}-binding top loops of C_2D were pulled very close toward the membrane, rendering binding of large phosphorylated inositol headgroups of PI(4,5)P_2 sterically unfavorable. In contrast, PI(4,5)P_2 clustered around the Ca^{2+}-binding pockets of C_2C, C_2F, and C_2G domains (fig. S10). Moreover, different from C_2E, the C_2D top-loop periphery contains fewer basic residues, which could form an interface with anionic lipids (Fig. 5, C and D). Instead, the hydrophobic C_2D top loop, which inserts into the membrane, seems key for the interaction of the domain with the lipid membrane (Fig. 5C).

To test how important Ca^{2+} ions and PI(4,5)P_2 are for observed binding, we carried out two simulations of the membrane-bound otoferlin where we removed either Ca^{2+} or the PI(4,5)P_2 charges. After Ca^{2+} removal, the contacts between protein and membrane underwent a quick and long-term decrease, primarily because of conformational change in C_2G and its detachment (fig. S10). In contrast, removing charges from the PI(4,5)P_2 lipids did not have an immediate effect on membrane binding, and the number of lipid contacts was comparable to the unperturbed simulations (fig. S10). These findings are in line with biochemical data, suggesting that Ca^{2+} plays a more important role than anionic lipids in liposome binding by otoferlin (fig. S1G). Because neither of the perturbations caused rapid detachment of otoferlin from the membrane, we conclude that, once established, the otoferlin-lipid interface is reinforced and stabilized by hydrophobic interactions.

In vivo analysis of otoferlin function

In the light of our cryo-EM structures and simulations, revealing the multiple Ca^{2+}-binding domains, we next turned to in vivo analysis of otoferlin function to test the "Ca^{2+} sensor of SV fusion" hypothesis. Previous efforts had targeted Ca^{2+} binding to C_2C (*31*), C_2F (*34*), and C_2G (*53*) domains by replacing Ca^{2+}-coordinating aspartates of their top loops. Yet, these studies had supported but did not unequivocally demonstrate the Ca^{2+}-sensor role of otoferlin. For example, despite substantial otoferlin abundance in IHCs (≥60% of WT levels) and intact synapse number and structure (fig. S4), the C_2F (*34*) and C_2G (*53*) mutants lacked Ca^{2+} influx–triggered exocytosis, precluding in depth analysis of the Ca^{2+} dependence of SV fusion. Consistent with this demonstration of the critical role of C_2F and C_2G, our present structural characterization of otoferlin implicated these domains in membrane binding, likely required for SV docking and SV fusion (Fig. 2). C_2D, on the other hand, shares a reduced phospholipid interface (Figs. 2 and 5A). Hence, we reasoned that targeting its Ca^{2+}-binding sites would primarily alter Ca^{2+} binding by otoferlin, without exerting additional effects on SV docking or membrane remodeling for SV fusion.

Our structure-guided mutagenesis aimed to progressively disrupt Ca^{2+} binding to the C_2D domain: Substitution of aspartates D1060, D990, and D990/996 by alanine is predicted to alter the binding of one ($Otof^{D1060A}$ and $Otof^{D990A}$) or both ($Otof^{D990\text{-}996A}$) Ca^{2+} ions (Fig. 3). As *Otof* mutations often lead to decreased otoferlin protein levels, alter the subcellular otoferlin distribution, or cause a reduction in the number of afferent IHC-SGN synapses (*27*, *37*, *53*), we first performed immunohistochemistry in the organ of Corti

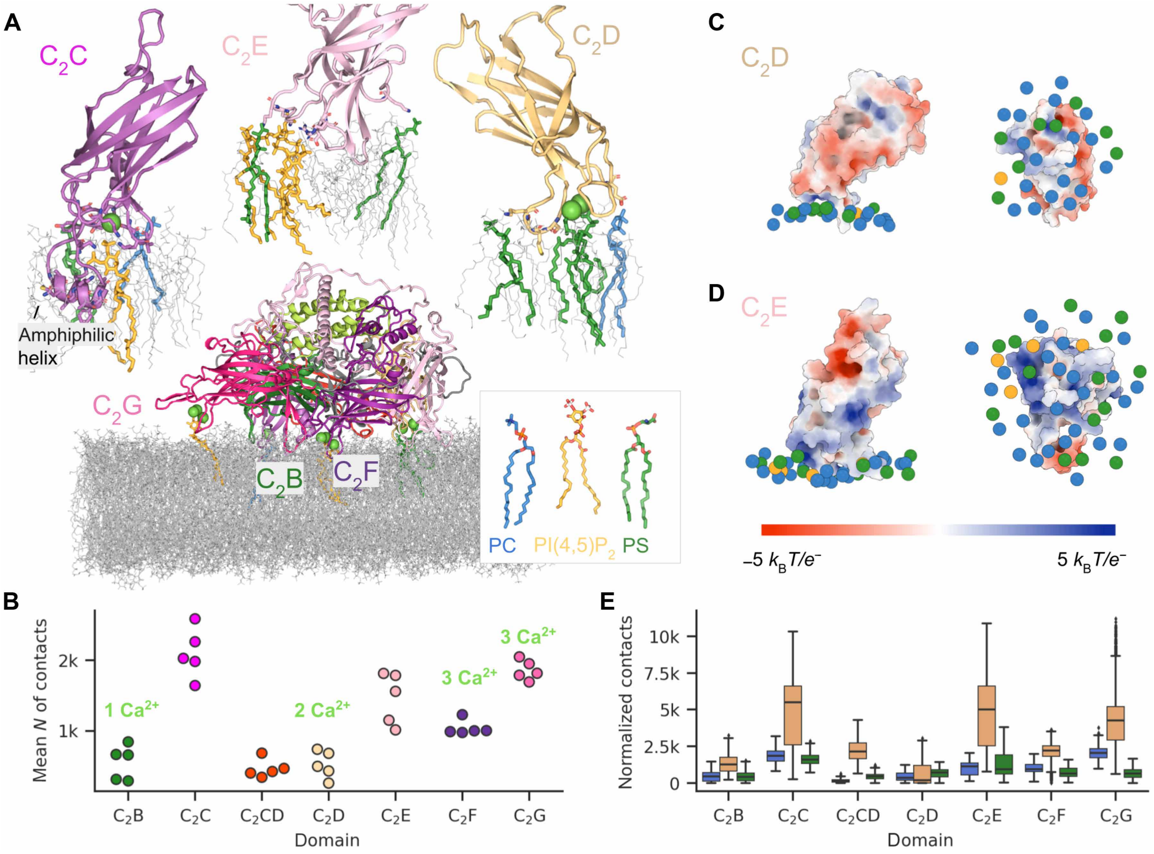

Fig. 5. Molecular dynamics simulations reveal protein-lipid interaction driving membrane docking. (**A**) Overview of the multi-domain lipid interface observed in otoferlin simulations. Lipids within 3 Å of Ca^{2+} ions are highlighted in the overview image. C_2C, C_2D, and C_2E domains are shown enlarged in inserts. (**B**) Numbers of protein-lipid contacts established by individual C_2 domains over the last 200 ns of simulations when the number of protein-lipid contacts plateaued (fig. S10B). Mean values are shown for five simulation replicates indicated by individual dots. The number of calcium ions bound to domains is indicated for reference. (**C** and **D**) Electrostatic potential mapped onto accessible surface areas of C_2D and C_2E domains, respectively. The potential was calculated with the APBS plugin in PyMol. Lipids within 15 nm of each domain are represented by their central phosphorus atom and colored according to the legend in (A) (also see fig. S10C for lipid densities at the C_2 domains). Side views are shown on the left, and bottom views are shown on the right. (**E**) Number of contacts between individual C_2 domains and every lipid type in the last 200 ns of five simulation replicates normalized by the lipid abundance in the model membrane, colored according to the legend in (A). The box boundaries represent the first and third quartiles of data distribution pooled together from five replicates, and the middle bar indicates the median value; the whiskers cover observations that are within 1.5 interquartile ranges of lower and upper quartiles; the rest of the data points are displayed individually.

early after hearing onset (2 weeks old) and after full cochlear maturation (5 to 8 weeks old). Immunofluorescence of the SV-marker Vglut3 (*7*, *62*) was unaltered in IHC of the mouse mutants and served as a reference for the subcellular distribution of otoferlin (Fig. 6, A to E, and figs. S11 and S12). We found the levels and subcellular distribution of otoferlin to be unaltered in IHCs of homozygous $Otof^{D990\text{-}996A}$ and $Otof^{D1060A}$ mice, while homozygous $Otof^{D990A}$ IHCs exhibited a mild reduction to 71.10 ± 0.03% of WT levels but unaltered subcellular otoferlin distribution (Fig. 6, D and E). The number of IHC-SGN synapses, quantified by counting juxtaposed spots of presynaptic RIBEYE/CtBP2 and postsynaptic Homer1 immunofluorescence, was normal in all three homozygous mutants (fig. S12).

$Otof^{D1060A}$, $Otof^{D990A}$, and $Otof^{D990\text{-}996A}$ mice showed significantly impaired synaptic transmission at IHC-SGN ribbon synapses of increasing severity as demonstrated by in vivo and ex vivo physiology. Cochlear amplification mediated by outer hair cells was intact as evident by unaltered distortion product otoacoustic emissions (DPOAE, Fig. 6F and fig. S13), supporting the notion of an auditory synaptopathy as the mechanism of hearing impairment (*17*). Recordings of auditory brainstem responses (ABRs, Fig. 6G) showed a mild[(~20-dB sound pressure level (SPL)] but significant increase of sound threshold for 2-month-old homozygous $Otof^{D990A}$ and $Otof^{D990\text{-}996A}$ mice (Fig. 6H) as compared to pooled littermate WT controls ($Otof^{wt}$). Homozygous 2-month-old $Otof^{D1060A}$ mice showed a nonsignificant trend toward higher ABR thresholds (Fig. 6H). The synchronous activation of the SGN population, approximated as amplitude of ABR wave I, was significantly impaired in all mutants. Wave I amplitudes declined with the anticipated strength of disrupting C_2D Ca^{2+} binding (Fig. 6I). Central ABR waves (waves II to IV) were less affected (Fig. 6, G and J), which likely reflects improved neural synchrony due to convergent input of SGNs into the cochlear nucleus neurons as previously reported for mouse mutants with impaired synaptic sound encoding (*29*, *36*, *37*, *63*).

Next, we turned to recordings from individual SGNs to scrutinize the impact of impaired Ca^{2+} binding on spontaneous and

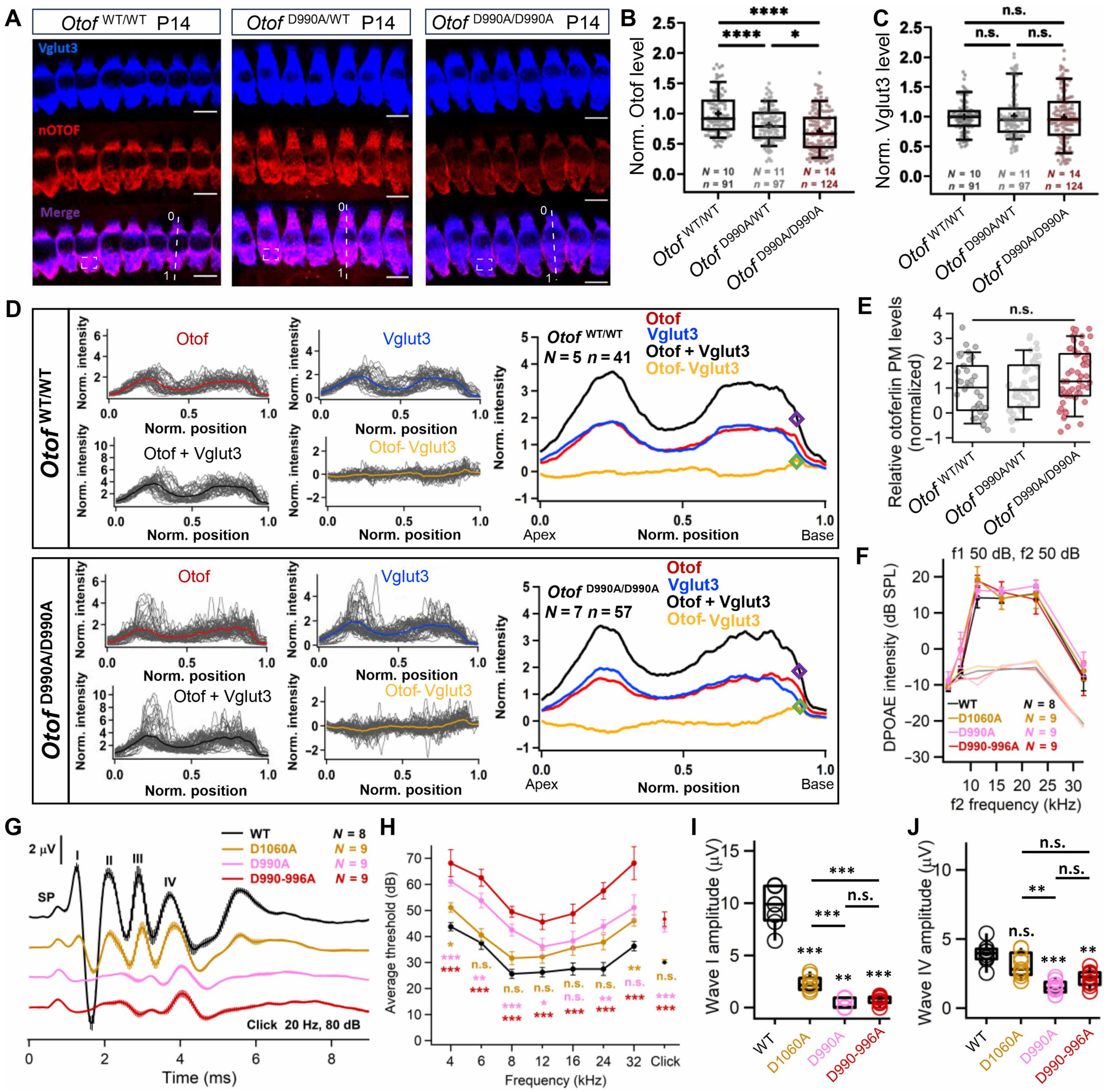

Fig. 6. Disruption of Ca^{2+}-binding by the C_2D domain of otoferlin impairs hearing. (**A**) Staining IHCs with otoferlin (OTOF) and Vglut3 antibodies. Otoferlin immunofluorescence intensity was averaged over basal regions of interest (white dashed boxes). White dashed lines indicate the line scans in (D), with 0 and 1 indicating start and end points. Scale bars, 10 μm. (**B**) Otoferlin immunofluorescence intensity indicated reduced level in IHCs of both heterozygote and homozygote *Otof*^{D990A} mice compared to IHCs of littermate controls. (**C**) Vglut3 immunofluorescence intensity indicated unchanged Vglut3 levels in *Otof*^{D990A} IHCs compared to littermate control IHCs. (**D**) Line profile analysis: For quantification of membrane staining, the fluorescence was normalized to the cellular fluorescence for each fluorophore. The sum of both fluorescence values (black line) was used to determine the position of the basal membrane. At the most basal cellular point along this line which exceeds the threshold value of 2 (purple diamond), the otoferlin-Vglut3 fluorescence difference (yellow line) gave the value for relative otoferlin plasma membrane levels (green diamond). (**E**) Relative levels of membrane-bound otoferlin at the basal pole of IHCs. (**F**) Robust DPOAE in homozygous *Otof*^{D1060A} (yellow), *Otof*^{D990A} (magenta), and *Otof*$^{D990-996A}$ (red) mice indicate normal OHC function across the cochlear frequency range: 2f1-f2 DPOAE amplitude for different f1 and f2 pairs of primary tones. (**G**) Auditory brainstem responses (ABRs) of *Otof*^{wt} (black), homozygous *Otof*^{D1060A} (yellow), *Otof*^{D990A} (magenta), and *Otof*$^{D990-996A}$ (red) mice to 80-dB click stimulation. (**H**) ABR thresholds, (**I**) ABR wave I amplitude, and (**J**) ABR wave IV amplitude [same color code as in (A)]. Box and whisker plots represent median, 25th and 75th percentiles, and 10th and 90th percentiles. Two sample comparisons were performed using *t* test or the Wilcoxon signed-rank test. Significant differences are indicated as $*P < 0.05$, $**P < 0.01$, and $***P < 0.001$. n.s., not significant.

sound evoked synaptic transmission from IHCs in vivo. Spontaneous SGN firing, reporting transmission at the IHC resting potential, tended to be lower in *Otof*^{D990A} and *Otof*$^{D990-996A}$ but not in *Otof*^{D1060A} mutants (fig. S14A). As expected, given intact cochlear amplification, we found thresholds and frequency tuning to be unaltered in SGNs of all three mutants (fig. S14, B and C). We then examined the SGN responses to suprathreshold tonebursts (30 dB above threshold, repetition rate of 5 Hz), which allows one to study release probability and RRP dynamics of IHC AZs (Fig. 7A) (*64*). Most SGNs in the mouse cochlea receive input from a single AZ (*65*), and their SGN firing rate reports the rate of SV release convolved with the well-understood neural refractoriness (*66–68*) that is unlikely to be

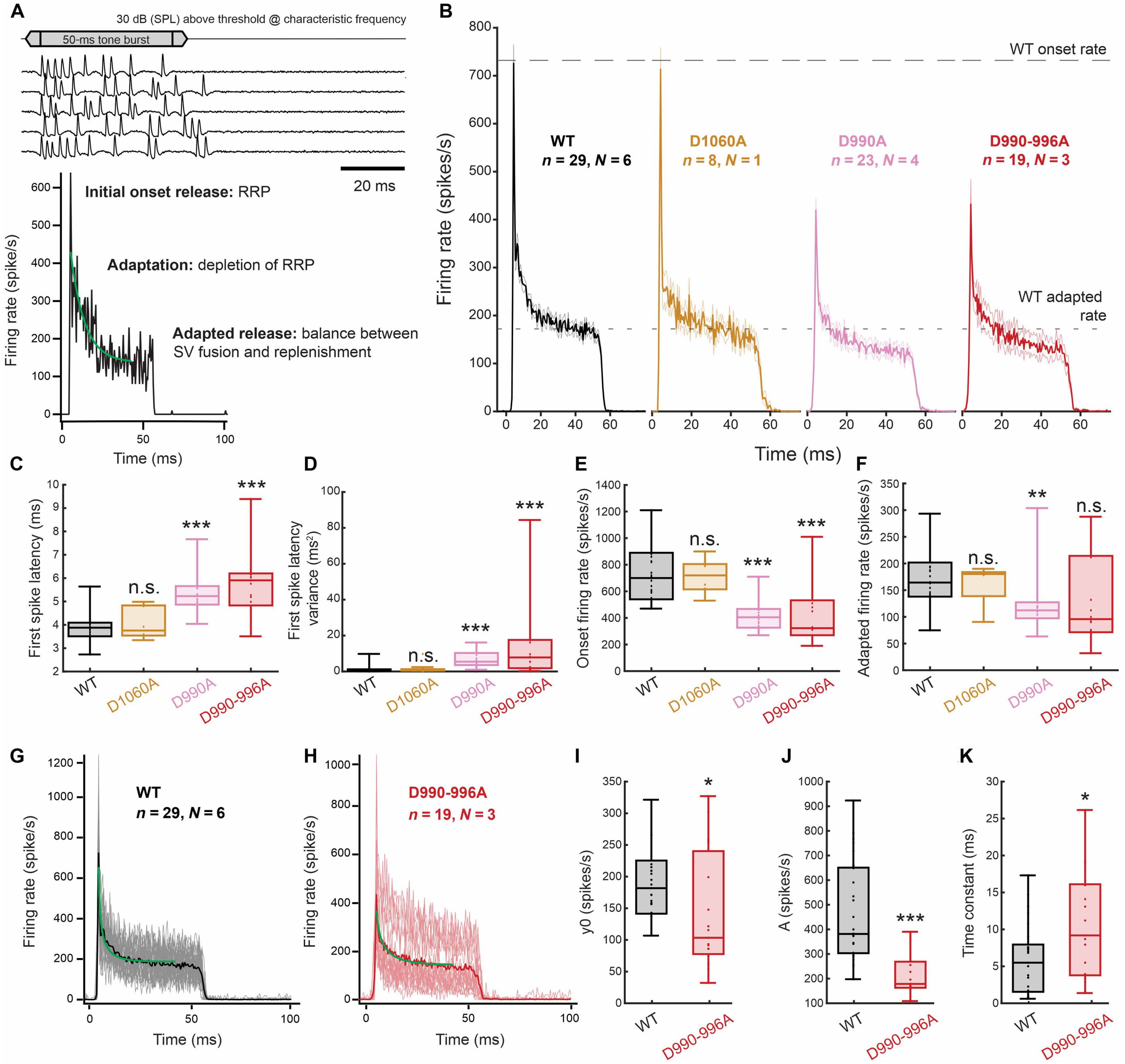

Fig. 7. Diminished SGN onset firing and slowed spike rate adaptation indicate reduced SV release probability. (**A**) Example wild-type SGN spike traces (top) recorded in response to 50-ms tone bursts at characteristic frequency, 30 dB (SPL) above threshold, and resulting peristimulus time histogram (PSTH) of the SGN firing rates across 200 repetitions (bottom). Single exponential fitting of the spike rate adaptation is shown in green. (**B**) PSTHs recorded as depicted in (A) for *Otof*^{wt} (black, $N = 6$, $n = 29$), homozygous *Otof*^{D1060A} (yellow $N = 1$, $n = 8$), *Otof*^{D990A} (magenta, $N = 4$, $n = 23$), and *Otof*$^{D990A\text{-}996A}$ (red, $N = 3$, $n = 19$) mice. Mean traces are displayed with SEM indicated by fainter lines. (**C**) Median first spike latency, (**D**) first spike latency variance, (**E**) onset firing rate, and (**F**) adapted firing rate of the SGNs shown in (B). (**G** to **K**) Single exponential fitting of individual PSTHs of (G) *Otof*^{wt} and (H) *Otof*$^{D990\text{-}996A}$ SGNs for which average traces are displayed [average traces are also shown (B)] with the averaged fits shown in green. Individual PSTHs are shown as fainter lines. (**I**) Plateau of the fit, y0, (K) the amplitude of the adapting component, and (J) the time constant of adaptation for the fitted PSTHs shown in [(G) and (H)]. Fitting was done in Igor Pro. Data in [(C) to (F) and (I) to (K)] are shown as box and whisker plots with data points overlaid, median, 25th and 75th percentiles (box), and 10th and 90th percentiles (whiskers) displayed. Two sample comparisons were performed using *t* test or the Wilcoxon signed-rank test. Significant differences are indicated as $*P < 0.05$, $**P < 0.01$, and $***P < 0.001$. WT, wild type.

directly affected by the otoferlin mutations. Assuming unaltered SGN excitability in the *Otof* mutants, we can use onset firing rate, first spike latency, and its variance and short-term spike rate adaptation to comparatively assess the rate of initial SV-release and kinetics of RRP depletion, respectively [(*29*), review in (*64*, *67*, *69*)]. Hence, while the readout is indirect, we have good access to the key parameters of RRP dynamics in vivo: Onset firing reports initial SV release that is codetermined by release probability and the size of RRP, the time constant of short-term spike rate adaptation approximates that of RRP depletion, which is governed by the rate constants of release (i.e., reporting release probability), and of SV replenishment (*64*). Last, the adapted firing rate reflects sustained release, which is governed by balanced SV fusion and SV replenishment and also requires sustained Ca^{2+} influx (*64*). To date, *Otof* missense mutants studied at the single SGN level showed strong firing rate adaptation lending support to a critical role of otoferlin in SV replenishment (*27*, *37*).

Onset firing was significantly reduced, and first spike latency and its variance were increased in $Otof^{D990A}$ and $Otof^{D990\text{-}996A}$ mutants, but not in $Otof^{D1060A}$ mutants, which indicates a reduced initial SV release rate at $Otof^{D990A}$ and $Otof^{D990\text{-}996A}$ AZs (Fig. 7, B to E). Spike rate adaptation kinetics reflects both neural refractoriness and short-term synaptic depression [e.g.. (*24*)], which can be more precisely described by fitting a double exponential function or a model to it. On the assumption that the impact of refractoriness will be comparable for SGNs of both genotypes, we chose the simplest fit function to approximate the kinetics of RRP depletion. We observed a slower adaptation kinetics in $Otof^{D990\text{-}996A}$ mutants (Fig. 7, G, H, and K), which indicates a reduction of SV release probability and/or of SV replenishment. The fitting of $Otof^{D990\text{-}996A}$ SGN firing further indicated less adapted firing (Fig. 7I) and a diminished amplitude of the adapting component (Fig. 7J). When approximating steady-state release by the adapted firing rate, it was smaller in $Otof^{D990A}$ mutants ($P = 0.001$) but not significantly changed in $Otof^{D990\text{-}996A}$ and $Otof^{D1060A}$ mutants (Fig. 7F), contrasting other *Otof* mutants, e.g., the $Otof^{I515T}$ and $Otof^{D1772G}$ (*Pachanga*) mutants that showed massively reduced adapted firing. We take this to indicate largely intact SV replenishment during ongoing stimulation at IHC synapses of $Otof^{D990\text{-}996A}$ and $Otof^{D1060A}$ mutants, while we cannot currently explain the mild but significant reduction in $Otof^{D990A}$ mutants. Therefore, we propose that reduced release probability, rather than a reduced RRP size due to impaired SV replenishment, is the primary cause of the sound encoding deficit in these C_2D mutants. This notion was further supported by the fact that onset and steady-state firing rate of all three mutants did not change with the stimulation rate beyond what we found in WT SGNs (10 and 0.5 Hz; fig. S14). This again sets apart the C_2D mutants from other *Otof* mutants (*27*, *37*). In summary, our in vivo analysis suggests that the altered Ca^{2+} binding of otoferlin impairs sound encoding by reducing release probability.

To directly assess Ca^{2+}-triggered IHC exocytosis, we turned to patch-clamp recordings of exocytic membrane capacitance changes (ΔC_m) in response to depolarization that elicits maximal Ca^{2+} influx (to −17 mV; Fig. 8, A to O). We chose perforated-patch recordings to minimize rundown of Ca^{2+} current and exocytosis in these experiments typically lasting for ≥10 min (*57*, *70*). Ca^{2+} influx was not significantly altered in voltage dependence and amplitude in IHCs of all three *Otof* mutants (Fig. 8 and fig. S15). We found a significant reduction of ΔC_m elicited by brief (20-ms) depolarizations that tap initial release from the RRP in $Otof^{D990A}$ (3.35 ± 0.96 fF versus 7.77 ± 1.01 fF for littermate control, $P = 0.0047$; Fig. 8H) and $Otof^{D990\text{-}996A}$ (3.29 ± 0.70 fF versus 8.36 ± 0.74 fF for littermate control, $P = 0.00007$; Fig. 8M) IHCs as compared to IHCs obtained from WT littermates.

ΔC_m in response to longer stimuli in $Otof^{D990A\text{-}996A}$ IHCs was smaller than those of littermate controls, while those of $Otof^{D990}$ and $Otof^{D1060A}$ IHCs did not differ from their controls (Fig. 8, D, I, and N). Sustained exocytosis, approximated as previously introduced (*69*) [difference in ΔC_m between 100 and 20 ms of stimulation divided by interval (80 ms) in SVs/ms], assuming a SV capacitance of 40 aF (*71*), was not significantly reduced in any of the C_2D mutants (Fig. 8, E, J, and O). This is consistent with the largely unaltered adapted SGN firing rates in vivo.

Last, we examined the apparent Ca^{2+} dependence of RRP exocytosis of $Otof^{D990A}$ IHCs when gradually reducing the Ca^{2+} influx elicited by 20-ms depolarizations (to −17 mV) by slow bath perfusion of Zn^{2+} (Fig. 8P) (*22*, *24*), which causes a rapidly flickering channel block (*72*). Fitting the relationship of normalized ΔC_m (≤50%, to avoid saturation of the function by RRP depletion) and the integrated normalized Ca^{2+} influx (Q_{Ca}, 70%) by a power function, we observed that the Ca^{2+} cooperativity of RRP exocytosis dropped to a power m of 0.8 ± 0.7 compared to 2.0 ± 0.4 for $Otof^{wt}$ IHCs (Fig. 8, Q and R).

In summary, we found that substituting key top-loop aspartates of the C_2D domain by alanine impaired IHC exocytosis primarily by reducing the release probability. Based on this finding and the loss of Ca^{2+} cooperativity of IHC exocytosis, we indicate that, as previously suggested, otoferlin is the Ca^{2+} sensor of SV fusion in IHCs.

DISCUSSION

We present a structure-function analysis of otoferlin, a deafness gene product, for which the first gene replacement therapy of the inner ear is currently in clinical trials. Cryo-EM structures of lipid-free and nanodisc-bound otoferlin and MD simulations elucidate the structural basis of Ca^{2+}- and lipid-binding by otoferlin. Specifically, we show that C_2C, C_2D, C_2F, and C_2G bind Ca^{2+} by their top-loops but differ in the number of Ca^{2+} ions coordinated and the contribution of anionic phospholipids. Toward deciphering Ca^{2+}-triggered SV fusion at IHC synapses, we tested the "Ca^{2+} sensor of SV fusion hypothesis" by multiscale physiology in mouse mutants with altered Ca^{2+} binding to C_2D. Overall, the solved structures, MD simulations, and in vivo analyses allowed us to advance a model for otoferlin functions in tethering and docking of SVs (Fig. 9A), indicated a Ca^{2+}-sensor of SV-fusion role, and provided the molecular basis for interpretation of human *OTOF* mutations (Fig. 9, B to D).

Structure-informed progressive alteration of Ca^{2+} binding by the C_2D domain of otoferlin led to increasing deficits of synaptic sound encoding. Mechanistically, we observed a reduced release probability and a loss of Ca^{2+} cooperativity of RRP exocytosis. This is reminiscent of the findings in hippocampal neurons of mouse mutants with impaired Ca^{2+} binding by synaptotagmin 1 (*32*) and supports the hypothesis that otoferlin is the Ca^{2+} sensor of SV fusion in IHCs. Our present study extends a series of experiments manipulating the Ca^{2+} binding sites of individual C_2 domains of otoferlin. The functional deficits obtained for the three C_2D mutants turned out to be mild, particularly when compared to mutants of C_2F [previously referred to as C_2E, (*34*)] and C_2G [previously referred to as C_2F, (*53*)]. A previous analysis of $Otof^{D515\text{-}517A}$ mice (*31*) with altered Ca^{2+} binding of C_2C revealed reduced SV replenishment, which might indicate that

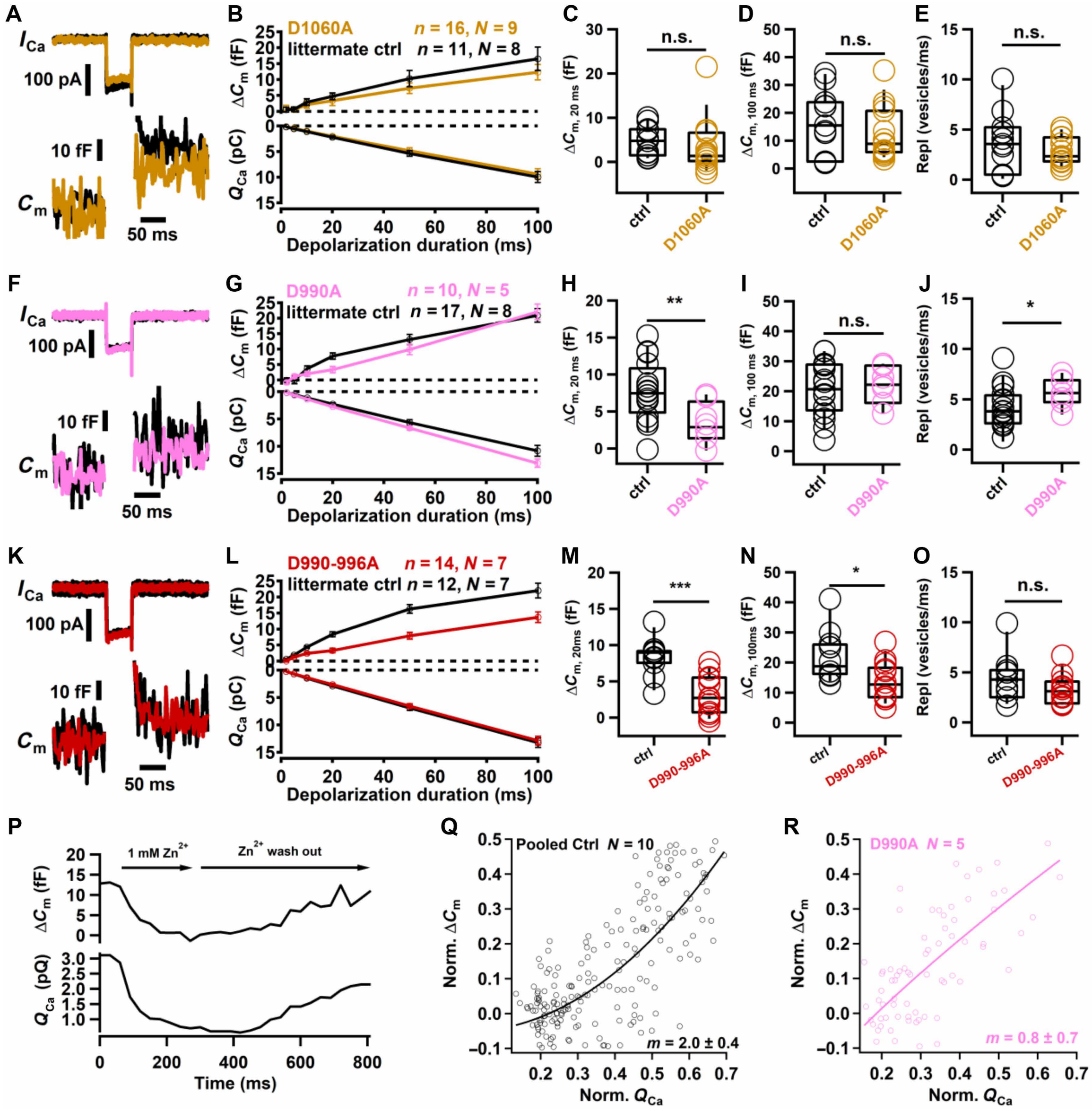

Fig. 8. Impaired synchronous exocytosis and linearization of the Ca^{2+} dependence of IHC exocytosis. (**A**, **F**, and **K**) Exemplary membrane capacitance changes (ΔC_m, top) and Ca^{2+} currents (bottom) elicited by 50-ms depolarizations to −17 mV in IHCs of $Otof^{wt}$ (black), homozygous $Otof^{D1060A}$ (yellow), $Otof^{D990A}$ (magenta), and $Otof^{D990\text{-}996A}$ (red) mice. (**B**, **G**, and **L**) ΔC_m (top) and integrated Ca^{2+} current integral (Q_{Ca}, bottom) elicited by depolarizations to −17 mV for different durations in IHCs, same color code, and numbers of IHCs and mice as in (A), (F), and (K). (**C**, **H**, and **M**) RRP exocytosis reported by $\Delta C_{m,20ms}$ (20 ms depolarizations to −17 mV), same color code and numbers of IHCs and mice as in (A), (F), and (K). (**D**, **I**, and **N**) Exocytosis reported by $\Delta C_{m,100ms}$ (100-ms depolarizations to −17 mV), same color code, and numbers of IHCs and mice as in in (A), (F), and (K). (**E**, **J**, and **O**) Maximal rate of sustained exocytosis (ΔC_m per ms during 20 and 100 ms of depolarization to −17 mV), same color code, and numbers of IHCs and mice as in in (A), (F), and (K). (**P**) The exemplary estimation of the apparent Ca^{2+} dependence using rapid flicker blocking by 1 mM Zn^{2+} in a control IHC: ΔC_m (top) and Q_{Ca} (bottom). (**Q** and **R**) Scatter plots of normalized ΔC_m versus Q_{Ca} of control (Q) and mutant [R, $Otof^{D990A}$ ($n = 5$)] and power function [norm. $\Delta C_m = A(\text{norm. } Q_{Ca})^m$] fits to the pooled data. Data in [(B), (G), and (L)] are shown as mean SEM, and box and whisker plots with data points overlaid and show median, mean, 25th and 75th percentiles (box), and 10th and 90th percentiles (whiskers). Two sample comparisons were performed using *t* test or the Wilcoxon signed-rank test. Significant differences are indicated as *$P < 0.05$, **$P < 0.01$, and ***$P < 0.001$.

Ca^{2+}-bound C_2C pioneers the contact of otoferlin to the target membrane for SV tethering (Fig. 9). In contrast, we found SV replenishment to be largely unaltered in IHCs of the newly described C_2D mutants (Figs. 7 and 8). This allowed us to isolate the "Ca^{2+} sensor of SV fusion function" of otoferlin and to conclude that Ca^{2+} binding to C_2D is dispensable for Ca^{2+}-dependent SV replenishment, consistent with the domain weakly engaging lipid bilayers (Figs. 2 and 5). Future studies will be required to elucidate how the disrupted Ca^{2+} binding of C_2D collapses the supralinear Ca^{2+} dependence that likely involves Ca^{2+} binding to further C_2 domains. The analysis of Ca^{2+} binding by isothermal titration calorimetry and highly resolved paired pre- and postsynaptic recordings combined with Ca^{2+} uncaging will be required to further elucidate this process. Ideally, this will also involve mutants combining Ca^{2+}-binding deficiencies in multiple C_2 domains.

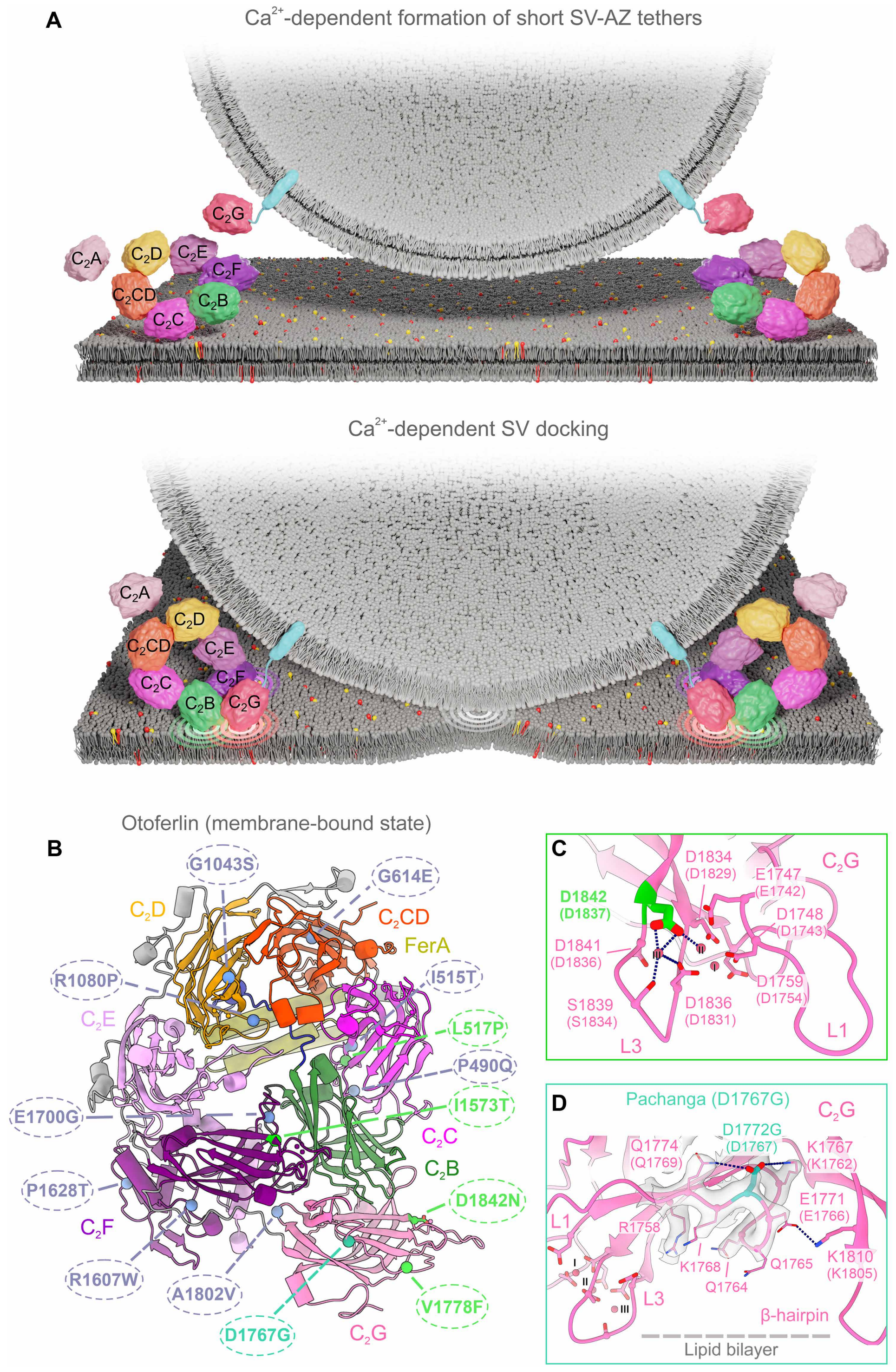

Fig. 9. Model of otoferlin-mediated SV docking and predicted impact of *OTOF* mutations. (**A**) Model of otoferlin-mediated SV docking inspired by structural and functional data. For simplicity and lack of sufficient understanding for the precise molecular mechanism of fusion, we have not aimed to draw-in SNAREs and sketch the fusion step. (**B**) Selected *OTOF/Otof* missense mutations with amino acid substitutions mapped onto the membrane bound structure of human otoferlin. Amino acids in mouse sequence are given in parentheses. Dark blue font indicates mild hearing impairment, and green font indicates moderate or severe hearing impairment (table S3). (**C** and **D**) Structural prediction of mutations affecting C_2G. The D1842 (mouse D1837) residue of C_2G coordinates two Ca^{2+} ions (C) and is the target of a human missense mutation (D1842N). D1842 has been previously substituted in a mouse mutant (D1837A), likely the functional equivalent of the human D1842N, and its mutation abolished Ca^{2+}-triggered exocytosis at IHC synapses. The D1767 residue (D) is involved in a network of polar contacts, bridging the membrane-facing concave surface of C_2G and the lipid-binding β-hairpin motif. The *Pachanga* mice, bearing the D1767G substitution, are profoundly deaf and exhibit impaired SV replenishment for fusion but normal Ca^{2+}-triggered exocytosis.

Otoferlin had long been thought to operate as a synaptotagmin-1–like Ca^{2+} sensor at the afferent synapses of the inner ear (*6*). However, otoferlin and synaptotagmin-1 do not appear to be functionally equivalent (*10*, *11*). In our cryo-EM structures, otoferlin adopts a significantly more complex architecture (Fig. 1E). Compared to the two loosely connected C_2A and C_2B of synaptotagmin-1, the seven resolved C_2 domains of otoferlin, four of which bind Ca^{2+}, establish multiple tertiary interfaces, are arranged in a ring-like manner, and form a composite membrane-binding surface (Fig. 1E and 9A). An additional layer of complexity arises from the ability of otoferlin to toggle between discrete states in a Ca^{2+}-dependent manner as it engages lipid membranes (Figs. 4 and 9A). These organization principles, recently found for all ferlin structures (*44*), this study) raise an important question: Why do IHC synapses use a Ca^{2+} sensor for SV fusion with such an intricate organization?

One possibility is that the complex, multidomain structure enables otoferlin to combine roles previously attributed to multiple individual factors at CNS synapses, many of which are likely dispensable or absent from mature IHCs (*8*, *10*–*13*, *73*): IHC exocytosis does not appear to rely on Munc13, Munc18, or complexin-1 as SV-priming proteins or regulators of SV fusion. At CNS synapses, Munc18 and Munc13 promote the assembly of the *trans*-SNARE complexes (*74*–*76*) and are essential for both spontaneous and Ca^{2+}-triggered transmitter release (*76*, *77*), while complexin-1 controls the Ca^{2+}-evoked SV fusion by modulating the final *trans*-SNAREs zippering (*78*, *79*). Similar to otoferlin, Munc13 has a complex multidomain organization, and same as in otoferlin, its C_2 domains have been ascribed roles ranging from SV tethering and organization of the AZ membrane to Ca^{2+}-dependent regulation of tight SV docking for fusion (*14*). While the role and identity of SNAREs at IHC synapses remains to be elucidated, we speculate that otoferlin—in a SNARE-dependent scenario—might chaperone and modulate the assembly of *trans*-SNARE complexes for SV fusion, much like Munc18 and complexin-1 at CNS synapses do. This might involve additional structural motifs, such as the FerA domain, the anchor loop of C_2E, or the many C_2 domain linkers, which are not involved in membrane interactions required for SV docking. An alternative possibility is that the specialized multi-C_2 domain structure of otoferlin enables a more extensive AZ remodeling than can be achieved by synaptotagmin-1, thereby potentially lowering the energy barriers enough to allow SV fusion to occur independently of SNAREs. Supporting the latter hypothesis, our cryo-EM structures and simulations implicate all the modeled C_2 domains in membranes interactions to varying extents. In particular, structural elements of C_2B, C_2C, and C_2F-C_2G deeply insert into the lipid bilayer in a concerted manner, which may alter the bilayer structure and induce membrane curvature changes.

Apart from a Ca^{2+} sensor function, a role of otoferlin in preparing SVs for fusion has been proposed based on ultrastructural and physiological mouse analyses in mutant mice (*12*, *27*, *36*, *37*). Specifically, the electron tomography of IHC synapses of *Otof*$^{-/-}$ mice revealed a lack of short tethers (<20 nm) connecting membrane-proximal SVs to the AZ membrane. These short "tethers" are well compatible with the dimensions of SV-resident otoferlin (~10 nm) that might mediate initial contacts with the AZ membrane via its Fer^{core} module, for example, through the C_2C domain (model in Fig. 9A). Consistent with such a role, the C_2C domain showed extensive lipid interactions in MD simulations (Fig. 5) and interacted with lipid membranes in myoferlin structures (*44*). We postulate that SVs then proceed to docking by progressive membrane interaction of otoferlin. In the nanodisc-bound cryo-EM structures, the C_2F and C_2G domains establish Ca^{2+}-sensitive contact interfaces with the lipid bilayer and undergo large-scale conformational rearrangements upon membrane binding (Figs. 4 and 5). As these C-terminal domains are connected to the transmembrane helix through a short linker, their concomitant interaction with the AZ membrane in response to Ca^{2+} influx would then likely (i) tilt the ferlin ring from the vertical tethering position to a planar arrangement, exposing its multidomain lipid interface, and (ii) shorten the distance between the tethered SVs and the AZ membrane for establishing tight docking (Fig. 9A). We note that, vice versa, AZ membrane–resident otoferlin (*6*, *29*, *37*) might engage with the SV membrane (not drawn in Fig. 9A), dependent on the precise, yet currently unknown, lipid composition of the SV.

The precise sequence of Ca^{2+}- and lipid-binding events and accompanying conformational changes in otoferlin remain to be investigated by further cryo-EM studies, MD simulations and potentially super-resolution light microscopy. We hypothesize that SVs get attracted to the AZ membrane via otoferlin-independent longer-range tethering mechanisms (*12*) and proceed to short-range tethering and docking upon progressive engagement of otoferlin with the AZ membrane (Fig. 9A). Otoferlin might sample the open and closed conformation and—upon contacting the membrane via SV-distal C_2C-C_2E domains—transition (Fig. 9A) to the closed configuration pulling the SV and AZ membranes closer to each other by the SV-proximal C_2F and C_2G engaging with the AZ membrane (Fig. 9D). Consistent with such a scenario, the electron tomography of stimulated IHC AZs of *Pachanga* mice (*27*, *80*), carrying the *Otof*^{D1772G} mutation (D1767G in mice) in C_2G, showed an increase of multiply tethered and docked SVs (*81*). Our structures indicate that the *Otof*^{D1772G} mutation modulates the network of hydrogen bonds between the C_2G core and the C_2G β-hairpin (depicted in Fig. 9, B and D) and, therefore, likely affects the tight C_2G interaction with the membrane and the interface of C_2G with C_2F. While the Ca^{2+}-mediated electrostatic interactions of the C_2F and C_2G top loops with the membrane might allow for loose SV docking, additional contacts between C_2C-C_2E and the membrane could be required for tight docking of the SV and high fusion probability (*56*).

More than 200 human pathogenic and likely pathogenic mutations in *OTOF* have been linked to the nonsyndromic autosomal recessive deafness DFNB9 or mild to moderate hearing impairment (*45*). The high-resolution otoferlin structures, in different conformational states, allowed us to map the most frequent mutations in three-dimensional (3D) and explain their structural consequences (Fig. 9B and table S3). We observed that these pathogenic substitutions affect all Ca^{2+}-binding C_2 domains of otoferlin (C_2C, C_2D, C_2F, and C_2G), whereas the C_2B and C_2E, which engage lipid membranes but do not coordinate Ca^{2+} ions, are not mutation targets. One mutation (G614E) maps to the C_2CD of the Fer^{core} module, an otoferlin domain important for maintaining the structural organization of the protein and also present in other ferlins (*44*). However, we could not pinpoint obvious mutational hotspots, and except p.D1842N, the analyzed substitutions do not interfere directly with Ca^{2+} binding by otoferlin's C_2 domains. The otoferlin structure predicts the human *OTOF* variant p.D1842N (Fig. 9, B and C, and table S3) (*82*) to disrupt the binding of two Ca^{2+} by the C_2G top loops, which we interpret to interfere with SV docking (Fig. 9, B and C, and table S2). Mouse mutagenesis targeting C_2G Ca^{2+} binding (*Otof*^{DDA}, p.D1836/1837A

in mouse, and p.D1841/1842A in human) revealed deafness due to a complete loss of IHC exocytosis despite substantial (~60%) basolateral otoferlin levels (*53*). We conclude that the most pathogenic otoferlin mutations may compromise the structural integrity of otoferlin, likely affecting the fold of the individual C_2 domains (i.e., by disrupting the packing of β sheets) or interfering with their tertiary interfaces.

Limitations of the study

Our structural work used a truncated construct lacking the nonessential N-terminal C_2A domain and the C-terminal transmembrane domain of otoferlin (residues 216 to 1931). The engineered construct encompasses all functionally relevant Ca^{2+}- and lipid-binding motifs, including the ordered C_2A-C_2B linker, and enabled us to obtain near-atomic structures of otoferlin in the membrane-bound state. A crystal structure of the missing C_2A domain is available (*41*), and in contrast to type I ferlins, this flexible domain in otoferlin lacks Ca^{2+}- and membrane-binding activity (*10*, *30*). Although we believe that our structural insights largely apply to the full-length protein, we cannot formally exclude minor structural differences, which should be addressed in future studies.

Since mice were anesthetized with isoflurane for in vivo recordings, we note that we may have underestimated the effect of the *Otof* mutations on spontaneous firing. Isoflurane causes lower spontaneous firing rates (*83*), likely due to inhibition of $Ca_V1.3$ channels of IHCs. Moreover, the analysis of Ca^{2+} cooperativity of IHC exocytosis summed over all IHC synapses and was limited to a small IHC sample of only one of the *Otof* C_2D mutants. Last, potential indirect effects of the C_2D substitutions such as on the interactions of otoferlin with other proteins, e.g., $Ca_V1.3$ channels, were not explicitly examined in this study.

The full elucidation of the molecular mechanisms underlying otoferlin-dependent SV release at IHC synapses will require integration of further molecular, structural, and functional data. Questions remaining include: (i) How reversible are the tethering and docking reactions? (ii) Is IHC exocytosis SNARE dependent? (iii) Which proteins compose the fusion machinery and what is their stoichiometry?

MATERIALS AND METHODS

Animals

Generation of Otof mutant mice using CRISPR-Cas9

Three knock-in mouse lines *Otof*^{D1060A} (*Otof*^{em6Bros}), *Otof*^{D990A} (*Otof*^{em5Bros}), and *Otof*$^{D990A\text{-}D996A}$ (*Otof*^{em4Bros}) were generated by site-directed CRISPR-Cas9 mutagenesis. Superovulated C57BL/6 N females were mated with C57BL/6N males and fertilized eggs were collected. In-house prepared CRISPR reagents [hCAS9_mRNA, single guide RNA (sgRNA), and either dsDNA or long oligonucleotides were used as repair templates containing the desired mutation] or preformed Cas9_sgRNA RNP complexes were microinjected into the pronucleus and the cytoplasm of zygotes at the pronuclear stage using Eppendorf Femtojet. Note that all nucleotide based CRISPR-Cas9 reagents (sgRNAs and hCAS9_mRNA) were used as RNA molecules and were not plasmid-coded. In this way, we intended to reduce the likelihood for off-target effects, as RNA-based reagents are only short-lived in contrast to plasmid-coded reagents.

The corresponding verified mutated sequences were as follows:

Otof^{D1060A} (5′-ggcaaagccg**CT**ttcatgggccg*C*accttcgccaagcccctggtgaagatggcagatgaagcatactgcccacctcgcttcccgccgcagcttgagtactaccagatctaccgaggcagtgccactgccggagacctactggctgccttcgagctgctgcag-3′)

The sequence shown covers 3303 to 3464 base pairs (bp) (Exon27) of GenBank acc. no. NM_ 001100395, *Mus musculus* otoferlin (Otof) transcript variant 1 mRNA. The nucleotides used to introduce the D1060A missense mutation are marked in bold, and the nucleotide used to introduce the silent mutation is marked in italics.

Otof^{D990A} (5′-agaagcaagccttccagctccgagcacacatgtatcaggcccgaagcctctttgctgctg**C**cagcag*C*gg*C*ctctctgatccctttgcccgtgtcttcttcatcaaccagagccaatgcactgag-3′)

The sequence shown covers 3043 to 3167 bp (Exon25) of GenBank acc. no. NM_ 001100395, *M. musculus* otoferlin (Otof) transcript variant 1 mRNA. The nucleotide used to introduce the D990A missense mutation is marked in bold, and the nucleotides used to introduce silent mutations are marked in italics.

Otof$^{D990A\text{-}D996A}$ (5′-agaagcaagccttccagctccgagcacacatgtatcaggcccgaagcctctttgctgctg**C**cagcag*C*gg*C*ctctctg**C**tccctttgcccgtgtcttcttcatcaaccagagccaatgcactgag-3′)

The sequence shown covers 3043 to 3167 bp (Exon25) of GenBank acc. no. NM_ 001100395, *M. musculus* otoferlin (Otof) transcript variant 1 mRNA. The nucleotides used to introduce the D990A and D996A missense mutations are marked in bold, and the nucleotides used to introduce silent mutations are marked in italics.

Experimental mice and husbandry

Mice of either sex, aged between 2 and 18 weeks, were used for experiments. Mice were group-housed with up to five mice per ventilated cage, provided with environmental enrichment (cardboard rolls and wood wool), fed an ad libitum diet of standard chow, and kept on a 12-hour light/12-hour dark cycle. Mice were housed in the animal facility of the Max Planck Institute for Multidisciplinary Sciences or in the Central Animal Facility of the University Medical Center Göttingen. All experiments complied with national animal care guidelines and were approved by the University of Göttingen Board for Animal Welfare and the Animal Welfare Office of the State of Lower Saxony (AZ19/3133 and AZ19/3134).

Characterizing mouse otoferlin

Expression and purification of mouse otoferlin

For insect cell expression, codon-optimized mouse otoferlin (residues 216 to 1931) was subcloned by ligation-independent cloning into a modified pFastBac vector backbone (primers: 5′-TACTTCCAATCCAATGCAATGGAAGACCTCGACCACTTGGCT-3′ and 5′-TTATCCACTTCCAATGTTATTACTTTTCCAAGGGGTCAGGCTCG-3′) in frame with an N-terminal twin-StrepII tag, cleavable with the HRV-3C (human rhinovirus 3C) protease. The D1829A and D1831A substitutions in the Ca^{2+}-binding sites of the C_2G domain were introduced by "around-the-horn" polymerase chain reaction–based site-directed mutagenesis (*84*) with the following 5′-phosphorylated DNA primers: 5′-CAGATCTGGGCCGCCGCCCACTTCTCAGCTG-3′ and 5′-GAGAGTCAGCCTGGCAGGGATC-3′. All cloning primers were synthesized by Microsynth Seqlab GmbH (Göttingen), and all constructs were verified by Sanger sequencing (Microsynth Seqlab GmbH, Göttingen).

Sf9 insect cells (*Spodoptera frugiperda*) were cultured in ESF 921 (Expression Systems, catalog no. 96-001-01), and recombinant baculoviruses were prepared as described previously (*84*–*87*). For large-scale expression of otoferlin (216 to 1931), Sf9 suspension cultures, grown at a density of ~1.0×10^6 cells/ml, were infected with V1 baculoviruses at a ~1:100 ratio, resulting in cell proliferation arrest after 24 hours. Infected cells were harvested after 72 hours and

washed with 1× phosphate-buffered saline (PBS), and the cell pellets were stored at −80°C.

Wild-type and D1829A/D1831A otoferlin (216 to 1931) were purified from insect cells by adapting the recently established dysferlin and myoferlin protocols (*44*). Briefly, infected Sf9 cells from 1-liter culture were resuspended in the lysis buffer [50 mM Hepes-KOH (pH 7.5), 300 mM KCl, 10% (v/v) glycerol, 4 mM dithiothreitol (DTT), 0.2% (v/v) Triton X-100, 1x EDTA-free cOmplete protease inhibitors cocktail (Roche, catalog no. 11836170001), and cell pellet (10 ml/g)] and lysed by sonication on ice. The lysate was cleared by centrifugation at 15,000 rpm for 1 hour at 4°C in a JA-18 rotor (Beckman Coulter) and filtered (0.8-μm Minisart, Sartorius, catalog no. 16592). The otoferlin samples were then bound in batch to 4 ml of Strep-Tactin XT 4FLOW high-capacity resin (50% slurry, IBA Lifesciences, catalog no. 2-5030-010) for 1 hour at 4° to 8°C. The affinity resin was collected and washed with the lysis buffer and the binding buffer [50 mM Hepes-KOH (pH 7.5), 150 mM KCl, 5% (v/v) glycerol, and 2 mM DTT]. The captured samples were eluted with the elution buffer [50 mM Hepes-KOH (pH 7.5), 200 mM KCl, 5% (v/v) glycerol, 2 mM DTT, 1 mM EDTA, and 60 mM biotin) and further purified by anion exchange chromatography [5-ml HiTrap Q Sepharose HP column, Cytiva, cat#17–1154-01). Following sample binding, the column was washed with buffer A [25 mM Hepes-KOH (pH 7.5), 150 mM KCl, 5% (v/v) glycerol, and 1 mM TCEP (tris(2-carboxyethyl)phosphine)], and the samples were eluted using a 0 to 50% linear gradient over 50 ml, formed between buffer A and buffer B [25 mM Hepes-KOH (pH 7.5), 1 M KCl, 5% (v/v) glycerol, and 1 mM TCEP]. The pure otoferlin fractions were concentrated by ultrafiltration to ~4.3 to 4.7 mg/ml (WT) or ~3.3 mg/ml (the D1829A/D1831A mutant), frozen in liquid nitrogen, and stored at −80°C.

NanoDSF-based characterization of mouse otoferlin

In a typical nanoDSF assay (*44*), used to assess the ability of otoferlin (216 to 1931), WT, or D1829A/D1831A mutant, to bind Ca^{2+} in the presence of liposomes (large unilamellar vesicle, LUVs, fig. S1D), the purified protein sample at a 1.6 to 2 μM final concentration was preincubated with 1 mM LUVs for 10 min at room temperature. The LUVs were prepared as described in (*44*) and comprised 20 mol % DOPS, 20 mol % DOPS, and 5 mol % PI(4,5)P_2 or lacked anionic phospholipids. In the next step, 5 μl of protein-liposome sample (at 1.6 to 2 μM) was mixed with 5 μl of Chelex-treated assay buffer [25 mM Hepes-KOH (pH 7.5) and 150 mM KCl] supplemented with increasing concentrations of $CaCl_2$ (0 to 10 mM). Following an additional 10-min incubation at room temperature, the emission intensity at 350 and 330 nm was measured using a Prometheus NT.48 instrument (NanoTemper Technologies) as the temperature was increased from 20° to 95°C at 1°C/min. The melting temperatures (T_m) were calculated with the PR.ThermControl v2.3.1 software, and the $[Ca^{2+}]_{1/2}$ values were estimated in OriginPro 2018 by non-linear regression fitting to a modified Hill function (*44*).

Liposome-binding assays

To investigate the ability of otoferlin (216 to 1931) to bind lipid bilayers in vitro (*88*), 50 μM Dansyl-labeled LUVs (containing 5 mol % 18:1 Dansyl PE) were mixed with 0.25 μM purified protein sample in the assay buffer [25 mM Hepes-KOH (pH 7.5) and 150 mM KCl], which was supplemented with 250 μM $CaCl_2$ or $MgCl_2$, resulting in a final reaction volume of 115 μl (fig. S1G). The tested liposomes contained 25 mol % DOPS, 25 mol % DOPS, and 5 mol % PI(4,5)P_2 or were devoid of anionic phospholipids (*44*). Following incubation for 5 min at room temperature, the emission spectra were taken between 450 and 560 nm at a 284-nm excitation wavelength (3-nm slits, 0.1-s integration time) using a Fluorolog 3 spectrofluorometer (Horiba Jobin Yvon). The otoferlin (216 to 1931)–D1829A/D1831A samples were measured in a similar manner. The relative protein-to-membrane Förster resonance energy transfer (FRET) efficiency was calculated as follows: $\mathrm{rFRET} = (I - I_{\min})/(I_{\max} - I_{\min})$, where I represents the average emission intensity of the sample at 518 to 522 nm, $I_{\min}$ is the intensity of the protein-free blank sample, and $I_{\max}$ is the maximum Dansyl emission of the series. All experiments were repeated at least three times and analyzed in OriginPro 2018. In coflotation assays, purified otoferlin (216 to 1931) samples were mixed with 100-nm LUVs in the presence of 50 μM $CaCl_2$ or $MgCl_2$ and applied to the bottom layer of a Nycodenz step gradient (0%/30%/40%). The final protein and liposomes assay concentrations were ~1 μM and 1 mM, respectively. The Nycodenz gradients were centrifuged at 50,000 rpm for 90 min in a TLS-55 rotor (Beckman Coulter), harvested in 20-μl fractions, and analyzed by SDS–polyacrylamide gel electrophoresis.

Mass photometry characterization of otoferlin

Before mass photometry, purified otoferlin (216 to 1931) was applied to a Superose 6 3.2/300 Increase column, equilibrated in the assay buffer [25 mM Hepes-KOH (pH 7.5), 150 mM KCl, 5% (v/v) glycerol, and 1 mM TCEP]. The two otoferlin peak fractions were 10-fold diluted with the assay buffer by adding 1-μl sample to a 12 μl of buffer droplet. Mass photometry measurements were performed with the OneMP mass photometer (Refeyn), and images were acquired with Refeyn AcquireMP software. Mass calibration was carried out by adding 5 μl of NativeMark Unstained Protein Standard (Invitrogen, catalog no. LC0725), diluted 1:200, to a 12 μl of buffer droplet and using the bovine serum albumin (66 kDa), lactate dehydrogenase (146 kDa), and apo-ferritin (480 kDa) peaks. The experiment was repeated four times (two times for each otoferlin fraction).

Cryo-EM structure determination

Cryo-EM sample preparation

The otoferlin (216 to 1931)–lipid nanodisc complex was assembled in vitro by mixing empty MSP2N2 nanodiscs (*52*) with the purified soluble otoferlin construct. Empty nanodiscs—containing 30 mol % DOPS, 10 mol % PI(4,5)P_2 or 25 mol % DOPS and 5 mol% PI(4,5) P_2 acidic phospholipids—were prepared by combining purified His-tagged human MSP2N2 (~3.0 mg/ml; obtained from Cube Biotech, catalog no. 26172) and DDM(n-Dodecyl-β-D-maltoside)-solubilized lipids in a ~1:200 protein-to-lipids ratio. Following overnight dialysis against 2 liter of reconstitution buffer [25 mM Hepes-KOH (pH 7.5), 150 mM KCl, and 1 mM DTT] in the presence of Bio-Beads SM-2 (2.5 g/liter), the nanodiscs were purified by SEC and added in ~2-fold molar excess over otoferlin (216 to 1931) in the presence of 0.5 mM $CaCl_2$. Before cryo-EM grid preparation, the otoferlin (216 to 1931)–nanodisc complex was crosslinked in batch on ice with 0.07% (v/v) glutaraldehyde for 12 min. The reaction was quenched with 50 mM L-lysine and L-arginine, and the sample applied to a Superose 6 10/300 Increase column, equilibrated in the SEC buffer [25 mM Hepes-KOH (pH 7.5), 150 mM KCl, 0.5 mM TCEP, 1.25% (v/v) glycerol, and 0.5 mM $CaCl_2$]. Fractions of the otoferlin (216 to 1931)–nanodisc complex were concentrated by ultrafiltration to A_{280} (absorbance at 280 nm) ~ 0.92 to 1.04 and, following the application of 3 μL sample to plasma-cleaned Quantifoil R1.2/1.3 200 grids (Jena Bioscience, catalog no. X-101-CU200), and vitrified in

liquid ethane-propane (37%:63%) cooled by liquid nitrogen. Grids optimal for data collection were obtained by blotting for 9 s at a blot force of 3 using a Vitrobot Mark IV plunger (Thermo Fisher Scientific), which was operated at 4°C and 100% humidity. The Ca^{2+}-bound lipid-free otoferlin (216 to 1931) samples were prepared in a similar manner, except omitting the nanodisc, and concentrated to A_{280} ~ 0.42 before vitrification. Lipid-free otoferlin (216 to 1931) samples prepared in the absence of Ca^{2+} were stabilized through GraFix, as described for human dysferlin (*44*), buffer-exchanged through ultrafiltration (to the minimal buffer: 25 mM HEPES-KOH, pH 7.5, 150 mM KCl, 0.5 mM DTT, 1.25% (v/v) Glycerol), and concentrated to A_{280} ~ 0.98 before cryo-EM grid preparation.

Cryo-EM data collection and image processing of otoferlin samples

The nanodisc-bound and lipid-free otoferlin (216 to 1931) cryo-EM datasets were acquired on a Titan Krios G4 electron microscope (Collaborative Laboratory and User Facility for Electron Microscopy, Georg-August-Universität Göttingen). The microscope was operated at an accelerating voltage of 300 kV and equipped with a Falcon4i direct electron detector and a Selectris X zero-loss energy filter. Otoferlin cryo-EM movies were acquired with EPU (Thermo Fisher Scientific) at a ×165,000 nominal magnification at the specimen level, resulting in a 0.72 Å/pixel exposure sampling rate. Inelastically scattered electrons were filtered out by setting the energy filter slit to 10 eV. Cryo-EM movies were saved as EER frames following exposure over 2.41 to 2.52 s, which resulted in total fluences of ~37.85 $e^-/Å^2$ [30 mol % PS and 10 mol % PI(4,5)P_2 nanodisc] and ~38.08 $e^-/Å^2$ (25 mol% PS and 5 mol% PI(4,5)P_2 nanodisc) for the nanodisc-bound and ~38.13 $e^-/Å^2$ (Ca^{2+} bound) and ~37.91 $e^-/Å^2$ (Ca^{2+} free) for the lipid-free otoferlin (216 to 1931) datasets (tables S1 and S2). The cryo-EM movies were fractionated in 40 EER fractions during on-the-fly preprocessing [patch motion correction, patch CTF (contrast transfer function) estimation, and dose weighting] with cryoSPARC Live (v4.5 to v.4.7). Otoferlin particles were picked using the Blob Picker and Template Picker, extracted in 360 pixel boxes and 2× binned, before being subjected to supervised 3D classification (Heterogeneous refinement) in cryoSPARC using an ab initio generated reference and four "decoy" volumes. The intact particle sets were further cleaned in 2D, refined in 3D, recentred and reextracted in a 360 pixel box (0.72 Å/pixel) before additional 3D classification and non-uniform refinement in cryoSPARC (figs. S2C, S3C, S4C, and S5D). To resolve the dynamic C_2G in the lipid-free dataset, the otoferlin particles were subjected to additional supervised 3D classification (with six classes) and masked 3D classification (with four classes) in cryoSPARC, which allowed us to identify two C_2G classes (figs. S4C and S5, E and F). For all samples, the final particle sets were subjected to sequential CTF refinement and reference-based motion correction to obtain the final maps. Final otoferlin particles were also refined in 3D with RELION-5.0 (*89*), with Blush regularization switched on, resulting in comparable maps (figs. S3C and S4C). To enable model building, the final maps of lipid-free otoferlin (216 to 1931) were sharpened using LocScale (*90*) or RELION-5.0 (figs. S4F and S5F), and the local resolution of the maps was estimated in cryoSPARC. The continuous flexibility and conformational space of lipid-free otoferlin (216 to 1931) particles were assessed using the 3D variability analysis (3DVA) tool in cryoSPARC, with three modes and at a 6-Å resolution.

Model building and refinement

Model building was initiated from AlphaFold3 predictions of otoferlin (*91*). The AlphaFold3 models were edited by removing low-confidence regions and divided into structural modules (C_2B-C_2D, C_2E, C_2F, and C_2G), which were then individually fit into the overall cryo-EM maps in ChimeraX (v1.8). Otoferlin models were initially refined with ISOLDE (*92*). The resulting structural models were corrected and manually rebuilt in Coot (v0.9.8.5) (*93*) and iteratively refined with phenix.real_space_refine (Phenix v1.19.2-4158 to v1.21.2-5419) (*94*, *95*) with nonbonded_weight set to 1000 for the final refinements. The modeling of bound Ca^{2+} ions and PS headgroups was enabled by $F_o - F_c$ omit maps, calculated with Servalcat (*96*). Geometry restraints for the refinement of the two phosphatidylserine molecules were generated with Grade2 (https://grade.globalphasing.org). All structural models were validated with MolProbity in Phenix. The models and maps were deposited in Protein Data Bank (PDB) and Electron Microscopy Data Bank with the following accession codes: lipid-bound otoferlin [30 mol % PS and 10 mol % PI(4,5)P_2 nanodisc: PDB 9QE2 and EMD-53046; 25 mol % PS and 5 mol% PI(4,5) P_2 nanodisc: PDB 9SEA, EMD-54805; merged datasets: PDB 9SFL and EMD-54827], lipid-free otoferlin (open conformation, class 1: PDB 9SEG and EMD-54809; open conformation, class 2: PDB 9SE5, EMD-54802; closed-like state: 9SI1, EMD-54923; Ca^{2+}-free state: PDB 9SH0, EMD-54883). Structural figures were prepared with ChimeraX (v1.8). Contact interfaces were estimated with PISA (v1.5.2) (*97*). Data collection and refinement statistics are provided in tables S1 and S2.

Immunohistochemistry and imaging

Mice (2 weeks old and 8 weeks old) were deeply anesthetized with CO_2 and euthanized by decapitation for immediate dissection of the cochleae in ice-cold PBS. Fixation was performed by perfusing the cochleae with 4% formaldehyde (in PBS) for 60 min on ice, while for the labeling of synapses, a shorter fixation of 30 min was performed. The organs of Corti were dissected and washed briefly in PBS at room temperature. The blocking and permeabilization of the tissue were performed with goat serum dilution buffer [GSDB: 16% normal goat serum, 450 mM NaCl, 0.3% Triton X-100, and 20 mM phosphate buffer (pH ~ 7.4)] for 1 hour at room temperature. Samples were then incubated with primary antibodies (diluted in GSDB) overnight at 4°C and were washed three times for 10 min in wash buffer [450 mM NaCl, 0.3% Triton X-100, and 20 mM phosphate buffer (pH ~ 7.4)]. This was followed by incubation with secondary antibodies (diluted in GSDB) for 1 hour in a light-protected wet chamber. Last, the samples were washed three times for 10 min in wash buffer before mounting onto glass slides with a drop of fluorescence mounting medium (Mowiol 4-88, Carl Roth, Karlsruhe, Germany) and covered with thin glass coverslips. Images were acquired in confocal mode using an Abberior Instruments Expert Line STED microscope (Abberior Instruments GmbH, Göttingen, Germany). We used lasers at 488, 561, and 640 nm for excitation. 1.4 numerical aperture (NA) 100× or 0.8 NA 20× oil immersion objectives were used. Confocal stacks were acquired using Imspector Software (Abberior Instruments GmbH, Göttingen, Germany; pixel size = 70 nm by 70 nm in *xy*, 200 nm in *z*). The acquired images were *z*-projected with NIH ImageJ software and adjusted for brightness and contrast. Organs of Corti from both mutant mice and corresponding WT mice were always processed in parallel using identical staining protocols, laser excitation powers, and microscope/detector settings. Images were acquired and analyzed with the observer blinded to the genotype.

The following primary antibodies were used: Mouse anti-otoferlin (Abcam, #ab53223) was used to label otoferlin, chicken

anti-parvalbumin (Synaptic Systems, #195006) or rabbit anti-Vglut3 (Synaptic Systems, #135203) was used as a context marker to label IHCs, mouse anti-ctbp2 (BD Biosciences, #612044), and rabbit anti-homer1 (Synaptic Systems, #160002) were used to label pre- and post-synapse, respectively. The following secondary antibodies from goat were used: STAR580 conjugated anti-mouse (Abberior, #2-0002-005-1), Alexa488-conjugated anti-chicken (Invitrogen, #A11039), Alexa647-conjugated anti-rabbit (Invitrogen, #A21244), and Alexa488-conjugated anti-rabbit (Invitrogen #A11008).

Patch clamp

Apical turns of the organs of Corti from 2- to 3-week-old mice were isolated in ice-cold Hepes Hank's solution containing: 5.26 mM KCl, 141.7 mM NaCl, 0.5 mM $MgSO_4.7H_2O$, 10 mM Hepes, 1 mM $MgCl_2$, 11.1 mM D-glucose and 3.42 L-glutamine, pH adjusted to around 7.2 and osmolality of ~300 mOsm/kg. The recording chamber was perfused with modified Ringer's solution containing 2.8 mM KCl, 111 mM NaCl, 35 mM TEA-Cl (tetraethylammonium chloride), 10 mM Hepes, 1 mM CsCl, 1 mM $MgCl_2$, 11.1 mM D-glucose, and 1.3 or 3 mM $CaCl_2$, pH adjusted to around 7.2 and osmolality of ~300 mOsm/kg. The cleaning of the tissue was performed to make the IHCs accessible for patch clamp by removing the tectorial membrane and neighboring cells. The clean exposed basolateral surface of IHCs was patch-clamped in perforated patch configuration using an EPC-10 amplifier (HEKA Electronics, Germany) controlled by Patchmaster software at room temperature, as described previously (*57*). The pipette solution contained 137 mM Cs-gluconate, 10 mM TEA-Cl, 10 mM 4-aminopyridine, 10 mM Hepes, 1 mM $MgCl_2$, and amphotericin B (300 μg/ml), pH adjusted to 7.2 using HCl and osmolality of ~290 mOsm/kg. Cells were kept at a holding potential of −84 mV. All voltages were corrected for liquid junction potential (14 mV) offline. Currents were leak corrected using a p/10 protocol. Recordings were discarded when the leak current exceeded −50 pA, series resistance exceeded 30 megohm, or Ca^{2+}-current rundown exceeded 25%. Current-voltage relationships (*I-V*s) were recorded once the access resistance dropped below 30 megohm by applying increasing 10-ms-long step depolarization pulses of voltage ranging from −85 to 65 mV, in steps of 5 mV. Exocytosis measurements were performed by measuring increments in membrane capacitance (ΔC_m) using the Lindau-Neher technique (*98*). ΔC_m was recorded by stimulating the cells at the potential for maximal Ca^{2+} influx (−14 mV) for variable durations. Successive stimuli were acquired at an interval of 10 to 90 s. Each protocol was sequentially applied two to three times, and only IHCs with reproducible measurements were included. For analysis, capacitance traces were averaged over 400 ms before and after the depolarization (skipping the first 60 ms after the end of depolarization or first 5, 10, and 25 ms in the case of dual pulse experiments with interpulse intervals of 25, 50, and 100 ms, respectively). The traces were subjected to 5 or 10 pass binomial smoothing using Igor Pro 6 (WaveMetrics Inc., Lake Oswego, USA) for display. For the Zn^{2+} perfusion experiments, 1 mM Zn^{2+} was slowly perfused in and out of the recording chamber, while IHCs were step depolarized for 20 ms every 20 s. Normalized ΔC_m versus Q_{Ca} of control were plotted and power function [norm. $\Delta C_m = A(\text{norm. } Q_{Ca})^m$] fits were done in Igor Pro using the Levenberg-Marquardt algorithm.

In vivo recordings

Recordings of ABRs and otoacoustic emissions

Mice at the age of 6 to 8 weeks were anesthetized by intraperitoneal injection of a combination of ketamine (125 mg/kg) and xylazine (2.5 mg/kg). In all in vivo experiments, the core temperature was constantly maintained at 37°C using a heat blanket (Hugo Sachs Elektronik–Harvard Apparatus) or a custom-designed heat plate. To record ABRs, signals between subcutaneous needle electrodes at the vertex and mastoid were amplified 10,000 times and averaged at least 2 × 1300 times for each stimulus type in 10-dB steps using TDT System III and a custom MATLAB software (MathWorks) with a software filter between 300 and 3000 Hz. ABR thresholds were subjectively estimated by two independent observers as the lowest intensity yielding a reproducible ABR waveform. Amplitudes were determined from peak to trough.

Distortion product otocoustic emissions (DPOAEs) were recorded using Tucker Davis Technologies (TDT) System III and custom MATLAB software, TDT MF1 speakers, a Sennheiser MKE-2 microphone, and a UAC zoom-2 microphone preamplifier. All stimuli were calibrated using a quarter-inch Bruel and Kjaer D 4039 microphone.

Juxtacellular recordings from single SGNs

Mice (12 to 18 weeks, average age: 14 weeks) were anesthetized by inhalation of isoflurane via a face mask (5 vol % in air for induction and 0.5 to 3.0 vol % in air for maintenance). Analgesia was achieved using subcutaneous injections of buprenorphine (0.1 mg/kg, repeated every 4 hours) and carprofen (5 mg/kg, administered only once at the beginning of the experiment). The animals were maintained at 37°C throughout the experiment using a custom-made heating pad with a rectal temperature probe and placed on a vibration isolation table in a sound-proof chamber (IAC GmbH, Niederkrüchten, Germany). The depth of the anesthesia was regularly monitored by the absence of hind limb withdrawal reflexes, and anesthesia levels were adjusted as needed. For the juxtacellular recordings from the auditory nerve, a tracheostomy was performed using a silicon tube to support breathing throughout the experiment and later stereotactic head fixation. To ensure constant flow of isoflurane, the face mask delivering the anesthetic was held in close proximity to the face and tracheal opening of the animals throughout this procedure until the silicone tube was inserted and secured in the tracheal opening. The animals were then rapidly positioned in a custom-designed stereotactic head holder, and a 3D printed adaptor was attached to the face mask to efficiently deliver the isoflurane directly to the tracheostomy tube until the end of the experiment. After positioning the animal in the stereotactic head holder, the pinnae were removed, the scalp was reflected, and part of the left occipital bone was removed. This procedure then allowed for a partial aspiration of the cerebellum to expose the anterior semicircular canal as a landmark for electrode positioning.

The procedure of juxtacellular recordings from SGNs has been described previously (*99*). Glass microelectrodes (~50 megohm, backfilled with 3 M NaCl and containing a silver chloride recording electrode) were advanced step-wise through the posterior end of the anteroventral cochlear nucleus using an LN Mini 55 micro-manipulator (Luigs & Neumann, Germany), aiming toward the internal auditory canal. Acoustic stimulation was provided by an open field Avisoft ScanSpeak Ultrasonic Speaker (Avisoft Bioacoustics, Germany). Noise bursts (50 ms) served as search stimuli. The spiking responses of isolated sound-responsive neurons were detected and recorded using TDT system III hardware and amplified using ELC-03XS amplifier (NPI Electronic, Tamm, Germany), filtered using a bandpass filter (300 to 3000 Hz). Offline analysis was performed using visually established thresholds and waveform-based spike detection by a custom-written MATLAB script. Responses from putative SGN

axons were distinguished from cochlear nucleus neurons based on their stereotactic position, peristimulus time histogram, regularity of firing, first spike latency, and spike waveform (*99*, *100*).

Electron microscopy

Conventional embedding was performed as described before (*101*, *102*). Cochleae from two *Otof*$^{TDA/TDA}$ mice (*34*) and one *Otof*$^{+/+}$ mouse at postnatal day (P)15 were dissected as described above. Subsequently, the organs of Corti were fixed immediately after dissection with 4% paraformaldehyde (0335.1, Carl Roth, Germany) and 0.5% glutaraldehyde (G7651, Sigma-Aldrich, Germany) in PBS (P4417, Sigma-Aldrich, Germany; pH 7.4) for 1 hour on ice, followed by a second fixation step overnight with 2% glutaraldehyde in 0.1 M sodium cacodylate buffer (v/v, pH 7.2) on ice. Next, specimens were washed in 0.1 M sodium cacodylate buffer and treated with 1% osmium tetroxide (75632, Sigma-Aldrich, Germany; v/v in 0.1 M sodium cacodylate buffer) for 1 hour on ice, followed by further sodium cacodylate buffer and distilled water washing steps. After the en bloc staining with 1% uranyl acetate (8473, Merck, Germany; v/v in distilled water) for 1 hour on ice, samples were briefly washed in distilled water, dehydrated in an ascending concentration series of ethanol (30, 50, 70, 95, and 100%), infiltrated, and embedded in epoxy resin (R1140, AGAR-100, Plano, Germany) for at least 48 hours at 70°C for final polymerization.

For electron tomography, semithin sections (250 nm) from the conventionally embedded samples were applied to formvar-coated 100 copper mesh grids and poststained with UranyLess solution (DM22409, EMS, Science Services GmbH); subsequently, gold particles (10 nm) were applied to both sides of the grid. Electron tomograms were acquired at 200 kV as single-axis tilts at a JEOL JEM 2100Plus equipped with a 20 MP XAROSA bottom-mount CMOS TEM camera (EMSIS) from −58° to +58° using the Serial-EM software package (*103*) and subsequently generated using Etomo from the IMOD software package (*104*).

Analysis of electron tomography data

Synaptic vesicles, synaptic ribbons, presynaptic densities, and AZ membranes were automatically segmented with SynapseNet (*105*). The artificial neural network for synaptic vesicle segmentation was adapted on six tomograms with unsupervised domain adaptation; the network for synaptic ribbon, presynaptic density, and AZ membrane segmentation were used as is. Of the 18 acquired tomograms, 13 were chosen for analysis; 5 tomograms with low contrast were excluded. The segmentation results were manually corrected in napari (*106*). The distances between synaptic vesicles and the other structures were measured automatically, and the vesicles were divided into two pools: ribbon-associated synaptic vesicles (RA-SVs) that were within 80 nm of the synaptic ribbon and membrane-proximal synaptic vesicles (MP-SVs) that were within 100 nm of the presynaptic density and within 50 nm of the AZ membrane, as defined previously (*107*). For the MP-SVs the subpopulation of docked vesicles, which were in direct contact with the membrane, was identified by a distance to the membrane smaller than 2 nm. The pool assignment was manually corrected in napari to exclude vesicles in the second row, i.e., for which the ribbon was occluded by another vesicle, from the RA-SVs and to correct the assignment of docked vesicles, which was sensitive to small segmentation inaccuracies. Five tomograms contained two ribbon synapses; in three of these, both synapses were segmented, and in the other two, only one was segmented because the other was only partially contained in the tomogram. In the case of two segmented ribbon synapses, each vesicle was assigned to the closest ribbon. Based on pool assignments and distance measurements, the number of RA-SVs, MP-SVs, and the fraction of docked vesicles was plotted (fig. S9, D to F), and the distances of MP-SVs to AZ membrane and presynaptic density were analyzed (fig. S9, G and H).

Molecular dynamics simulations

All MD simulations were based on the cryo-EM model of the otoferlin structure. The missing disordered loops were manually built and corrected in Coot (v0.9.8.5) (*93*), guided by AlphaFold3 predictions and low-pass–filtered maps of the otoferlin (216 to 1931)–nanodisc complex. The resulting model was refined and further corrected in ISOLDE (*92*), with distance, position, and reference model restraints switched on. A symmetric lipid bilayer—consisting of 60% POPC, 30% POPS, 5% POPI24, and 5% POPI25—was created with CharmmGUI (*61*). The otoferlin molecule was positioned in the same orientation as observed in the cryo-EM structure so that its center of mass was 40 to 47 Å above the lipid headgroups. Subsequently, the system was solvated, and K^+ and Cl^- ions were added to neutralize the system and set a total KCl concentration of 0.15 M. For calcium ions bound to otoferlin, we used the multisite calcium model (*108*). We chose the CHARMM36M forcefield (*109*) to parameterize the system. The energy minimization, equilibration, and production simulation runs were performed in GROMACS version 2024.4 (*110*). We performed three minimization steps with the steepest descent algorithm: In the first one, position restraints were applied for protein backbone (4000 kJ/mol × nm^2), protein side chains (2000 kJ/mol × nm^2), dihedrals (1000 kJ/mol × nm^2), lipids (1000 kJ/mol × nm^2), and D0 pseudoatoms in the multisite calcium model (1000 kJ/mol × nm^2); in the second step, force constants of position restraints were decreased; last, in the last minimization run, no position restraints were applied. Minimization was followed by two equilibration steps. First, the system was simulated for 0.5 ns in the NVT (constant number of particles, volume, and temperature) ensemble with position restraints for protein backbone (1000 kJ/mol × nm^2), protein side chains (1 kJ/mol × nm^2), dihedrals (100 kJ/mol × nm^2), lipids (500 kJ/mol × nm^2), and D0 pseudoatoms in the multisite calcium model (1000 kJ/mol × nm^2). NVT equilibration was followed by a 2-ns simulation in the NPT (constant number of particles, pressure, and temperature) ensemble (no position restraints applied). In all simulations, the average temperature was kept at 300 K by the modified Berendsen thermostat with three coupling groups: membrane, protein bound to calcium ions, and solvent (*111*). For NPT equilibration and production simulations, the pressure was kept at 1 bar by semiisotropic exponential relaxation pressure coupling (*112*). The length of all bonds with hydrogens was constrained using the P-LINCS algorithm (*113*). The integration time step constituted 2 fs in all simulations. A 1.2-nm cutoff value was used for nonbonded forces. Van der Waals interactions were gradually switched off between 1 and 1.2 nm. To calculate long-ranged electrostatic interactions, the particle mesh Ewald method was used with 0.12-nm grid spacing (*114*). Five 2-μs-long simulation replicates were performed to study otoferlin binding to the model lipid bilayer.

Simulation analysis

Lipid contacts and root mean square deviation were analyzed using GROMACS (*110*). Contacts were defined as atoms being at 0.6 nm or less from each other. Calcium ions and hydrogens were not

included in contact analysis. Frames for analysis were taken with a step of 1 ns. For studying the electrostatic potential of domain surfaces, we used the APBS Electrostatics Plugin in Pymol (*115*) with temperature set at 300 K. The lipid densities at the interface of C_2 domains and the structural differences between simulations and the cryo-EM model were analyzed based on the last 200 ns of simulation replicates with help of the MDAnalysis Python library (*116*).

Quantification and statistical analysis

Data were analyzed using Wavemetrics Igor Pro, GraphPad Prism, or IBM SPSS Statistics. The normality of the data was assessed with the Kolmogorov-Smirnov test, and the equality of variances in normally distributed data was assessed with the *F* test or the Jarque-Bera test. Differences between two groups were evaluated for significant differences using the two-tailed unpaired Student's *t* test or, when not normally distributed and/or variance was unequal, the unpaired two-tailed Wilcoxon signed rank test. *P* values were corrected for multiple comparisons using the Bonferroni method. Significant differences are indicated as $*P < 0.05$, $**P < 0.01$, and $***P < 0.001$. The statistical details of individual experiments including the *n* values can be found in the figure legends.

Supplementary Materials

The PDF file includes:

Figs. S1 to S15
Tables S1 to S4
Legends for movies S1 to S4
References

Other Supplementary Material for this manuscript includes the following:

Movies S1 to S4

REFERENCES AND NOTES

1. T. Moser, C. P. Grabner, F. Schmitz, Sensory processing at ribbon synapses in the retina and the cochlea. *Physiol. Rev.* **100**, 103–144 (2020).
2. M. A. Rutherford, H. Von Gersdorff, J. D. Goutman, Encoding sound in the cochlea: From receptor potential to afferent discharge. *J. Physiol.* **599**, 2527–2557 (2021).
3. S. L. Johnson, S. Safieddine, M. Mustapha, W. Marcotti, Hair cell afferent synapses: Function and dysfunction. *Cold Spring Harb. Perspect. Med.* **9**, a033175 (2019).
4. S. Safieddine, R. J. Wenthold, SNARE complex at the ribbon synapses of cochlear hair cells: Analysis of synaptic vesicle- and synaptic membrane-associated proteins. *Eur. J. Neurosci.* **11**, 803–812 (1999).
5. A. Brandt, J. Striessnig, T. Moser, CaV1.3 channels are essential for development and presynaptic activity of cochlear inner hair cells. *J. Neurosci.* **23**, 10832–10840 (2003).
6. I. Roux, S. Safieddine, R. Nouvian, M. Grati, M.-C. Simmler, A. Bahloul, I. Perfettini, M. Le Gall, P. Rostaing, G. Hamard, A. Triller, P. Avan, T. Moser, C. Petit, Otoferlin, defective in a human deafness form, is essential for exocytosis at the auditory ribbon synapse. *Cell* **127**, 277–289 (2006).
7. R. P. Seal, O. Akil, E. Yi, C. M. Weber, L. Grant, J. Yoo, A. Clause, K. Kandler, J. L. Noebels, E. Glowatzki, L. R. Lustig, R. H. Edwards, Sensorineural deafness and seizures in mice lacking vesicular glutamate transporter 3. *Neuron* **57**, 263–275 (2008).
8. N. Strenzke, S. Chanda, C. Kopp-Scheinpflug, D. Khimich, K. Reim, A. V. Bulankina, A. Neef, F. Wolf, N. Brose, M. A. Xu-Friedman, T. Moser, Complexin-I is required for high-fidelity transmission at the endbulb of held auditory synapse. *J. Neurosci.* **29**, 7991–8004 (2009).
9. R. C. Uthaiah, A. J. Hudspeth, Molecular anatomy of the hair cell's ribbon synapse. *J. Neurosci.* **30**, 12387–12399 (2010).
10. E. Reisinger, C. Bresee, J. Neef, R. Nair, K. Reuter, A. Bulankina, R. Nouvian, M. Koch, J. Bückers, L. Kastrup, I. Roux, C. Petit, S. W. Hell, N. Brose, J.-S. Rhee, S. Kügler, J. V. Brigande, T. Moser, Probing the functional equivalence of otoferlin and synaptotagmin 1 in exocytosis. *J. Neurosci.* **31**, 4886–4895 (2011).
11. R. Nouvian, J. Neef, A. V. Bulankina, E. Reisinger, T. Pangršič, T. Frank, S. Sikorra, N. Brose, T. Binz, T. Moser, Exocytosis at the hair cell ribbon synapse apparently operates without neuronal SNARE proteins. *Nat. Neurosci.* **14**, 411–413 (2011).
12. C. Vogl, B. H. Cooper, J. Neef, S. M. Wojcik, K. Reim, E. Reisinger, N. Brose, J.-S. Rhee, T. Moser, C. Wichmann, Unconventional molecular regulation of synaptic vesicle replenishment in cochlear inner hair cells. *J. Cell Sci.* **128**, 638–644 (2015).
13. A. P. Cepeda, M. Ninov, J. Neef, I. Parfentev, K. Kusch, E. Reisinger, R. Jahn, T. Moser, H. Urlaub, Proteomic analysis reveals the composition of glutamatergic organelles of auditory inner hair cells. *Mol. Cell. Proteomics* **23**, 100704 (2024).
14. J. Rizo, Molecular mechanisms underlying neurotransmitter release. *Annu. Rev. Biophys.* **51**, 377–408 (2022).
15. R. Jahn, D. C. Cafiso, L. K. Tamm, Mechanisms of SNARE proteins in membrane fusion. *Nat. Rev. Mol. Cell Biol.* **25**, 101–118 (2024).
16. C. Calvet, T. Peineau, N. Benamer, M. Cornille, A. Lelli, B. Plion, G. Lahlou, J. Fanchette, S. Nouaille, J. B. de Monvel, A. Estivalet, P. Jean, V. Michel, M. Sachse, N. Michalski, P. Avan, C. Petit, D. Dulon, S. Safieddine, The SNARE protein SNAP-25 is required for normal exocytosis at auditory hair cell ribbon synapses. *iScience* **25**, 105628 (2022).
17. T. Moser, A. Starr, Auditory neuropathy—Neural and synaptic mechanisms. *Nat. Rev. Neurol.* **12**, 135–149 (2016).
18. S. Yasunaga, M. Grati, M. Cohen-Salmon, A. El-Amraoui, M. Mustapha, N. Salem, E. El-Zir, J. Loiselet, C. Petit, A mutation in OTOF, encoding otoferlin, a FER-1-like protein, causes DFNB9, a nonsyndromic form of deafness. *Nat. Genet.* **21**, 363–369 (1999).
19. S. M. Baig, A. Koschak, A. Lieb, M. Gebhart, C. Dafinger, G. Nürnberg, A. Ali, I. Ahmad, M. J. Sinnegger-Brauns, N. Brandt, J. Engel, M. E. Mangoni, M. Farooq, H. U. Khan, P. Nürnberg, J. Striessnig, H. J. Bolz, Loss of Ca(v)1.3 (CACNA1D) function in a human channelopathy with bradycardia and congenital deafness. *Nat. Neurosci.* **14**, 77–84 (2011).
20. I. Schrauwen, S. Helfmann, A. Inagaki, F. Predoehl, M. A. Tabatabaiefar, M. M. Picher, M. Sommen, C. Z. Seco, J. Oostrik, H. Kremer, A. Dheedene, C. Claes, E. Fransen, M. H. Chaleshtori, P. Coucke, A. Lee, T. Moser, G. Van Camp, A mutation in CABP2, expressed in cochlear hair cells, causes autosomal-recessive hearing impairment. *Am. J. Hum. Genet.* **91**, 636–645 (2012).
21. J. Platzer, J. Engel, A. Schrott-Fischer, K. Stephan, S. Bova, H. Chen, H. Zheng, J. Striessnig, Congenital deafness and sinoatrial node dysfunction in mice lacking class D L-type Ca^{2+} channels. *Cell* **102**, 89–97 (2000).
22. A. Brandt, D. Khimich, T. Moser, Few CaV1.3 channels regulate the exocytosis of a synaptic vesicle at the hair cell ribbon synapse. *J. Neurosci.* **25**, 11577–11585 (2005).
23. T. Pangršič, M. Gabrielaitis, S. Michanski, B. Schwaller, F. Wolf, N. Strenzke, T. Moser, EF-hand protein Ca^{2+} buffers regulate Ca^{2+} influx and exocytosis in sensory hair cells. *Proc. Natl. Acad. Sci. U.S.A.* **112**, E1028–E1037 (2015).
24. L. M. Jaime Tobón, T. Moser, Ca^{2+} regulation of glutamate release from inner hair cells of hearing mice. *Proc. Natl. Acad. Sci. U.S.A.* **120**, e2311539120 (2023).
25. T. Pangrsic, E. Reisinger, T. Moser, Otoferlin: A multi-C2 domain protein essential for hearing. *Trends Neurosci.* **35**, 671–680 (2012).
26. S. T. Cooper, P. L. McNeil, Membrane repair: Mechanisms and pathophysiology. *Physiol. Rev.* **95**, 1205–1240 (2015).
27. T. Pangrsic, L. Lasarow, K. Reuter, H. Takago, M. Schwander, D. Riedel, T. Frank, L. M. Tarantino, J. S. Bailey, N. Strenzke, N. Brose, U. Müller, E. Reisinger, T. Moser, Hearing requires otoferlin-dependent efficient replenishment of synaptic vesicles in hair cells. *Nat. Neurosci.* **13**, 869–876 (2010).
28. N. H. Revelo, D. Kamin, S. Truckenbrodt, A. B. Wong, K. Reuter-Jessen, E. Reisinger, T. Moser, S. O. Rizzoli, A new probe for super-resolution imaging of membranes elucidates trafficking pathways. *J. Cell Biol.* **205**, 591–606 (2014).
29. S. Jung, T. Maritzen, C. Wichmann, Z. Jing, A. Neef, N. H. Revelo, H. Al-Moyed, S. Meese, S. M. Wojcik, I. Panou, H. Bulut, P. Schu, R. Ficner, E. Reisinger, S. O. Rizzoli, J. Neef, N. Strenzke, V. Haucke, T. Moser, Disruption of adaptor protein 2μ (AP-2μ) in cochlear hair cells impairs vesicle reloading of synaptic release sites and hearing. *EMBO J.* **34**, 2686–2702 (2015).
30. C. P. Johnson, E. R. Chapman, Otoferlin is a calcium sensor that directly regulates SNARE-mediated membrane fusion. *J. Cell Biol.* **191**, 187–197 (2010).
31. N. Michalski, J. D. Goutman, S. M. Auclair, J. Boutet de Monvel, M. Tertrais, A. Emptoz, A. Parrin, S. Nouaille, M. Guillon, M. Sachse, D. Ciric, A. Bahloul, J.-P. Hardelin, R. B. Sutton, P. Avan, S. S. Krishnakumar, J. E. Rothman, D. Dulon, S. Safieddine, C. Petit, Otoferlin acts as a Ca^{2+} sensor for vesicle fusion and vesicle pool replenishment at auditory hair cell ribbon synapses. *eLife* **6**, e31013 (2017).
32. R. Fernández-Chacón, A. Königstorfer, S. H. Gerber, J. García, M. F. Matos, C. F. Stevens, N. Brose, J. Rizo, C. Rosenmund, T. C. Südhof, Synaptotagmin I functions as a calcium regulator of release probability. *Nature* **410**, 41–49 (2001).
33. O. Kochubey, R. Schneggenburger, Synaptotagmin increases the dynamic range of synapses by driving Ca^{2+}–evoked release and by clamping a near-linear remaining Ca^{2+} sensor. *Neuron* **69**, 736–748 (2011).
34. H. Chen, M. Monga, Q. Fang, L. Slitin, J. Neef, S. S. Chepurwar, R. C. M. Netto, K. Lezirovitz, A. Tabith, F. Benseler, N. Brose, K. Kusch, C. Wichmann, N. Strenzke, B. Vona, J. Preobraschenski, T. Moser, Ca^{2+} binding to the C_2E domain of otoferlin is required for hair cell exocytosis and hearing. *Protein Cell* **15**, 305–312 (2024).
35. D. Beutner, T. Voets, E. Neher, T. Moser, Calcium dependence of exocytosis and endocytosis at the cochlear inner hair cell afferent synapse. *Neuron* **29**, 681–690 (2001).
36. C. Vogl, I. Panou, G. Yamanbaeva, C. Wichmann, S. J. Mangosing, F. Vilardi, A. A. Indzhykulian, T. Pangršič, R. Santarelli, M. Rodriguez-Ballesteros, T. Weber, S. Jung,

E. Cardenas, X. Wu, S. M. Wojcik, K. Y. Kwan, I. Del Castillo, B. Schwappach, N. Strenzke, D. P. Corey, S.-Y. Lin, T. Moser, Tryptophan-rich basic protein (WRB) mediates insertion of the tail-anchored protein otoferlin and is required for hair cell exocytosis and hearing. *EMBO J.* **35**, 2536–2552 (2016).

37. N. Strenzke, R. Chakrabarti, H. Al-Moyed, A. Müller, G. Hoch, T. Pangrsic, G. Yamanbaeva, C. Lenz, K.-T. Pan, E. Auge, R. Geiss-Friedlander, H. Urlaub, N. Brose, C. Wichmann, E. Reisinger, Hair cell synaptic dysfunction, auditory fatigue and thermal sensitivity in otoferlin Ile515Thr mutants. *EMBO J.* **35**, 2519–2535 (2016).
38. S. V. Duncker, C. Franz, S. Kuhn, U. Schulte, D. Campanelli, N. Brandt, B. Hirt, B. Fakler, N. Blin, P. Ruth, J. Engel, W. Marcotti, U. Zimmermann, M. Knipper, Otoferlin couples to clathrin-mediated endocytosis in mature cochlear inner hair cells. *J. Neurosci.* **33**, 9508–9519 (2013).
39. M. Tertrais, Y. Bouleau, A. Emptoz, S. Belleudy, R. B. Sutton, C. Petit, S. Safieddine, D. Dulon, Viral transfer of mini-otoferlins partially restores the fast component of exocytosis and uncovers ultrafast endocytosis in auditory hair cells of otoferlin knock-out mice. *J. Neurosci.* **39**, 3394–3411 (2019).
40. N. Hams, M. Padmanarayana, W. Qiu, C. P. Johnson, Otoferlin is a multivalent calcium-sensitive scaffold linking SNAREs and calcium channels. *Proc. Natl. Acad. Sci. U.S.A.* **114**, 8023–8028 (2017).
41. S. Helfmann, P. Neumann, K. Tittmann, T. Moser, R. Ficner, E. Reisinger, The crystal structure of the C_2A domain of otoferlin reveals an unconventional top loop region. *J. Mol. Biol.* **406**, 479–490 (2011).
42. A. H. Shaib, A. A. Chouaib, R. Chowdhury, J. Altendorf, D. Mihaylov, C. Zhang, D. Krah, V. Imani, R. K. W. Spencer, S. V. Georgiev, N. Mougios, M. Monga, S. Reshetniak, T. Mimoso, H. Chen, P. Fatehbasharzad, D. Crzan, K.-A. Saal, M. M. Alawieh, N. Alawar, J. Eilts, J. Kang, A. Soleimani, M. Müller, C. Pape, L. Alvarez, C. Trenkwalder, B. Mollenhauer, T. F. Outeiro, S. Köster, J. Preobraschenski, U. Becherer, T. Moser, E. S. Boyden, A. R. Aricescu, M. Sauer, F. Opazo, S. O. Rizzoli, One-step nanoscale expansion microscopy reveals individual protein shapes. *Nat. Biotechnol.* **43**, 1539–1547 (2025).
43. H.-L. Huang, G. Grandinetti, S. M. Heissler, K. Chinthalapudi, Cryo-EM structures of the membrane repair protein dysferlin. *Nat. Commun.* **15**, 9650 (2024).
44. C. Cretu, A. Chernev, C. Z. Kibédi Szabó, V. Pena, H. Urlaub, T. Moser, J. Preobraschenski, Structural insights into lipid membrane binding by human ferlins. *EMBO J.* **44**, 3926–3958 (2025).
45. B. Vona, A. Rad, E. Reisinger, The many faces of DFNB9: Relating OTOF variants to hearing impairment. *Genes* **11**, 1411 (2020).
46. H. Al-Moyed, A. P. Cepeda, S. Jung, T. Moser, S. Kügler, E. Reisinger, A dual-AAV approach restores fast exocytosis and partially rescues auditory function in deaf otoferlin knock-out mice. *EMBO Mol. Med.* **11**, e9396 (2019).
47. O. Akil, F. Dyka, C. Calvet, A. Emptoz, G. Lahlou, S. Nouaille, J. B. de Monvel, J.-P. Hardelin, W. W. Hauswirth, P. Avan, C. Petit, S. Safieddine, L. R. Lustig, Dual AAV-mediated gene therapy restores hearing in a DFNB9 mouse model. *Proc. Natl. Acad. Sci. U.S.A.* **116**, 4496–4501 (2019).
48. J. Lv, H. Wang, X. Cheng, Y. Chen, D. Wang, L. Zhang, Q. Cao, H. Tang, S. Hu, K. Gao, M. Xun, J. Wang, Z. Wang, B. Zhu, C. Cui, Z. Gao, L. Guo, S. Yu, L. Jiang, Y. Yin, J. Zhang, B. Chen, W. Wang, R. Chai, Z.-Y. Chen, H. Li, Y. Shu, AAV1-hOTOF gene therapy for autosomal recessive deafness 9: A single-arm trial. *Lancet* **403**, 2317–2325 (2024).
49. J. Qi, F. Tan, L. Zhang, L. Lu, S. Zhang, Y. Zhai, Y. Lu, X. Qian, W. Dong, Y. Zhou, Z. Zhang, X. Yang, L. Jiang, C. Yu, J. Liu, T. Chen, L. Wu, C. Tan, S. Sun, H. Song, Y. Shu, L. Xu, X. Gao, H. Li, R. Chai, AAV-mediated gene therapy restores hearing in patients with DFNB9 deafness. *Adv. Sci.* **11**, e2306788 (2024).
50. H. Wang, Y. Chen, J. Lv, X. Cheng, Q. Cao, D. Wang, L. Zhang, B. Zhu, M. Shen, C. Xu, M. Xun, Z. Wang, H. Tang, S. Hu, C. Cui, L. Jiang, Y. Yin, L. Guo, Y. Zhou, L. Han, Z. Gao, J. Zhang, S. Yu, K. Gao, J. Wang, B. Chen, W. Wang, Z.-Y. Chen, H. Li, Y. Shu, Bilateral gene therapy in children with autosomal recessive deafness 9: Single-arm trial results. *Nat. Med.* **30**, 1898–1904 (2024).
51. H. Liu, H. Liu, L. Wang, L. Song, G. Jiang, Q. Lu, T. Yang, H. Peng, R. Cai, X. Zhao, T. Zhao, H. Wu, Cochlear transcript diversity and its role in auditory functions implied by an otoferlin short isoform. *Nat. Commun.* **14**, 3085 (2023).
52. K. S. Cannon, R. D. Sarsam, T. Tedamrongwanish, K. Zhang, R. W. Baker, Lipid nanodiscs as a template for high-resolution cryo-EM structures of peripheral membrane proteins. *J. Struct. Biol.* **215**, 107989 (2023).
53. H. Chen, Q. Fang, F. Benseler, N. Brose, T. Moser, Probing the role of the C_2F domain of otoferlin. *Front. Mol. Neurosci.* **16**, 1299509 (2023).
54. M. Padmanarayana, N. Hams, L. C. Speight, E. J. Petersson, R. A. Mehl, C. P. Johnson, Characterization of the lipid binding properties of otoferlin reveals specific interactions between PI(4,5)P2 and the C2C and C2F domains. *Biochemistry* **53**, 5023–5033 (2014).
55. N. J. Marty, C. L. Holman, N. Abdullah, C. P. Johnson, The C2 domains of otoferlin, dysferlin, and myoferlin alter the packing of lipid bilayers. *Biochemistry* **52**, 5585–5592 (2013).
56. E. Neher, N. Brose, Dynamically primed synaptic vesicle states: Key to understand synaptic short-term plasticity. *Neuron* **100**, 1283–1291 (2018).
57. T. Moser, D. Beutner, Kinetics of exocytosis and endocytosis at the cochlear inner hair cell afferent synapse of the mouse. *Proc. Natl. Acad. Sci. U.S.A.* **97**, 883–888 (2000).
58. J. D. Goutman, E. Glowatzki, Time course and calcium dependence of transmitter release at a single ribbon synapse. *Proc. Natl. Acad. Sci. U.S.A.* **104**, 16341–16346 (2007).
59. L. M. Jaime Tobón, T. Moser, Bridging the gap between presynaptic hair cell function and neural sound encoding. *eLife* **12**, RP93749 (2024).
60. D. Bansal, K. Miyake, S. S. Vogel, S. Groh, C.-C. Chen, R. Williamson, P. L. McNeil, K. P. Campbell, Defective membrane repair in dysferlin-deficient muscular dystrophy. *Nature* **423**, 168–172 (2003).
61. S. Jo, T. Kim, V. G. Iyer, W. Im, CHARMM-GUI: A web-based graphical user interface for CHARMM. *J. Comput. Chem.* **29**, 1859–1865 (2008).
62. J. Ruel, S. Emery, R. Nouvian, T. Bersot, B. Amilhon, J. M. Van Rybroek, G. Rebillard, M. Lenoir, M. Eybalin, B. Delprat, T. A. Sivakumaran, B. Giros, S. El Mestikawy, T. Moser, R. J. H. Smith, M. M. Lesperance, J.-L. Puel, Impairment of SLC17A8 encoding vesicular glutamate transporter-3, VGLUT3, underlies nonsyndromic deafness DFNA25 and inner hair cell dysfunction in null mice. *Am. J. Hum. Genet.* **83**, 278–292 (2008).
63. D. Khimich, R. Nouvian, R. Pujol, S. T. Dieck, A. Egner, E. D. Gundelfinger, T. Moser, Hair cell synaptic ribbons are essential for synchronous auditory signalling. *Nature* **434**, 889–894 (2005).
64. T. Moser, "Presynaptic physiology of cochlear inner hair cells," in *The Senses: A Comprehensive Reference* (Elsevier, 2020), pp. 441–467; https://linkinghub.elsevier.com/retrieve/pii/B9780128093245241850.
65. Y. Hua, X. Ding, H. Wang, F. Wang, Y. Lu, J. Neef, Y. Gao, T. Moser, H. Wu, Electron microscopic reconstruction of neural circuitry in the cochlea. *Cell Rep.* **34**, 108551 (2021).
66. P. Heil, H. Neubauer, D. R. F. Irvine, M. Brown, Spontaneous activity of auditory-nerve fibers: insights into stochastic processes at ribbon synapses. *J. Neurosci.* **27**, 8457–8474 (2007).
67. B. N. Buran, N. Strenzke, A. Neef, E. D. Gundelfinger, T. Moser, M. C. Liberman, Onset coding is degraded in auditory nerve fibers from mutant mice lacking synaptic ribbons. *J. Neurosci.* **30**, 7587–7597 (2010).
68. M. Avissar, J. H. Wittig, J. C. Saunders, T. D. Parsons, Refractoriness enhances temporal coding by auditory nerve fibers. *J. Neurosci.* **33**, 7681–7690 (2013).
69. T. Frank, M. A. Rutherford, N. Strenzke, A. Neef, T. Pangršič, D. Khimich, A. Fejtova, E. D. Gundelfinger, M. C. Liberman, B. Harke, K. E. Bryan, A. Lee, A. Egner, D. Riedel, T. Moser, Bassoon and the synaptic ribbon organize Ca^{2+} channels and vesicles to add release sites and promote refilling. *Neuron* **68**, 724–738 (2010).
70. A. Neef, D. Khimich, P. Pirih, D. Riedel, F. Wolf, T. Moser, Probing the mechanism of exocytosis at the hair cell ribbon synapse. *J. Neurosci.* **27**, 12933–12944 (2007).
71. C. P. Grabner, T. Moser, Individual synaptic vesicles mediate stimulated exocytosis from cochlear inner hair cells. *Proc. Natl. Acad. Sci. U.S.A.* **115**, 12811–12816 (2018).
72. B. D. Winegar, J. B. Lansman, Voltage-dependent block by zinc of single calcium channels in mouse myotubes. *J. Physiol.* **425**, 563–578 (1990).
73. M. Beurg, N. Michalski, S. Safieddine, Y. Bouleau, R. Schneggenburger, E. R. Chapman, C. Petit, D. Dulon, Control of exocytosis by synaptotagmins and otoferlin in auditory hair cells. *J. Neurosci.* **30**, 13281–13290 (2010).
74. Y. Lai, U. B. Choi, J. Leitz, H. J. Rhee, C. Lee, B. Altas, M. Zhao, R. A. Pfuetzner, A. L. Wang, N. Brose, J. S. Rhee, A. T. Brunger, Molecular mechanisms of synaptic vesicle priming by Munc13 and Munc18. *Neuron* **95**, 591–607.e10 (2017).
75. B. Quade, M. Camacho, X. Zhao, M. Orlando, T. Trimbuch, J. Xu, W. Li, D. Nicastro, C. Rosenmund, J. Rizo, Membrane bridging by Munc13–1 is crucial for neurotransmitter release. *eLife* **8**, e42806 (2019).
76. M. Verhage, A. S. Maia, J. J. Plomp, A. B. Brussaard, J. H. Heeroma, H. Vermeer, R. F. Toonen, R. E. Hammer, T. K. van den Berg, M. Missler, H. J. Geuze, T. C. Südhof, Synaptic assembly of the brain in the absence of neurotransmitter secretion. *Science* **287**, 864–869 (2000).
77. F. Varoqueaux, A. Sigler, J.-S. Rhee, N. Brose, C. Enk, K. Reim, C. Rosenmund, Total arrest of spontaneous and evoked synaptic transmission but normal synaptogenesis in the absence of Munc13-mediated vesicle priming. *Proc. Natl. Acad. Sci. U.S.A.* **99**, 9037–9042 (2002).
78. A. T. Brunger, J. Leitz, The core complex of the Ca^{2+}–triggered presynaptic fusion machinery. *J. Mol. Biol.* **435**, 167853 (2023).
79. Q. Zhou, P. Zhou, A. L. Wang, D. Wu, M. Zhao, T. C. Südhof, A. T. Brunger, The primed SNARE-complexin-synaptotagmin complex for neuronal exocytosis. *Nature* **548**, 420–425 (2017).
80. M. Schwander, A. Sczaniecka, N. Grillet, J. S. Bailey, M. Avenarius, H. Najmabadi, B. M. Steffy, G. C. Federe, E. A. Lagler, R. Banan, R. Hice, L. Grabowski-Boase, E. M. Keithley, A. F. Ryan, G. D. Housley, T. Wiltshire, R. J. H. Smith, L. M. Tarantino, U. Müller, A forward genetics screen in mice identifies recessive deafness traits and reveals that pejvakin is essential for outer hair cell function. *J. Neurosci.* **27**, 2163–2175 (2007).
81. R. Chakrabarti, S. Michanski, C. Wichmann, Vesicle sub-pool organization at inner hair cell ribbon synapses. *EMBO Rep.* **19**, e44937 (2018).
82. T. Matsunaga, H. Mutai, S. Kunishima, K. Namba, N. Morimoto, Y. Shinjo, Y. Arimoto, Y. Kataoka, T. Shintani, N. Morita, T. Sugiuchi, S. Masuda, A. Nakano, H. Taiji, K. Kaga, A

prevalent founder mutation and genotype-phenotype correlations of OTOF in Japanese patients with auditory neuropathy. *Clin. Genet.* **82**, 425–432 (2012).

83. N. Karagulyan, A. Thirumalai, S. Michanski, Y. Qi, Q. Fang, H. Wang, N. J. Ortner, J. Striessnig, N. Strenzke, C. Wichmann, Y. Hua, T. Moser, Gating of hair cell Ca^{2+} channels governs the activity of cochlear neurons. *Sci. Adv.* **11**, eadu7898 (2025).
84. J. Schmitzová, C. Cretu, C. Dienemann, H. Urlaub, V. Pena, Structural basis of catalytic activation in human splicing. *Nature* **617**, 842–850 (2023).
85. C. Cretu, A. A. Agrawal, A. Cook, C. L. Will, P. Fekkes, P. G. Smith, R. Lührmann, N. Larsen, S. Buonamici, V. Pena, Structural basis of splicing modulation by antitumor macrolide compounds. *Mol. Cell* **70**, 265–273.e8 (2018).
86. C. Cretu, P. Gee, X. Liu, A. Agrawal, T.-V. Nguyen, A. K. Ghosh, A. Cook, M. Jurica, N. A. Larsen, V. Pena, Structural basis of intron selection by U2 snRNP in the presence of covalent inhibitors. *Nat. Commun.* **12**, 4491 (2021).
87. C. Cretu, J. Schmitzová, A. Ponce-Salvatierra, O. Dybkov, E. I. De Laurentiis, K. Sharma, C. L. Will, H. Urlaub, R. Lührmann, V. Pena, Molecular architecture of SF3b and structural consequences of its cancer-related mutations. *Mol. Cell* **64**, 307–319 (2016).
88. D. S. Brandt, M. D. Coffman, J. J. Falke, J. D. Knight, Hydrophobic contributions to the membrane docking of Synaptotagmin 7 C2A domain: Mechanistic contrast between isoforms 1 and 7. *Biochemistry* **51**, 7654–7664 (2012).
89. D. Kimanius, K. Jamali, M. E. Wilkinson, S. Lövestam, V. Velazhahan, T. Nakane, S. H. W. Scheres, Data-driven regularization lowers the size barrier of cryo-EM structure determination. *Nat Methods* **21**, 1216–1221 (2024).
90. A. J. Jakobi, M. Wilmanns, C. Sachse, Model-based local density sharpening of cryo-EM maps. *eLife* **6**, e27131 (2017).
91. J. Jumper, R. Evans, A. Pritzel, T. Green, M. Figurnov, O. Ronneberger, K. Tunyasuvunakool, R. Bates, A. Žídek, A. Potapenko, A. Bridgland, C. Meyer, S. A. A. Kohl, A. J. Ballard, A. Cowie, B. Romera-Paredes, S. Nikolov, R. Jain, J. Adler, T. Back, S. Petersen, D. Reiman, E. Clancy, M. Zielinski, M. Steinegger, M. Pacholska, T. Berghammer, S. Bodenstein, D. Silver, O. Vinyals, A. W. Senior, K. Kavukcuoglu, P. Kohli, D. Hassabis, Highly accurate protein structure prediction with AlphaFold. *Nature* **596**, 583–589 (2021).
92. T. I. Croll, ISOLDE: A physically realistic environment for model building into low-resolution electron-density maps. *Acta Crystallogr. D Struct. Biol.* **74**, 519–530 (2018).
93. P. Emsley, K. Cowtan, Coot: Model-building tools for molecular graphics. *Acta Crystallogr. D Biol. Crystallogr.* **60**, 2126–2132 (2004).
94. P. V. Afonine, B. K. Poon, R. J. Read, O. V. Sobolev, T. C. Terwilliger, A. Urzhumtsev, P. D. Adams, Real-space refinement in PHENIX for cryo-EM and crystallography. *Acta Crystallogr. D Struct. Biol.* **74**, 531–544 (2018).
95. R. D. Oeffner, T. I. Croll, C. Millán, B. K. Poon, C. J. Schlicksup, R. J. Read, T. C. Terwilliger, Putting AlphaFold models to work with phenix.process_predicted_model and ISOLDE. *Acta Crystallogr. D Struct. Biol.* **78**, 1303–1314 (2022).
96. K. Yamashita, C. M. Palmer, T. Burnley, G. N. Murshudov, Cryo-EM single-particle structure refinement and map calculation using Servalcat. *Acta Crystallogr. D Struct. Biol.* **77**, 1282–1291 (2021).
97. E. Krissinel, K. Henrick, Inference of macromolecular assemblies from crystalline state. *J. Mol. Biol.* **372**, 774–797 (2007).
98. M. Lindau, E. Neher, Patch-clamp techniques for time-resolved capacitance measurements in single cells. *Pflügers Arch.* **411**, 137–146 (1988).
99. Z. Jing, M. A. Rutherford, H. Takago, T. Frank, A. Fejtova, D. Khimich, T. Moser, N. Strenzke, Disruption of the presynaptic cytomatrix protein bassoon degrades ribbon anchorage, multiquantal release, and sound encoding at the hair cell afferent synapse. *J. Neurosci.* **33**, 4456–4467 (2013).
100. A. M. Taberner, M. C. Liberman, Response properties of single auditory nerve fibers in the mouse. *J. Neurophysiol.* **93**, 557–569 (2005).
101. A. B. Wong, M. A. Rutherford, M. Gabrielaitis, T. Pangršič, F. Göttfert, T. Frank, S. Michanski, S. Hell, F. Wolf, C. Wichmann, T. Moser, Developmental refinement of hair cell synapses tightens the coupling of Ca^{2+} influx to exocytosis. *EMBO J.* **33**, 247–264 (2014).
102. P. Jean, D. L. de la Morena, S. Michanski, L. M. J. Tobón, R. Chakrabarti, M. M. Picher, J. Neef, S. Jung, M. Gültas, S. Maxeiner, A. Neef, C. Wichmann, N. Strenzke, C. Grabner, T. Moser, The synaptic ribbon is critical for sound encoding at high rates and with temporal precision. *eLife* **7**, e29275 (2018).
103. D. N. Mastronarde, Automated electron microscope tomography using robust prediction of specimen movements. *J. Struct. Biol.* **152**, 36–51 (2005).
104. J. R. Kremer, D. N. Mastronarde, J. R. McIntosh, Computer visualization of three-dimensional image data using IMOD. *J. Struct. Biol.* **116**, 71–76 (1996).
105. S. Muth, F. Moschref, L. Freckmann, S. Mutschall, I. Hojas-Garcia-Plaza, J. N. Bahr, A. Petrovic, T. T. Do, V. Schwarze, A. Archit, K. Weyand, S. Michanski, L. Maus, C. Imig, N. Brose, C. Wichmann, R. Fernandez-Busnadiego, T. Moser, S. O. Rizzoli, B. H. Cooper, C. Pape, SynapseNet: Deep learning for automatic synapse reconstruction. *Mol. Biol. Cell*, mbcE24110519 (2025).
106. N. Sofroniew, T. Lambert, G. Bokota, J. Nunez-Iglesias, P. Sobolewski, A. Sweet, L. Gaifas, K. Evans, A. Burt, D. Doncila Pop, K. Yamauchi, M. Weber Mendonça, G. Buckley, W.-M. Vierdag, L. Royer, A. Can Solak, K. I. S. Harrington, J. Ahlers, D. Althviz Moré, O. Amsalem, A. Anderson, A. Annex, P. Boone, J. Bragantini, M. Bussonnier, C. Caporal, J. Eglinger, A. Eisenbarth, J. Freeman, C. Gohlke, K. Gunalan, H. Har-Gil, M. Harfouche, V. Hilsenstein, K. Hutchings, J. Lauer, G. Lichtner, Z. Liu, L. Liu, A. Lowe, L. Marconato, S. Martin, A. McGovern, L. Migas, N. Miller, H. Muñoz, J.-H. Müller, C. Nauroth-Kreß, D. Palecek, C. Pape, E. Perlman, K. Pevey, G. Peña-Castellanos, A. Pierré, D. Pinto, J. Rodríguez-Guerra, D. Ross, C. T. Russell, J. Ryan, G. Selzer, M. Smith, P. Smith, K. Sofiiuk, J. Soltwedel, D. Stansby, J. Vanaret, P. Wadhwa, M. Weigert, J. Windhager, P. Winston, R. Zhao, napari: A multi-dimensional image viewer for Python, version v0.5.5 (Zenodo, 2024); https://doi.org/10.5281/zenodo.14427406.
107. S. Krinner, T. Butola, S. Jung, C. Wichmann, T. Moser, RIM-binding protein 2 promotes a large number of CaV1.3 Ca^{2+}-channels and contributes to fast synaptic vesicle replenishment at hair cell active zones. *Front. Cell Neurosci.* **11**, 334 (2017).
108. A. Zhang, H. Yu, C. Liu, C. Song, The Ca^{2+} permeation mechanism of the ryanodine receptor revealed by a multi-site ion model. *Nat. Commun.* **11**, 922 (2020).
109. J. Huang, S. Rauscher, G. Nawrocki, T. Ran, M. Feig, B. L. de Groot, H. Grubmüller, A. D. MacKerell, CHARMM36m: An improved force field for folded and intrinsically disordered proteins. *Nat. Methods* **14**, 71–73 (2017).
110. M. J. Abraham, T. Murtola, R. Schulz, S. Páll, J. C. Smith, B. Hess, E. Lindahl, GROMACS: High performance molecular simulations through multi-level parallelism from laptops to supercomputers. *SoftwareX* **1–2**, 19–25 (2015).
111. G. Bussi, D. Donadio, M. Parrinello, Canonical sampling through velocity rescaling. *J. Chem. Phys.* **126**, 014101 (2007).
112. M. Bernetti, G. Bussi, Pressure control using stochastic cell rescaling. *J. Chem. Phys.* **153**, 114107 (2020).
113. B. Hess, P-LINCS: A parallel linear constraint solver for molecular simulation. *J. Chem. Theory Comput.* **4**, 116–122 (2008).
114. U. Essmann, L. Perera, M. L. Berkowitz, T. Darden, H. Lee, L. G. Pedersen, A smooth particle mesh Ewald method. *J. Chem. Phys.* **103**, 8577–8593 (1995).
115. E. Jurrus, D. Engel, K. Star, K. Monson, J. Brandi, L. E. Felberg, D. H. Brookes, L. Wilson, J. Chen, K. Liles, M. Chun, P. Li, D. W. Gohara, T. Dolinsky, R. Konecny, D. R. Koes, J. E. Nielsen, T. Head-Gordon, W. Geng, R. Krasny, G.-W. Wei, M. J. Holst, J. A. McCammon, N. A. Baker, Improvements to the APBS biomolecular solvation software suite. *Protein Sci.* **27**, 112–128 (2018).
116. F. B. Naughton, I. Alibay, J. Barnoud, E. Barreto-Ojeda, O. Beckstein, C. Bouysset, O. Cohen, R. J. Gowers, H. MacDermott-Opeskin, M. Matta, M. N. Melo, T. Reddy, L. Wang, Y. Zhuang, MDAnalysis 2.0 and beyond: Fast and interoperable, community driven simulation analysis. *Biophys. J.* **121**, 272a–273a (2022).
117. Y.-I. Iwasa, S.-Y. Nishio, A. Sugaya, Y. Kataoka, Y. Kanda, M. Taniguchi, K. Nagai, Y. Naito, T. Ikezono, R. Horie, Y. Sakurai, R. Matsuoka, H. Takeda, S. Abe, C. Kihara, T. Ishino, S.-Y. Morita, S. Iwasaki, M. Takahashi, T. Ito, Y. Arai, S.-I. Usami, OTOF mutation analysis with massively parallel DNA sequencing in 2,265 Japanese sensorineural hearing loss patients. *PLOS ONE* **14**, e0215932 (2019).
118. F. Mirghomizadeh, M. Pfister, F. Apaydin, C. Petit, S. Kupka, C. M. Pusch, H. P. Zenner, N. Blin, Substitutions in the conserved C2C domain of otoferlin cause DFNB9, a form of nonsyndromic autosomal recessive deafness. *Neurobiol. Dis.* **10**, 157–164 (2002).
119. Y. Wang, Y. Lu, J. Cheng, L. Zhang, D. Han, H. Yuan, Novel OTOF gene mutations identified using a massively parallel DNA sequencing technique in DFNB9 deafness. *Acta Otolaryngol.* **138**, 865–870 (2018).
120. J. Romanos, L. Kimura, M. L. Fávero, F. A. R. Izarra, M. T. B. de Mello Auricchio, A. C. Batissoco, K. Lezirovitz, R. S. Abreu-Silva, R. C. Mingroni-Netto, Novel OTOF mutations in Brazilian patients with auditory neuropathy. *J. Hum. Genet.* **54**, 382–385 (2009).
121. M. Yildirim-Baylan, G. Bademci, D. Duman, H. Ozturkmen-Akay, S. Tokgoz-Yilmaz, M. Tekin, Evidence for genotype-phenotype correlation for OTOF mutations. *Int. J. Pediatr. Otorhinolaryngol.* **78**, 950–953 (2014).
122. C. L. Ford, W. J. Riggs, T. Quigley, O. P. Keifer, J. P. Whitton, V. Valayannopoulos, The natural history, clinical outcomes, and genotype–phenotype relationship of otoferlin-related hearing loss: A systematic, quantitative literature review. *Hum. Genet.* **142**, 1429–1449 (2023).
123. Y.-I. Iwasa, S.-Y. Nishio, H. Yoshimura, A. Sugaya, Y. Kataoka, Y. Maeda, Y. Kanda, K. Nagai, Y. Naito, H. Yamazaki, T. Ikezono, H. Matsuda, M. Nakai, R. Tona, Y. Sakurai, R. Motegi, H. Takeda, M. Kobayashi, C. Kihara, T. Ishino, S.-Y. Morita, S. Iwasaki, M. Takahashi, S. Furutate, S.-I. Oka, T. Kubota, Y. Arai, Y. Kobayashi, D. Kikuchi, T. Shintani, N. Ogasawara, Y. Honkura, S. Izumi, M. Hyogo, Y. Ninoyu, M. Suematsu, J. Nakayama, N. Tsuchihashi, M. Okami, H. Sakata, H. Yoshihashi, T. Kobayashi, K. Kumakawa, T. Yoshida, T. Esaki, S.-I. Usami, Detailed clinical features and genotype-phenotype correlation in an OTOF-related hearing loss cohort in Japan. *Hum. Genet.* **141**, 865–875 (2022).
124. Y.-M. Zhu, Q. Li, X. Gao, Y.-F. Li, Y.-L. Liu, P. Dai, X.-P. Li, Familial temperature-sensitive auditory neuropathy: Distinctive clinical courses caused by variants of the OTOF gene. *Front. Cell Dev. Biol.* **9**, 732930 (2021).
125. A. M. Fedick, C. Jalas, A. Swaroop, E. E. Smouha, B. D. Webb, Identification of a novel pathogenic OTOF variant causative of nonsyndromic hearing loss with high frequency in the Ashkenazi Jewish population. *Appl. Clin. Genet.* **9**, 141–146 (2016).

Acknowledgments: We thank C. Senger-Freitag, S. Gerke, S. Langer, A. Schacht, and P. Räke-Kügler for excellent technical and administrative support. We are grateful to T. Cheung Cheng and R. Fernandez-Busnadiego for supporting cryo-EM data acquisition and E. Sakata and B. Vona for critically reading the manuscript. **Funding:** This work was supported and funded by Deutsche Forschungsgemeinschaft (DFG) through the Cluster of Excellence (EXC2067) Multiscale Bioimaging EXC 2067/1-390729940 (MBExC, to N.Br., C.C., H.G., T.M., C.P., and J.P.) and Collaborative Research Centers 889, 1286, and 1690 (to N.Br., T.M., C.P., J.P., N.S., and C.W.), as well as CR 937/2-1 (to C.C.) and MO896/5 to (to T.M.). Cryo-EM instrumentation was jointly funded by the DFG Major Research Instrumentation program (448415290) and the Ministry of Science and Culture of the State of Lower Saxony (main applicant: R. Fernandez-Busnadiego). A.T. is a collegian of the Hertha Sponer College of MBExC. N.E. is supported by a fellowship of the Boehringer Ingelheim Fonds. T.M. is a fellow of the Max-Planck-Society and was supported by Fondation Pour l'Audition (FPA RD-2020-10). N.S. is supported by the Heisenberg program of the German Research Foundation (STR1027/5-2). L.F. is recipient of a scholarship of the Goettingen Promotionskolleg für Medizinstudierende, funded by the Jacob-Henle-Programm or Else-Kroener-Fresenius-Stiftung (Promotionskolleg für Epigenomik und Genomdynamik, 2017_Promotionskolleg.04). C.Z.K.S. and V.P. were supported by the Institute of Cancer Research (ICR) and funded by the Wellcome Trust (220300Z/20/Z). The transmission electron microscope JEM2100Plus was procured as part of the UMG's project "ETomoH&H: Elektronentomografie an Herz und Hirn". As part of the Union's response to the COVID-19 pandemic (REACT-EU) this project was co-financed by the European Union and funded by the Lower Saxony Multifondsprogramm with funds from the "Europäischen Fonds für regionale Entwicklung (EFRE)" and the State of Lower Saxony, as well as the University Medical Center Göttingen (to C.W.). **Author contributions:** Conceptualization: T.M., H.C., C.C., and J.P. Methodology: N.Ba., H.G., F.L., C.C., V.P., T.M., C.P., J.P., N.S., and C.W. Software: C.P. Investigation: H.C., C.C., A.T., N.E., N.Ba., L.F., F.L., F.B., S.M., K.E., C.Z.K.S., and C.W. Validation: T.M., H.G., C.C., J.P., C.W., and N.S. Formal analysis: H.C., C.C., A.T., N.E., N.Ba., L.F., F.L., F.B., S.M., and K.E. Data curation: T.M., C.C., J.P., C.P., C.W., and H.C. (key author for patch-clamp and immunohistochemistry) and C.C. (key author for structural and functional analysis of purified otoferlin) contributed equally. N.S: Writing—original draft: T.M., H.C., C.C., N.E. Writing—review and editing: All authors. Visualization: H.C., C.C., A.T., and N.E. Funding acquisition: N.Br., H.G., T.M., C.C., C.P., J.P., N.S., and C.W. Resources: N.Br., H.G., T.M., C.P., J.P., N.S., and C.W. Supervision: N.Br., H.G., F.L., T.M., C.P., J.P., N.S., and C.W. Project administration: T.M., H.G., and J.P. **Competing interests:** The authors declare that they have no competing interests. **Data and materials availability:** All data needed to evaluate the conclusions in the paper are present in the paper and/or the Supplementary Materials. Electrophysiology and imaging source data have been uploaded on Zenodo (https://zenodo.org/records/16941955). Atomic coordinates and cryo-EM maps were deposited in the Protein Data Bank (PDB) and Electron Microscopy Data Bank (EMDB) under the following accession codes: lipid-bound otoferlin—30% PS and 10% PI(4,5)P_2 nanodisc: PDB 9QE2 and EMD-53046, 25% PS and 5% PI(4,5)P_2 nanodisc: PDB 9SEA and EMD-54805, merged datasets: PDB 9SFL and EMD-54827; Ca^{2+}-bound, lipid-free otoferlin – open conformation, class 1: PDB 9SEG and EMD-54809, open conformation, class 2: PDB 9SE5, EMD-54802, "closed-like" state: 9SI1, EMD-54923; Ca^{2+}- and lipid-free state: PDB 9SH0, EMD-54883. Source data related to the biochemical experiments have been deposited in a public repository (https://doi.org/10.5281/zenodo.16944849). Molecular dynamics simulation data were uploaded to Zenodo (https://doi.org/10.5281/zenodo.16936610).

Submitted 9 May 2025
Accepted 16 September 2025
Published 15 October 2025
10.1126/sciadv.ady8532
