# Supplementary material for: Structure and function of otoferlin, a synaptic protein of sensory hair cells essential for hearing
Source: Sci Adv. 2025 Oct 15;11(42):eady8532. doi: 10.1126/sciadv.ady8532 (PMC12526402; doi:10.1126/sciadv.ady8532)
Supplement: Supplementary file 2 — Figs. S1 to S15 Tables S1 to S4 Legends for movies S1 to S4 References [file sciadv.ady8532_sm.pdf]

Science Advances
AAAS

Supplementary Materials for

Structure and function of otoferlin, a synaptic protein of sensory hair cells essential for hearing

Han Chen *et al.*

Corresponding author: Tobias Moser, tmoser@gwdg.de; Julia Preobraschenski,
julia.preobraschenski@med.uni-goettingen.de

Sci. Adv. **11**, eady8532 (2025)
DOI: 10.1126/sciadv.ady8532

The PDF file includes:

Figs. S1 to S15
Tables S1 to S4
Legends for movies S1 to S4
References

Other Supplementary Material for this manuscript includes the following:

Movies S1 to S4

Figure S1

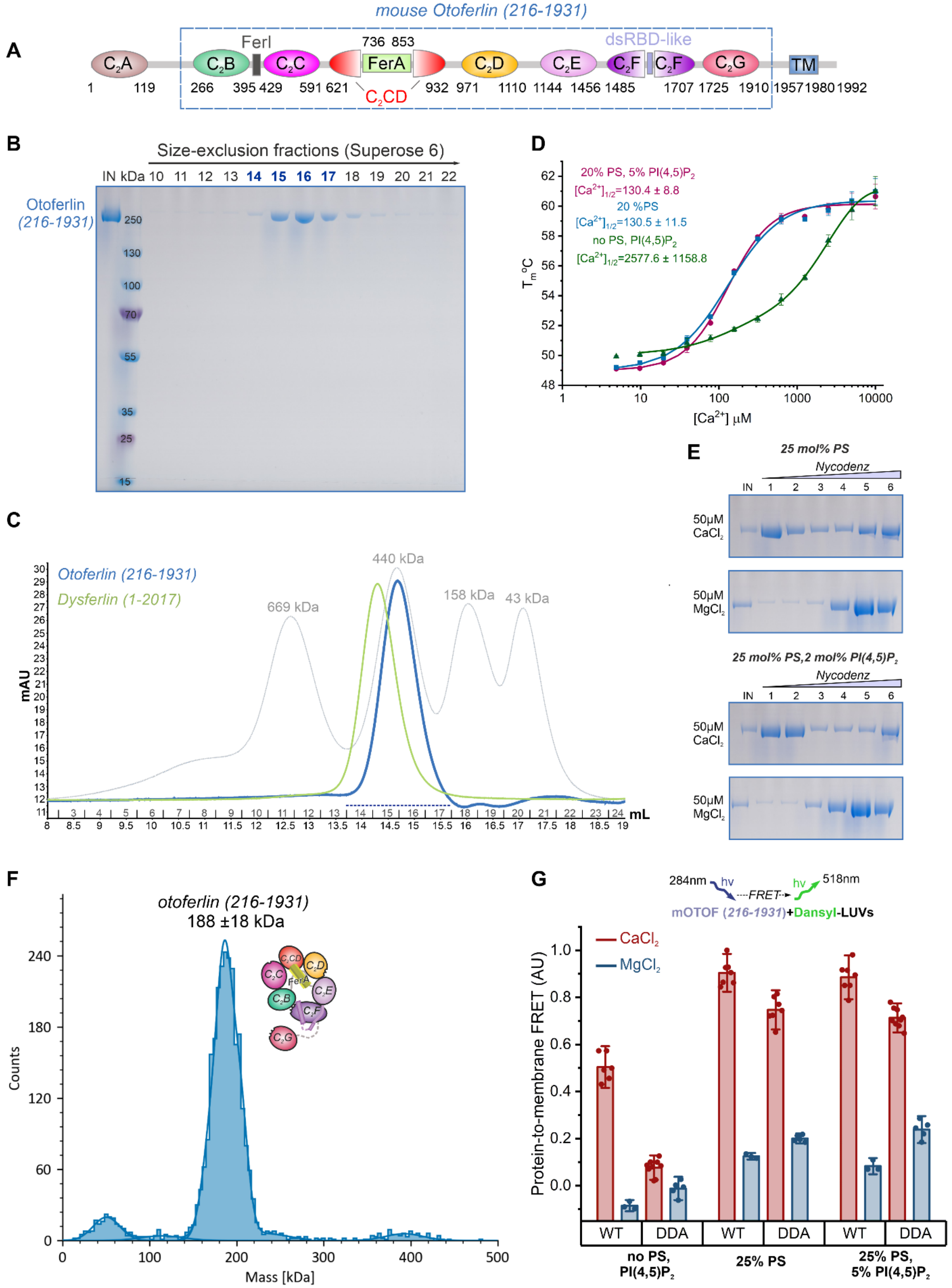

A
mouse Otoferlin (216-1931)
FerI
736 853
dsRBD-like
C2A
C2B
C2C
FerA
C2D
C2E
C2F
C2F
C2G
TM
C2CD
1 119 266 395 429 591 621 932 971 1110 1144 1456 1485 1707 1725 1910 1957 1980 1992
B
Size-exclusion fractions (Superose 6)
IN kDa 10 11 12 13 14 15 16 17 18 19 20 21 22
Otoferlin (216-1931)
250
130
100
70
55
35
25
15
C
Otoferlin (216-1931)
Dysferlin (1-2017)
440 kDa
669 kDa
158 kDa
43 kDa
mAU
mL
D
20% PS, 5% PI(4,5)P2
[Ca2+]1/2=130.4 ± 8.8
20 %PS
[Ca2+]1/2=130.5 ± 11.5
no PS, PI(4,5)P2
[Ca2+]1/2=2577.6 ± 1158.8
Tm °C
[Ca2+] µM
E
25 mol% PS
Nycodenz
IN 1 2 3 4 5 6
50µM CaCl2
50µM MgCl2
25 mol% PS,2 mol% PI(4,5)P2
Nycodenz
IN 1 2 3 4 5 6
50µM CaCl2
50µM MgCl2
F
otoferlin (216-1931)
188 ±18 kDa
Counts
Mass [kDa]
G
284nm hv FRET hv 518nm
mOTOF (216-1931)+Dansyl-LUVs
CaCl2
MgCl2
Protein-to-membrane FRET (AU)
WT DDA WT DDA WT DDA
no PS, PI(4,5)P2
25% PS
25% PS, 5% PI(4,5)P2

Figure S1. Biochemical characterization of mouse otoferlin

(A) Structure-based domain composition schematic of mouse otoferlin. The borders of the otoferlin construct (residues 216-1931) used for cryo-EM structure determination are boxed.

(B) SDS-PAGE analysis of purified mouse otoferlin (residues 216-1931) after size-exclusion chromatography (SEC).

(C) The SEC profile of mouse otoferlin (216-1931) applied to a Superose 6 column. The peak otoferlin fractions (see also B) are indicated with a dashed line and soluble dysferlin (residues 1-2017) was used as a ferlin mass reference. The SEC profile of several gel filtration standards with known molecular masses are shown in grey.

(D) Ca^{2+}-binding activity of otoferlin (216-1931) assessed in the presence of liposomes. The Ca^{2+}-binding activity was assessed using a nanoDSF assay and large unilamellar vesicles (LUVs) of indicated composition (comprising 20 mol% PS, 20 mol% PS and 5 mol% PI(4,5)P_2 or lacking anionic phospholipids, no PS, PI(4,5)P_2) were used at a 1 mM assay concentration. $[Ca^{2+}]_{1/2}$ values were estimated by nonlinear regression curve fit. Error bars represent the standard error of the mean (SEM, n=3).

(E) Ca^{2+}-sensitive liposome binding activity of mouse otoferlin (216-1931) assessed using a coflotation assay. Binding of otoferlin to LUVs of indicated acidic phospholipids composition was assessed by ultracentrifugation on a Nycodenz step in the presence of 50 µM $CaCl_2$ or $MgCl_2$. The Nycodenz gradients were harvested from the top, analyzed by SDS-PAGE, and stained with Coomassie Blue.

(F) Characterization of the soluble otoferlin (216-1931) construct by mass photometry. The distribution of binding events is consistent with the presence of a major otoferlin (216-1931) species having a molecular mass of 188±18 kDa. The measurements were carried out in the absence of $CaCl_2$ and were repeated four times (n=4).

(G) Liposome binding activity of mOTOF (216-1931) and mOTOF (216-1931)-D1829A/D1831A (DDA) mutant using a FRET-based assay. The measurements were carried out in the presence of 250 µM $CaCl_2$ or $MgCl_2$ at 0.25 µM and 50 µM protein and liposome concentrations, respectively. The measured LUVs comprised 5 mol% Dansyl-PE. The measurements in the presence of $CaCl_2$ were repeated n=6 (WT, in the presence of liposomes lacking PS and PI(4,5)P_2, denoted as no PS, PI(4,5)P_2), n=9 (DDA, in the presence of liposomes comprising 25 mol% PS and 5 mol% PI(4,5)P_2 or lacking PS and PI(4,5)P_2), or n=7 (remaining measurements). The measurements in the presence of $MgCl_2$ were carried out in n=3 (WT) or n=5 (DDA) replicates. The data were normalized to the maximum Dansyl-PE emission after subtraction of the background at each individual point (using protein-free measurements) and error bars indicate the standard deviation (SD) from the mean (±1.5 SD). See also Figure 3G.

Figure S2

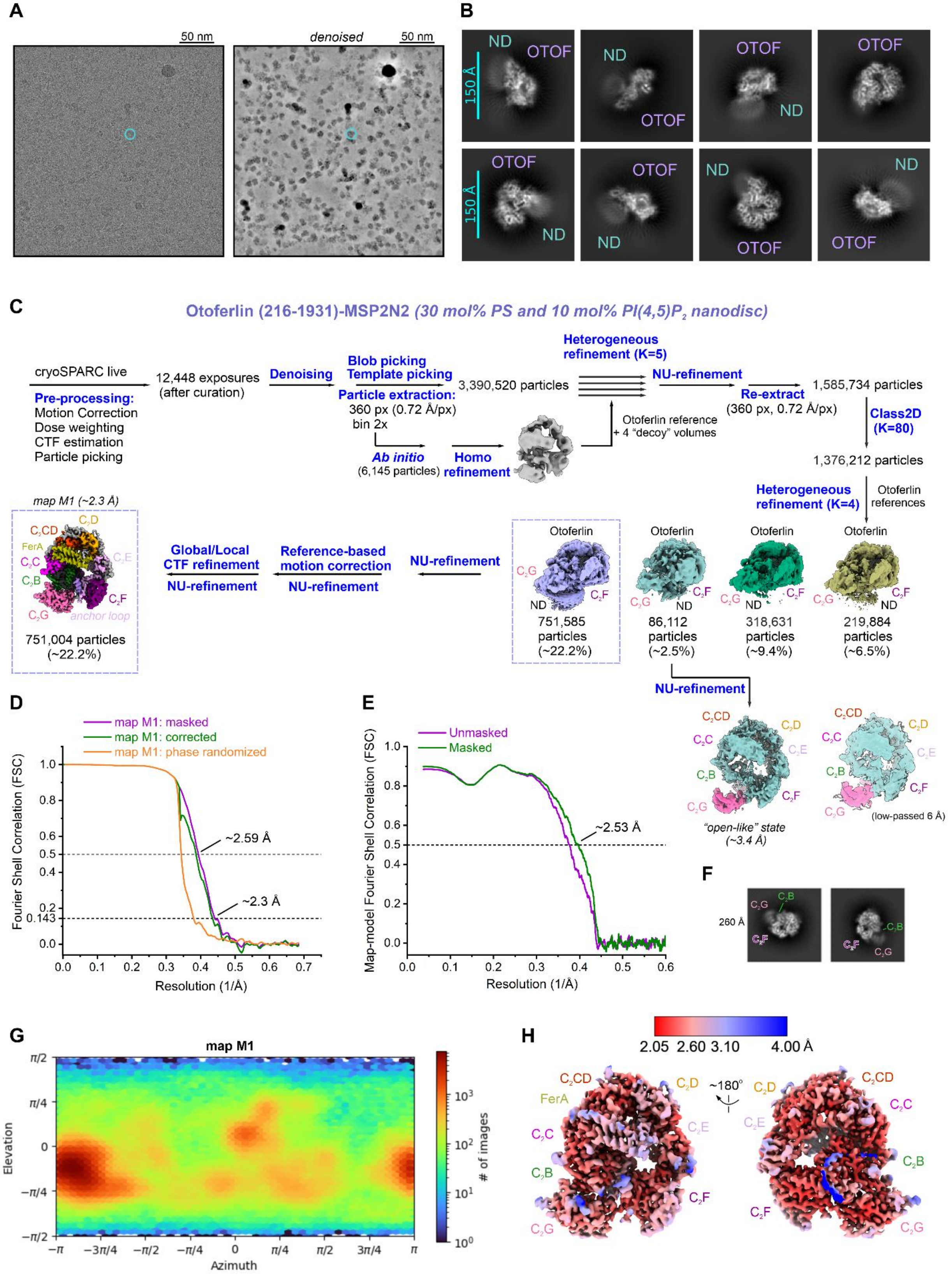

A
50 nm
denoised
50 nm
B
ND
OTOF
150 Å
C
Otoferlin (216-1931)-MSP2N2 (30 mol% PS and 10 mol% PI(4,5)P2 nanodisc)
cryoSPARC live
Pre-processing:
Motion Correction
Dose weighting
CTF estimation
Particle picking
12,448 exposures (after curation)
Denoising
Blob picking
Template picking
Particle extraction:
360 px (0.72 Å/px)
bin 2x
Ab initio
(6,145 particles)
Homo refinement
3,390,520 particles
Heterogeneous refinement (K=5)
Otoferlin reference + 4 "decoy" volumes
NU-refinement
Re-extract
(360 px, 0.72 Å/px)
1,585,734 particles
Class2D (K=80)
1,376,212 particles
Heterogeneous refinement (K=4)
Otoferlin references
map M1 (~2.3 Å)
751,004 particles (~22.2%)
Global/Local CTF refinement
NU-refinement
Reference-based motion correction
NU-refinement
NU-refinement
Otoferlin
751,585 particles (~22.2%)
86,112 particles (~2.5%)
318,631 particles (~9.4%)
219,884 particles (~6.5%)
"open-like" state (~3.4 Å)
(low-passed 6 Å)
D
map M1: masked
map M1: corrected
map M1: phase randomized
~2.59 Å
~2.3 Å
Fourier Shell Correlation (FSC)
Resolution (1/Å)
E
Unmasked
Masked
~2.53 Å
Map-model Fourier Shell Correlation (FSC)
Resolution (1/Å)
F
260 Å
G
map M1
Elevation
Azimuth
# of images
H
2.05 2.60 3.10 4.00 Å
~180°
FerA
anchor loop

Figure S2. Cryo-EM image analysis of the otoferlin (216-1931)-nanodisc complex

(30 mol% PS and 10 mol% PI(4,5)P_2 MSP2N2 nanodisc)

(A) Cryo-EM micrograph (raw and denoised) of the vitrified otoferlin (216-1931)-MSP2N2 complex. The MSP2N2 nanodisc comprised of 30 mol% DOPS and 10 mol% PI(4,5)P_2. A typical otoferlin particle is circled.

(B) Reference-free 2D class averages of the otoferlin (216-1931)-MSP2N2 nanodisc complex. The nanodisc-bound bound otoferlin (OTOF) and the nanodisc (ND) are indicated. The images of the 2D class averages were generated using the freely available cryosparc-tools Python package (https://tools.cryosparc.com/).

(C) Cryo-EM processing routine of otoferlin (216-1931)-MSP2N2 nanodisc complex (30 mol% DOPS and 10 mol% PI(4,5)P_2 nanodisc). The resolution estimates (FSC=0.143 criterion) of the final maps are indicated, and the final maps are coloured according to the modelled domains (see also Fig. S1A).

(D) Fourier shell correlation (FSC) plots between the otoferlin-MSP2N2 cryo-EM half-maps. The global resolution of the map (map M1) was estimated using the gold-standard FSC criterion (FSC=0.143).

(E) Map versus model FSC plot for the map M1 of the otoferlin (216-1931)-MSP2N2 complex (30 mol% DOPS and 10 mol% PI(4,5)P_2).

(F) Reference-free 2D class averages of lipid-bound otoferlin particles assigned to the "open-like" conformational state. In this "open-like" state, observed for ~2.5% of otoferlin-nanodisc particles, C_2G is dynamic and does not interact with the C_2B domain.

(G) Angular distribution of the otoferlin-MSP2N2 particles contributing to the map M1 of the complex. Red colour indicates a higher number of particle images.

(H) Local resolution of the lipid-bound otoferlin cryo-EM map (map M1). Red colour indicates a higher resolution of the map. The otoferlin domains are indicated.

Figure S3

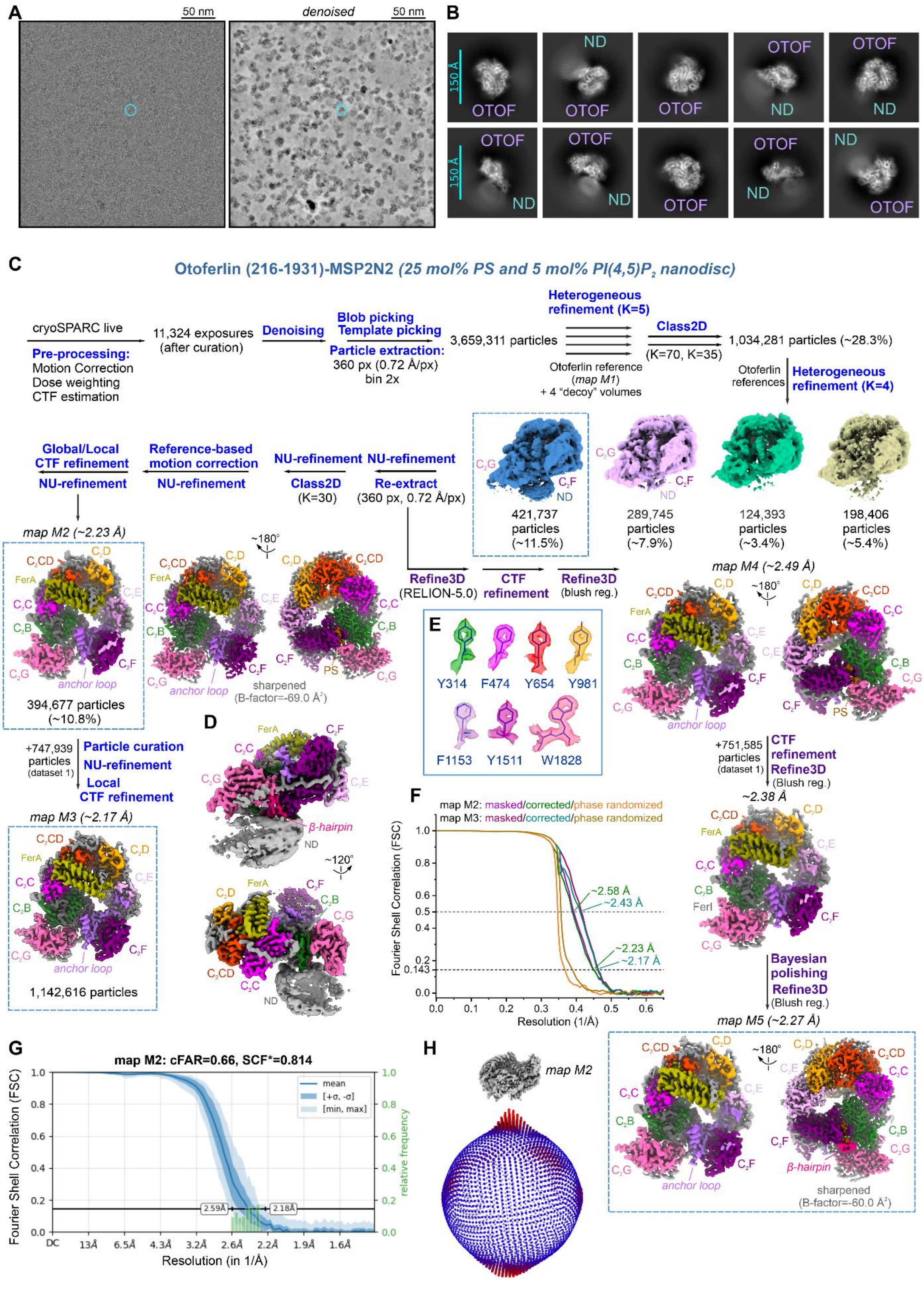
A
50 nm
denoised
50 nm
B
150 Å
OTOF
ND
C
Otoferlin (216-1931)-MSP2N2 (25 mol% PS and 5 mol% PI(4,5)P2 nanodisc)
cryoSPARC live
Pre-processing:
Motion Correction
Dose weighting
CTF estimation
11,324 exposures (after curation)
Denoising
Blob picking
Template picking
Particle extraction:
360 px (0.72 Å/px) bin 2x
3,659,311 particles
Heterogeneous refinement (K=5)
Otoferlin reference (map M1) + 4 "decoy" volumes
Class2D
(K=70, K=35)
1,034,281 particles (~28.3%)
Otoferlin references
Heterogeneous refinement (K=4)
421,737 particles (~11.5%)
289,745 particles (~7.9%)
124,393 particles (~3.4%)
198,406 particles (~5.4%)
NU-refinement
Re-extract (360 px, 0.72 Å/px)
NU-refinement
Class2D (K=30)
Reference-based motion correction
NU-refinement
Global/Local CTF refinement
NU-refinement
map M2 (~2.23 Å)
394,677 particles (~10.8%)
sharpened (B-factor=-69.0 Å²)
anchor loop
+747,939 particles (dataset 1)
Particle curation
NU-refinement
Local CTF refinement
map M3 (~2.17 Å)
1,142,616 particles
Refine3D (RELION-5.0)
CTF refinement
Refine3D (blush reg.)
map M4 (~2.49 Å)
+751,585 particles (dataset 1)
CTF refinement
Refine3D (Blush reg.)
~2.38 Å
Bayesian polishing
Refine3D (Blush reg.)
map M5 (~2.27 Å)
sharpened (B-factor=-60.0 Å²)
β-hairpin
D
E
Y314
F474
Y654
Y981
F1153
Y1511
W1828
F
map M2: masked/corrected/phase randomized
map M3: masked/corrected/phase randomized
~2.58 Å
~2.43 Å
~2.23 Å
~2.17 Å
Fourier Shell Correlation (FSC)
Resolution (1/Å)
G
map M2: cFAR=0.66, SCF*=0.814
mean
[+σ, -σ]
[min, max]
relative frequency
2.59Å
2.18Å
Resolution (in 1/Å)
H
map M2

Figure S3. Cryo-EM analysis of the otoferlin (216-1931)-nanodisc complex

(25 mol% PS and 5 mol% PI(4,5)P_2 nanodisc)

(A) Cryo-EM micrographs (raw and denoised) of the otoferlin (216-1931) bound to a 25 mol% DOPS and 5 mol% PI(4,5)P_2 MSP2N2 nanodisc. A lipid-bound otoferlin particle is circled for orientation purposes.

(B) 2D class averages of the otoferlin (216-1931)-nanodisc complex (25 mol% DOPS and 5 mol% PI(4,5)P_2 nanodisc). The density elements corresponding to otoferlin and the MSP2N2 nanodisc are indicated.

(C) Cryo-EM data processing schematic for the lipid-bound otoferlin (216-1931) complex, assembled on a nanodisc with 25 mol% DOPS and 5 mol% PI(4,5)P_2. Processing steps shown in blue and purple were performed using cryoSPARC v.4.7 and RELION-5.0, respectively. The final maps used for model building are colour-coded by the resolved domains.

(D) Feature-enhanced map of the otoferlin (216-1931)-nanodisc complex. The locally scaled map was generated with LocScale and allows the better visualization of the low-resolution nanodisc density.

(E) Selected density snapshots from the high-resolution lipid-bound otoferlin (216-1931) cryo-EM map (map M9). The sharpened cryo-EM density map is contoured around several well-resolved otoferlin residues and coloured according to the otoferlin domain.

(F) Fourier shell correlation (FSC) plots between the cryo-EM half-maps of lipid-bound otoferlin (216-1931). The global resolution of the maps was estimated using the gold-standard FSC criterion (FSC=0.143).

(G) Fourier sampling and directional resolution of the lipid-bound otoferlin cryo-EM map (map M2). The sampling compensation factor (SCF) and the conical FSC area ratio (cFAR) were calculated using cryoSPARC v4.7.

(H) Angular distribution of the nanodisc-bound otoferlin (216-1931) particles used to reconstruct the map M2 of the complex. A higher number of particle images is indicated in red.

Figure S4

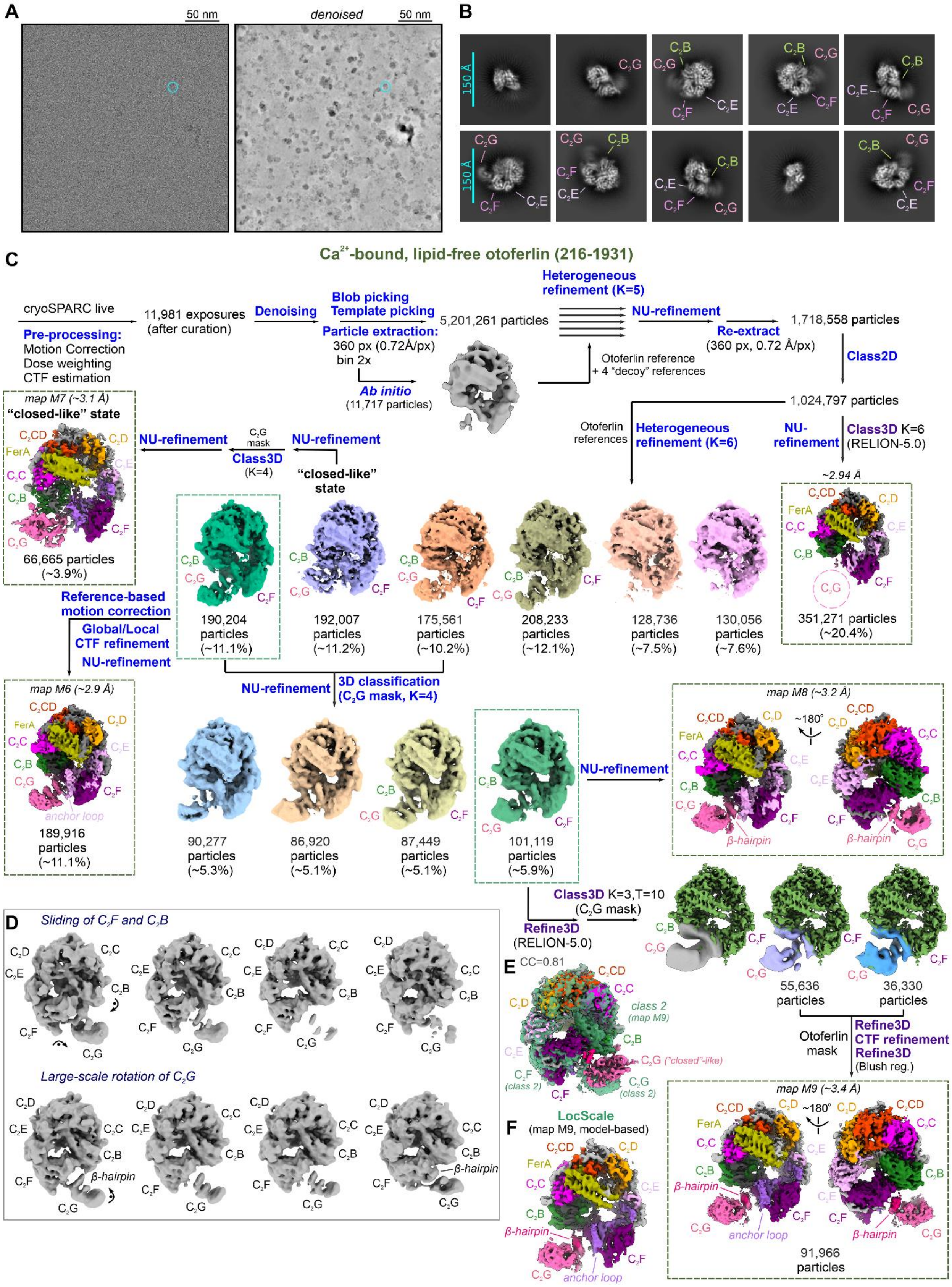

A
50 nm
denoised
50 nm
B
150 Å
Ca2+-bound, lipid-free otoferlin (216-1931)
C
cryoSPARC live
Pre-processing:
Motion Correction
Dose weighting
CTF estimation
11,981 exposures
(after curation)
Denoising
Blob picking
Template picking
Particle extraction:
360 px (0.72Å/px)
bin 2x
Ab initio
(11,717 particles)
5,201,261 particles
Heterogeneous
refinement (K=5)
Otoferlin reference
+ 4 "decoy" references
NU-refinement
1,718,558 particles
Re-extract
(360 px, 0.72 Å/px)
Class2D
1,024,797 particles
Otoferlin
references
Heterogeneous
refinement (K=6)
NU-
refinement
Class3D K=6
(RELION-5.0)
~2.94 Å
351,271 particles
(~20.4%)
map M7 (~3.1 Å)
"closed-like" state
66,665 particles
(~3.9%)
NU-refinement
C2G mask
Class3D
(K=4)
NU-refinement
"closed-like" state
190,204 particles (~11.1%)
192,007 particles (~11.2%)
175,561 particles (~10.2%)
208,233 particles (~12.1%)
128,736 particles (~7.5%)
130,056 particles (~7.6%)
Reference-based
motion correction
Global/Local
CTF refinement
NU-refinement
map M6 (~2.9 Å)
anchor loop
189,916
particles
(~11.1%)
NU-refinement
3D classification
(C2G mask, K=4)
90,277 particles (~5.3%)
86,920 particles (~5.1%)
87,449 particles (~5.1%)
101,119 particles (~5.9%)
NU-refinement
map M8 (~3.2 Å)
~180°
β-hairpin
Class3D K=3,T=10
(C2G mask)
Refine3D
(RELION-5.0)
55,636
particles
36,330
particles
Otoferlin
mask
Refine3D
CTF refinement
Refine3D
(Blush reg.)
map M9 (~3.4 Å)
91,966
particles
D
Sliding of C2F and C2B
Large-scale rotation of C2G
E
CC=0.81
class 2
(map M9)
C2G ("closed"-like)
(class 2)
F
LocScale
(map M9, model-based)
C2CD
C2D
C2C
C2B
C2E
C2F
C2G
FerA

Figure S4. Cryo-EM analysis of Ca^{2+}-bound, lipid-free otoferlin (216-1931)

(A) Denoised cryo-EM micrograph of lipid-free otoferlin (216-1931) imaged in vitreous ice. A typical otoferlin (216-1931) particle is circled in cyan.

(B) Reference-free 2D class averages of the lipid-free otoferlin (216-1931). Note that the C-terminal C_2G domain is dynamic in the absence of a lipid bilayer (see also Fig. S2B).

(C) Cryo-EM processing schematic for the lipid-free otoferlin (216-1931). The resolution of the final maps was estimated using the FSC=0.143 criterion. The final lipid-free otoferlin (216-1931) maps were colored as in Figure S2C.

(D) Conformational dynamics of otoferlin (216-1931) in the lipid-free state. Selected map snapshots along the two resolved modes of 3D variability are shown side by side. Particle motions in the lipid-free state primarily involve the C_2B, C_2F, and the highly dynamic C_2G domain.

(E) Superposition of the "open" and "closed"-like maps of Ca^{2+}-bound, lipid-free otoferlin. The "closed"-like state map (map M7) is colour-coded by modelled domains, while the "open" state map (class 2, map M9) is shown in light green.

(F) Locally scaled map of Ca^{2+}-bound, lipid-free otoferlin. The map (map M9, class 2) was scaled with LocScale using the refined structural model (model-based) to visualize the dynamic C_2G domain.

Figure S5

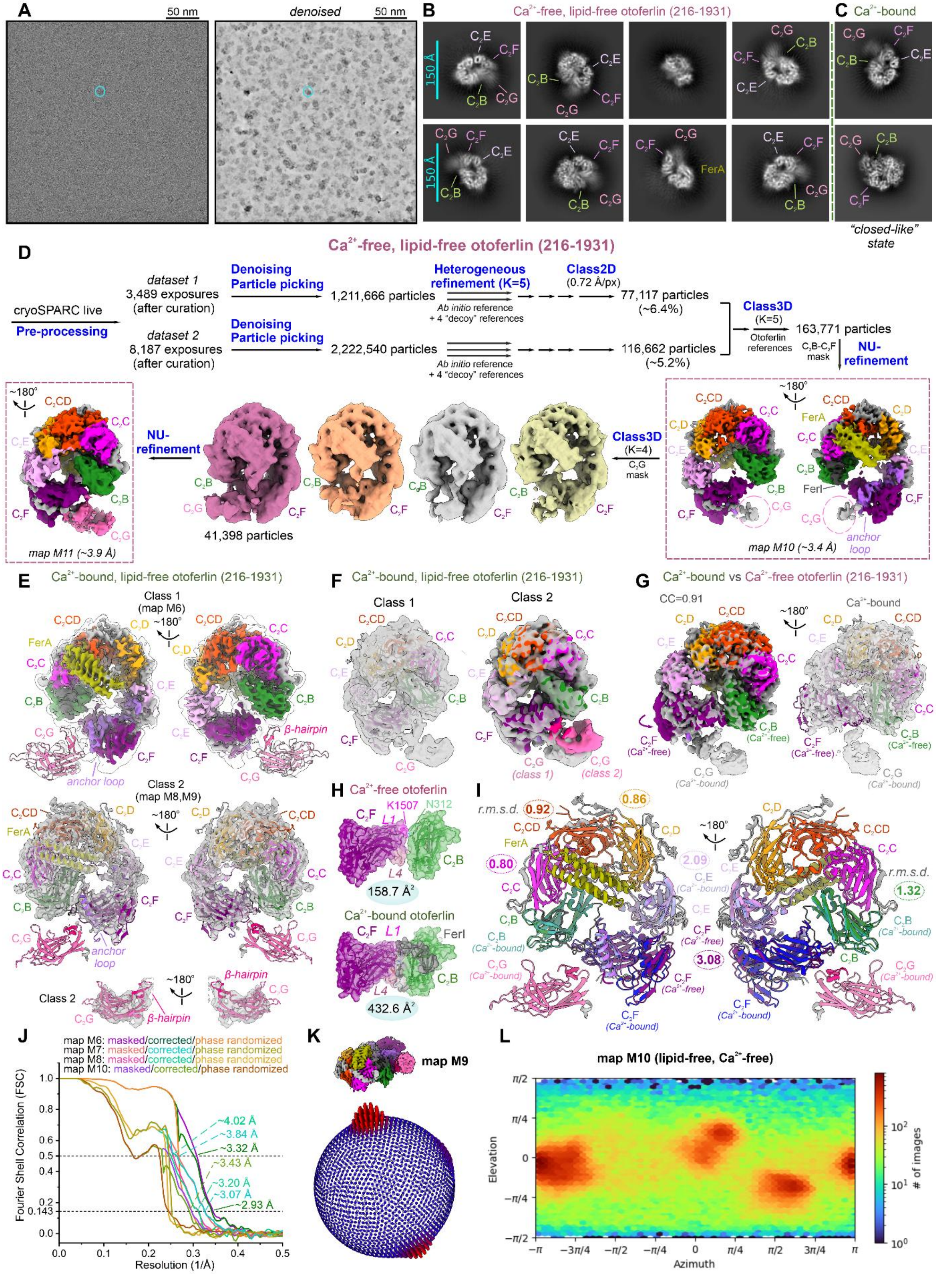
A
50 nm
denoised
50 nm
B
Ca2+-free, lipid-free otoferlin (216-1931)
C
Ca2+-bound
150 Å
"closed-like" state
D
Ca2+-free, lipid-free otoferlin (216-1931)
cryoSPARC live
Pre-processing
dataset 1
3,489 exposures (after curation)
dataset 2
8,187 exposures (after curation)
Denoising
Particle picking
1,211,666 particles
2,222,540 particles
Heterogeneous refinement (K=5)
Ab initio reference + 4 "decoy" references
Class2D (0.72 Å/px)
77,117 particles (~6.4%)
116,662 particles (~5.2%)
Class3D (K=5)
Otoferlin references
163,771 particles
NU-refinement
Class3D (K=4)
41,398 particles
map M11 (~3.9 Å)
map M10 (~3.4 Å)
anchor loop
E
Ca2+-bound, lipid-free otoferlin (216-1931)
Class 1 (map M6)
Class 2 (map M8,M9)
β-hairpin
F
Ca2+-bound, lipid-free otoferlin (216-1931)
Class 1
Class 2
G
Ca2+-bound vs Ca2+-free otoferlin (216-1931)
CC=0.91
H
Ca2+-free otoferlin
K1507
N312
158.7 Å2
Ca2+-bound otoferlin
432.6 Å2
I
r.m.s.d.
0.92
0.86
0.80
2.09
1.32
3.08
J
map M6: masked/corrected/phase randomized
map M7: masked/corrected/phase randomized
map M8: masked/corrected/phase randomized
map M10: masked/corrected/phase randomized
Fourier Shell Correlation (FSC)
Resolution (1/Å)
~4.02 Å
~3.84 Å
~3.32 Å
~3.43 Å
~3.20 Å
~3.07 Å
~2.93 Å
K
map M9
L
map M10 (lipid-free, Ca2+-free)
Elevation
Azimuth
# of images

Figure S5. Cryo-EM image analysis of Ca^{2+}-free, lipid-free otoferlin (216-1931)

(A) Cryo-EM micrograph (raw and denoised) of vitrified otoferlin (residues 216-1931) in the Ca^{2+}-free, lipid-free state. A typical otoferlin particle is circled.

(B) Representative 2D class averages of otoferlin (216-1931) imaged in the absence of Ca^{2+} and lipids. The assigned otoferlin domains are indicated.

(C) 2D class averages of lipid-free otoferlin (216-1931) imaged in the presence of Ca^{2+} (see also Fig. S2B). These 2D averages are shown for comparison with the Ca^{2+}-free 2D classes in panel B; additional 2D averages are provided in Fig. S2B. Note the "closed"-like 2D classes observed in the presence of Ca^{2+}, which may indicate that Ca^{2+} ions stabilize the poses of the C_2F and C_2G domains (see also Fig. S4C and S4E).

(D) Cryo-EM image processing schematic for the Ca^{2+}-free otoferlin (216-1931). Key processing steps are indicated, and the final maps are coloured according to the modelled otoferlin domains.

(E) The two resolved 3D classes of Ca^{2+}-bound lipid-free otoferlin (216-1931). The two conformations of lipid-free otoferlin differ in the orientation of the C-terminal C_2G domain and correspond to "open" otoferlin states. The final models were fitted into the refined cryo-EM maps and colour-coded. The flexible C_2G domain was fitted into either a 6 Å (top panel) or 3.9 Å (bottom panel) low-pass filtered maps, which are shown as transparent surfaces.

(F) Comparison between the two resolved states of Ca^{2+}-bound, lipid-free otoferlin (216-1931). The two otoferlin maps were aligned and the similar C_2B-C_2F domains are shown as either model (left) or cryo-EM density maps (right).

(G) Comparison between the Ca^{2+}-free and Ca^{2+}-bound otoferlin (216-1931) maps in the lipid-free state. Note the different orientation of the C_2F domain and the absence of density (or weak density in map M11, S5D) for the C_2G domain in the Ca^{2+}-free state. Cryo-EM maps and models are depicted as in E-F.

(H) The C_2B-C_2F interface in the Ca^{2+}-free and Ca^{2+}-bound, lipid-free otoferlin. The C_2B and C_2F domains are shown as solvent-excluded surfaces, and interface areas are indicated (see also Fig. 4F).

(I) Structural superposition of Ca^{2+}-free and Ca^{2+}-bound, lipid-free otoferlin. The C_2B and C_2E-C_2G domains are shown in different colours. Root-mean-square deviations (r.m.s.d.) of the domains' backbone (Cα) between the two lipid-free conformations are indicated.

(J) Fourier shell correlation (FSC) plots for the lipid-free otoferlin (216-1931) cryo-EM maps. FSCs were calculated between cryo-EM half-maps and the resolution of the maps were estimated according to the gold-standard FSC criterion (FSC=0.143).

(K-L) Angular distribution of the lipid-free otoferlin particles in the final Ca^{2+}-bound (map M9) and Ca^{2+}-free (map M10) cryo-EM maps. Higher particle numbers at given projection angles are indicated in red.

Figure S6

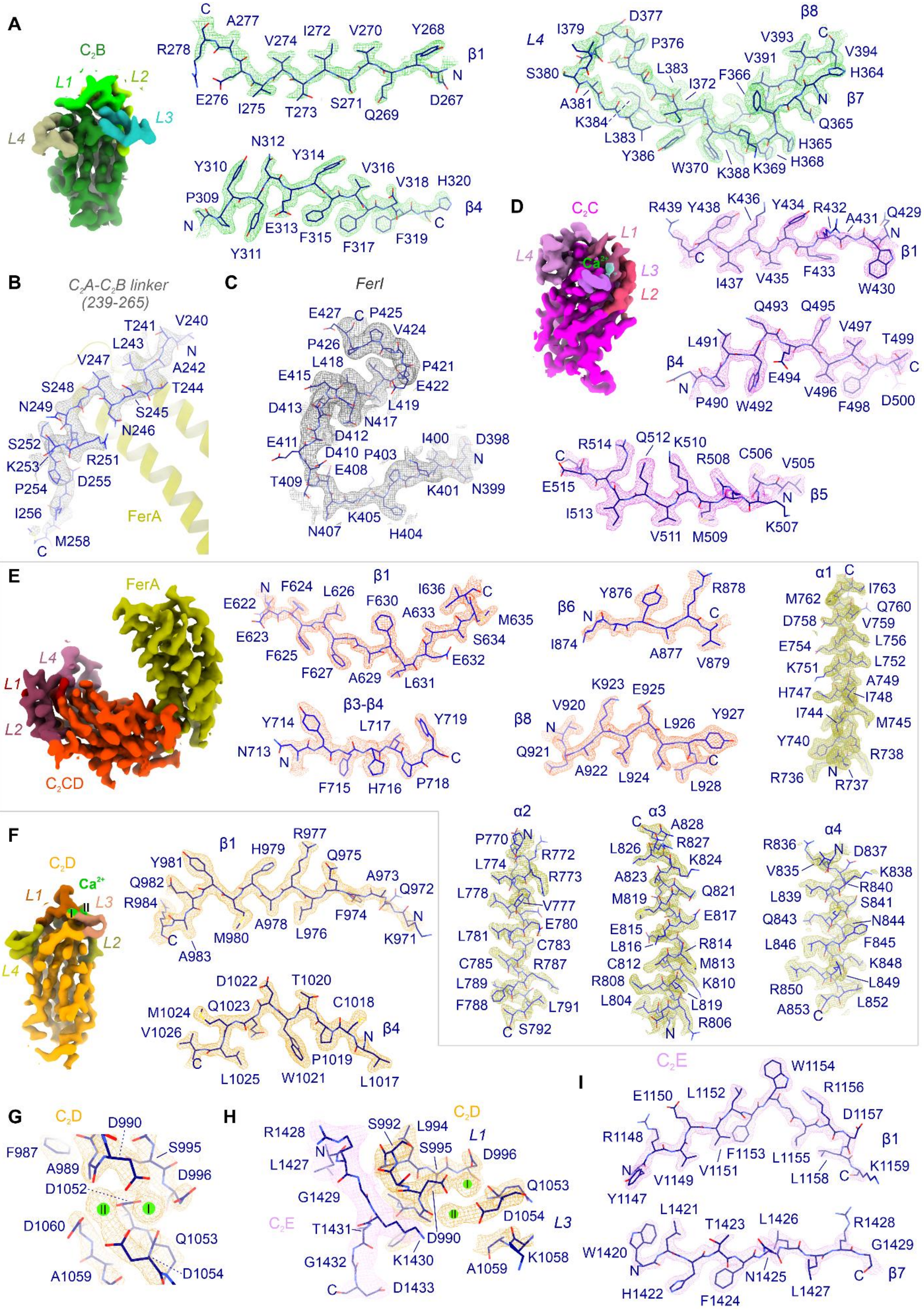
A
C2B
L1
L2
L3
L4
β1
β4
β7
β8
B
C2A-C2B linker (239-265)
FerA
C
FerI
D
C2C
Ca2+
β1
β4
β5
E
FerA
C2CD
β1
β3-β4
β6
β8
α1
α2
α3
α4
F
C2D
Ca2+
β1
β4
G
C2D
H
C2D
C2E
I
C2E
β1
β7

Figure S6. Cryo-EM densities of otoferlin's core domains (C_2B-C_2D) in the lipid-bound state

(A) Cryo-EM density of the C_2B domain in the lipid-bound state of otoferlin (216-1931). The top loops of C_2B (L1-L4) are colour-coded. Selected β-strand densities are contoured around the fitted model.
(B) The resolved C_2A-C_2B linker (residues 239-265) in the "closed", lipid-bound state of otoferlin (216-1931). The modelled C_2A-C_2B linker (depicted as sticks with its cryo-EM density) engages the α-helices of the FerA module at multiple sites before reaching the membrane-bound N-terminal C_2B domain.
(C) Cryo-EM density of the FerI motif in otoferlin's lipid-bound state. The FerI motif (residues 396-428) connects the N-terminal C_2B to the C_2C domain; the fitted FerI model is shown with the cryo-EM density.
(D) Cryo-EM density of the Ca^{2+}-bound C_2C domain. The cryo-EM density (cyan) is contoured around the single bound Ca^{2+} ion, and the top loops (L1-L4) are indicated. Several β-strands of C_2C are shown with their cryo-EM densities.
(E) Structure of otoferlin's C_2CD-FerA module. The C_2CD-FerA region packs between the C_2C and C_2D domains. Cryo-EM densities of FerA's four-helix bundle and of selected β-strands from C_2CD are shown with the final models.
(F) Cryo-EM density of otoferlin's C_2D domain. C_2D coordinates two Ca^{2+} ions independently of phospholipid binding. Two representative β-strands are shown as stick models with their cryo-EM densities.
(G-H) The two Ca^{2+}-binding sites of otoferlin's C_2D domain. The Ca^{2+}-coordinating residues of C_2D (G) and the neighboring L4 loop of C_2E (H) are shown as sticks with the contoured cryo-EM density.
(I) Cryo-EM densities of selected β-strands in the C_2E domain. The β-strands are depicted as sticks, and the density is contoured around the C_2E model.

Figure S7

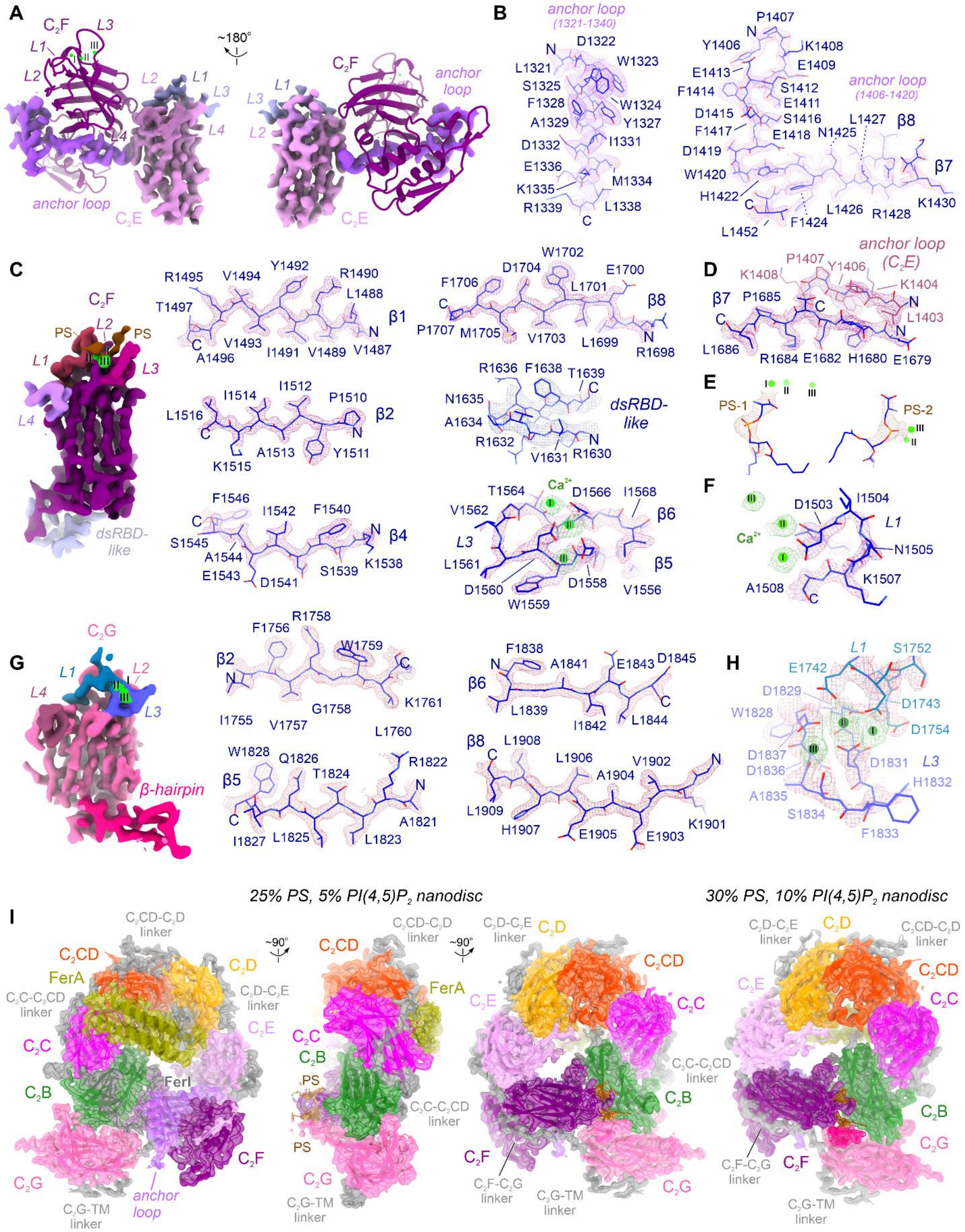
A
C2F
L3
L1
L2
L4
L2
L1
L3
L4
anchor loop
C2E
~180°
C2F
anchor loop
L1
L3
L2
C2E
B
anchor loop
(1321-1340)
D1322
W1323
L1321
S1325
F1328
W1324
A1329
Y1327
I1331
D1332
E1336
M1334
K1335
R1339
L1338
P1407
Y1406
K1408
E1409
E1413
S1412
F1414
E1411
anchor loop
(1406-1420)
D1415
S1416
F1417
E1418
L1427
N1425
β8
D1419
W1420
β7
H1422
L1452
F1424
L1426
R1428
K1430
C
C2F
PS
L2
PS
L1
L3
L4
dsRBD-like
R1495
V1494
Y1492
R1490
L1488
β1
T1497
A1496
V1493
I1491
V1489
V1487
I1514
I1512
P1510
L1516
β2
A1513
Y1511
K1515
F1546
I1542
F1540
β4
S1545
A1544
E1543
D1541
S1539
K1538
W1702
D1704
E1700
F1706
L1701
β8
P1707
M1705
V1703
L1699
R1698
R1636
F1638
T1639
N1635
dsRBD-like
A1634
R1632
V1631
R1630
Ca2+
T1564
D1566
I1568
V1562
β6
L3
L1561
β5
D1560
D1558
V1556
W1559
D
anchor loop
(C2E)
K1408
P1407
Y1406
K1404
β7
P1685
L1403
L1686
R1684
E1682
H1680
E1679
E
PS-1
PS-2
F
D1503
I1504
L1
Ca2+
N1505
K1507
A1508
G
C2G
L1
L2
L4
L3
β-hairpin
β2
F1756
R1758
W1759
I1755
V1757
G1758
K1761
L1760
W1828
Q1826
T1824
R1822
β5
I1827
L1825
L1823
A1821
β6
F1838
A1841
E1843
D1845
L1839
I1842
L1844
β8
L1908
L1906
V1902
A1904
L1909
H1907
E1905
E1903
K1901
H
L1
S1752
E1742
D1829
W1828
D1743
D1754
D1837
D1836
D1831
L3
H1832
A1835
S1834
F1833
I
25% PS, 5% PI(4,5)P2 nanodisc
30% PS, 10% PI(4,5)P2 nanodisc
C2CD-C2D linker
C2CD
C2D
FerA
C2C-C2CD linker
C2D-C2E linker
C2E
C2C
C2B
FerI
C2F
C2G
C2G-TM linker
anchor loop
~90°
PS
C2C-C2CD linker
C2D-C2E linker
C2F-C2G linker

Figure S7. Cryo-EM densities of otoferlin's C-terminal domains (C_2E-C_2G) in the lipid-bound state

(A) Cryo-EM density of the C_2E domain. C_2E links the otoferlin core (C_2B-C_2D) to the C-terminal C_2F-C_2G domains. C_2E contains a large insertion loop (the "anchor loop", residues 1238-1421) between the β6 and β7 strands, which is mostly unstructured and wraps around C_2F (purple).
(B) Cryo-EM densities of selected anchor loop elements in the lipid-bound state of otoferlin. Resolved regions include a helical element (residues 1321-1340) and several segments connecting the β6-β7 strands (residues 1238-1265 and 1398-1421). Cryo-EM densities of these regions are shown with the corresponding models.
(C) Structure of the C-terminal C_2F domain in the lipid-bound state of otoferlin. Three bound Ca^{2+} ions, two PS (phosphatidylserine) molecules, and the top loops (L1-L4) are indicated. Cryo-EM densities of selected β-strands and modelled Ca^{2+} ions are contoured around the C_2F model.
(D) Contact interface between the C_2E anchor loop and the β7 strand of C_2F. The modelled otoferlin elements are shown as sticks with contoured cryo-EM densities.
(E) Cryo-EM densities of the two molecules PS molecules originating from the lipid nanodisc. The phospholipid headgroups are bound to C_2F through three coordinated Ca^{2+} ions, shown as spheres for orientation purposes.
(F) The three Ca^{2+}-binding sites of C_2F. C_2F coordinates three Ca^{2+} ions via residues in the L1-L3 loops and two recruited PS molecules (E). The cryo-EM density is contoured around the modelled ions.
(G) Cryo-EM density of the C-terminal C_2G domain in the lipid-bound state of otoferlin. The modelled Ca^{2+} ions, the top loops (L1-L4), and the β-hairpin motif of C_2G are highlighted. Several β-strands are shown as cryo-EM densities.
(H) The Ca^{2+}-binding sites of otoferlin's C_2G domain. As in C_2F, the L1-L3 loops of C_2G coordinate three Ca^{2+} ions (shown as spheres with contoured densities). The Ca^{2+} ions were likely coordinated independent of phospholipids, as no phospholipid headgroups could be modelled in the C_2G cryo-EM map. The Ca^{2+}-coordinating residues are shown as sticks.
(I) Modelling of otoferlin (216-1931) in the lipid-bound state. Final cryo-EM maps of the two otoferlin-nanodisc complexes (map M1 and map M2), assembled on either 25 mol% PS and 5 mol% PI(4,5)P_2 or 30 mol% PS and 10 mol% PI(4,5)P_2 MSP2N2 nanodiscs, are coloured according to the modelled otoferlin domains. The final otoferlin models are shown inside the maps. Resolved linker regions connecting the C_2 domains are coloured grey.

Figure S8

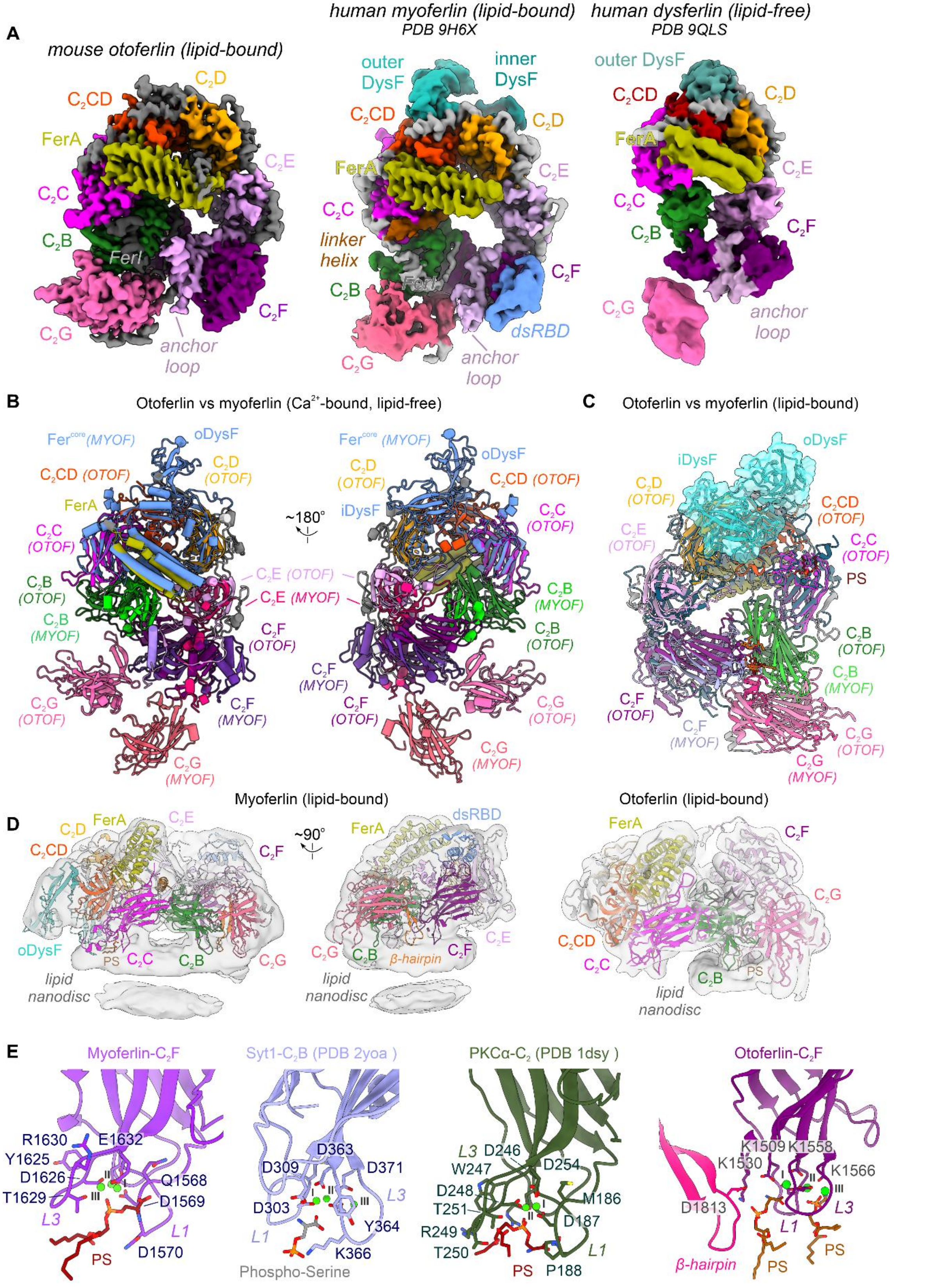

A
mouse otoferlin (lipid-bound)
human myoferlin (lipid-bound)
PDB 9H6X
human dysferlin (lipid-free)
PDB 9QLS
C$_2$CD
C$_2$D
FerA
C$_2$E
C$_2$C
C$_2$B
FerI
C$_2$F
C$_2$G
anchor loop
outer DysF
inner DysF
linker helix
dsRBD
B
Otoferlin vs myoferlin (Ca^{2+}-bound, lipid-free)
Fer^{core} (MYOF)
oDysF
C$_2$CD (OTOF)
C$_2$D (OTOF)
FerA
C$_2$C (OTOF)
C$_2$B (OTOF)
C$_2$B (MYOF)
C$_2$E (OTOF)
C$_2$E (MYOF)
C$_2$F (OTOF)
C$_2$F (MYOF)
C$_2$G (OTOF)
C$_2$G (MYOF)
~180°
iDysF
C
Otoferlin vs myoferlin (lipid-bound)
iDysF
oDysF
PS
D
Myoferlin (lipid-bound)
~90°
dsRBD
β-hairpin
PS
lipid nanodisc
Otoferlin (lipid-bound)
E
Myoferlin-C$_2$F
R1630
E1632
Y1625
D1626
Q1568
T1629
D1569
L3
L1
D1570
PS
Syt1-C$_2$B (PDB 2yoa)
D363
D309
D371
D303
Y364
K366
Phospho-Serine
PKCα-C$_2$ (PDB 1dsy)
L3
D246
W247
D254
D248
T251
M186
R249
D187
T250
P188
Otoferlin-C$_2$F
K1509
K1558
K1530
K1566
D1813
β-hairpin

Figure S8. Comparison between the cryo-EM structures of otoferlin, myoferlin, and dysferlin

(A) Side-by-side comparison between the cryo-EM structures of mouse otoferlin (lipid-bound state, residues 216-1931, this work), human myoferlin (lipid-bound state, residues 1-1997, PDB 9H6X), and human dysferlin (Ca^{2+}-bound, lipid-free state, residues 1-2017, PDB 9QLS). The cryo-EM maps are coloured after the modelled domains and are viewed from the solvent-facing side.
(B) Structural superposition of mouse otoferlin (216-1931) and human myoferlin (1-1997, 9QLN) in their Ca^{2+}-bound, lipid-free states. While the ferlin core domains (C_2C-C_2CD-FerA-C_2D) have a similar organization, the two structures differ in the positions of the N-terminal C_2B and the C_2E-C_2G domains. C_2B and C_2E-C_2G are highlighted in different colours. Note that the DysF motifs, the outer Dysf (oDysF) and the inner DysF (iDysF), are absent in otoferlin.
(C) Superposition of lipid-bound mouse otoferlin (216-1931) and human myoferlin (1-1997, PDB 9H6X/PDB 9QLF). The structures were aligned based on the ferlin core domains (C_2C-C_2D). The C_2B and C_2E-C_2G domains adopt comparable orientations and the overall configuration of the ferlin ring is highly similar.
(D) Protein-lipid interfaces in the nanodisc-bound structures of human myoferlin (1-1997, PDB 9H6X) and mouse otoferlin (216-1931, this work). The ferlin models are colour-coded and fitted into 8 Å (myoferlin) or 6 Å (otoferlin) low-pass filtered maps of the nanodisc-bound complexes. In both structures, the C_2B and C_2F-C_2G domains interact with the lipid nanodisc. However, the C_2C domain and the iDysF motif (absent from otoferlin) provide additional contacts in the myoferlin structure.
(E) Ca^{2+}-sensitive recognition of phosphatidylserine (PS) headgroups in otoferlin, myoferlin, and in other C_2 domain structures. The C_2 domain models are shown in cartoon representation, with bound Ca^{2+} ions depicted as spheres. Aided by the β-hairpin motif of C_2G, the C_2F domain of otoferlin recognizes two PS molecules via three coordinated Ca^{2+} ions, compared with a single PS headgroup in myoferlin's C_2F (PDB 9H6X), synaptotagmin-1 (Syt1) C_2B (PDB 2yoa), or the C_2 domain of PKCα (PDB 1dsy).

Figure S9

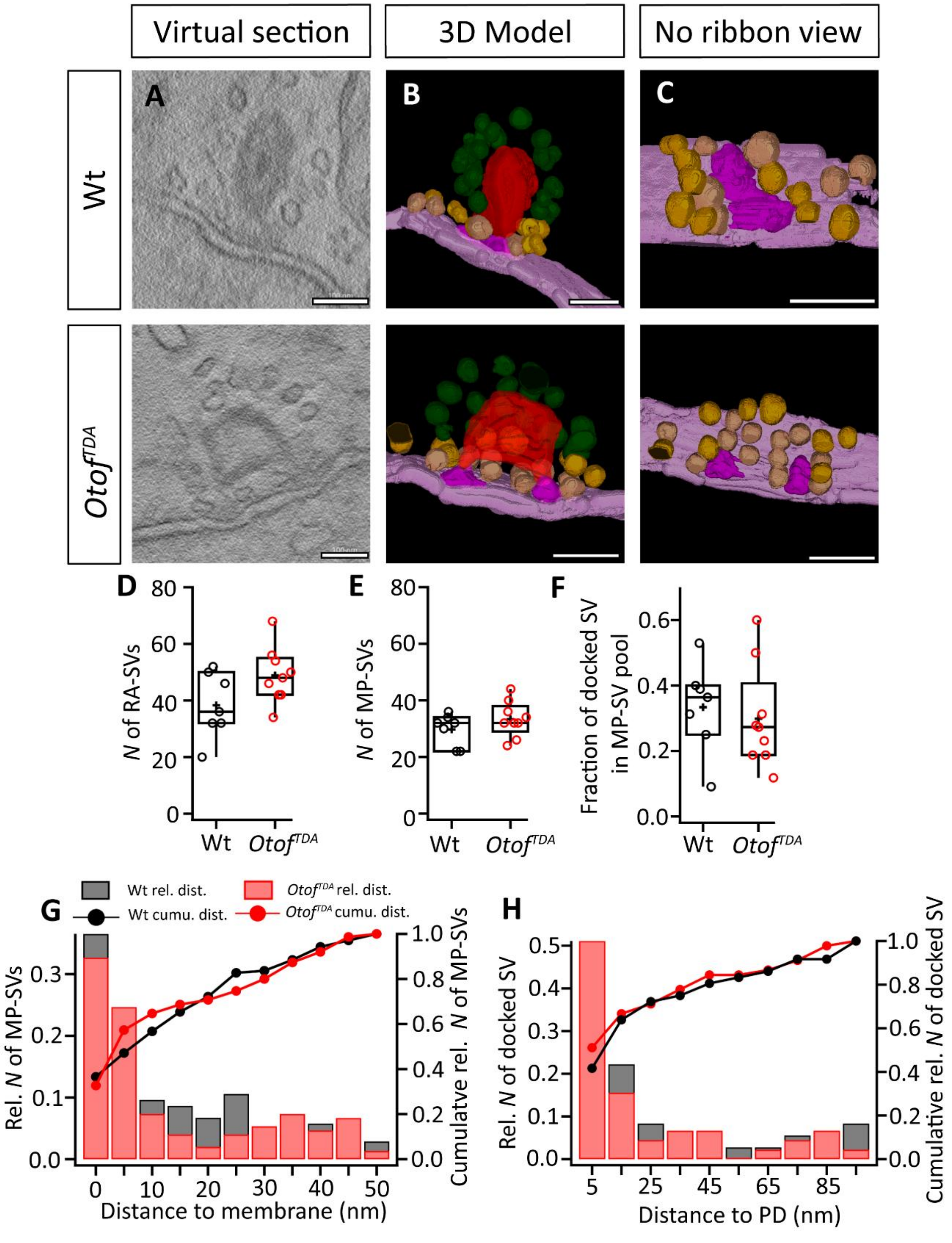

Virtual section
3D Model
No ribbon view
A
B
C
Wt
Otof^TDA
D
E
F
N of RA-SVs
N of MP-SVs
Fraction of docked SV in MP-SV pool
Wt
Otof^TDA
G
H
Wt rel. dist.
Otof^TDA rel. dist.
Wt cumu. dist.
Otof^TDA cumu. dist.
Rel. N of MP-SVs
Cumulatve rel. N of MP-SVs
Distance to membrane (nm)
Rel. N of docked SV
Cumulative rel. N of docked SV
Distance to PD (nm)

Figure S9. Disruption of Ca^{2+}-binding by the C_2F domain (previously C_2E domain) does not change SV number and distribution at ribbon synapses

(A) Representative virtual electron tomogram sections of IHC ribbon synapses from an *Otof*$^{D1563/1565/1570A}$ (*Otof*^{TDA}) animal and an *Otof*^{wt} (here abbreviated Wt) control. Scale bar: 100 nm.

(B) Reconstructed model of the area around the ribbon synapses for the tomograms corresponding to sections from A. The ribbon is rendered in red, the presynaptic density in magenta, and the active zone membrane in soft pink. Vesicles are classified into three pools, ribbon-associated (RA-SV, green), membrane-proximal (MP-SV, orange), and docked (pink). Scale bar: 100 nm.

(C) Top view onto the AZ reconstructions with MP-SVs, ribbon and RA-SVs are not displayed in this perspective. Scale bar: 100 nm.

(D) Number (*N*) of RA-SVs per ribbon synapse. The numbers were not significantly different in IHCs of *Otof*^{TDA} animals compared to Wt ($N_{wt} = 19.1 \pm 2.2$, $n = 7$ ribbons, $N_{animals} = 1$ vs. $N_{mut} = 24.4 \pm 1.6$, $n = 9$ ribbons, $N_{animals} = 2$, Wilcoxon signed-rank test: $p > 0.05$,).

(E) Number (*N*) of MP-SVs per ribbon synapse. The numbers were not significantly different in IHCs of *Otof*^{TDA} animals compared to Wt ($N_{wt} = 14.9 \pm 1.1$, $n = 7$ ribbons vs. $N_{mut} = 16.7 \pm 1.0$, $n = 9$ ribbons, Wilcoxon signed-rank test: $p > 0.05$).

(F) Fraction of docked SVs in the MP-SV pool per ribbon synapse. The fractions were not significantly different in IHCs of *Otof*^{TDA} animals compared to Wt ($Fraction_{wt} = 0.334 \pm 0.051$, $n = 7$ ribbons vs. $Fraction_{mut} = 0.298 \pm 0.052$, $n = 9$ ribbons, Wilcoxon signed-rank test: $p > 0.05$).

(G) Distance of MP-SVs to the active zone membrane, histogram with bins of 3.3 nm for wt (gray) and KO (red) and cumulative distribution (Wt: black, *Otof*^{TDA}: red). The distances were not significantly different in IHCs of *Otof*^{TDA} animals compared to Wt ($distance_{wt} = 13.8 \pm 1.4$ nm, $n = 104$ MP-SVs vs. $distance_{mut} = 13.8 \pm 1.3$ nm, $n = 150$ MP-SVs, Wilcoxon signed-rank test: $p > 0.05$).

(H) Distance of docked SVs to the presynaptic density. It was not significantly different in IHCs of *Otof*^{TDA} animals compared to Wt ($distance_{wt} = 25.4 \pm 5.0$ nm, $n = 36$ docked SVs vs. $distance_{mut} = 22.7 \pm 4.3$ nm, $n = 45$ ribbons, Wilcoxon signed-rank test: $p > 0.05$).

For further information see Table S4.

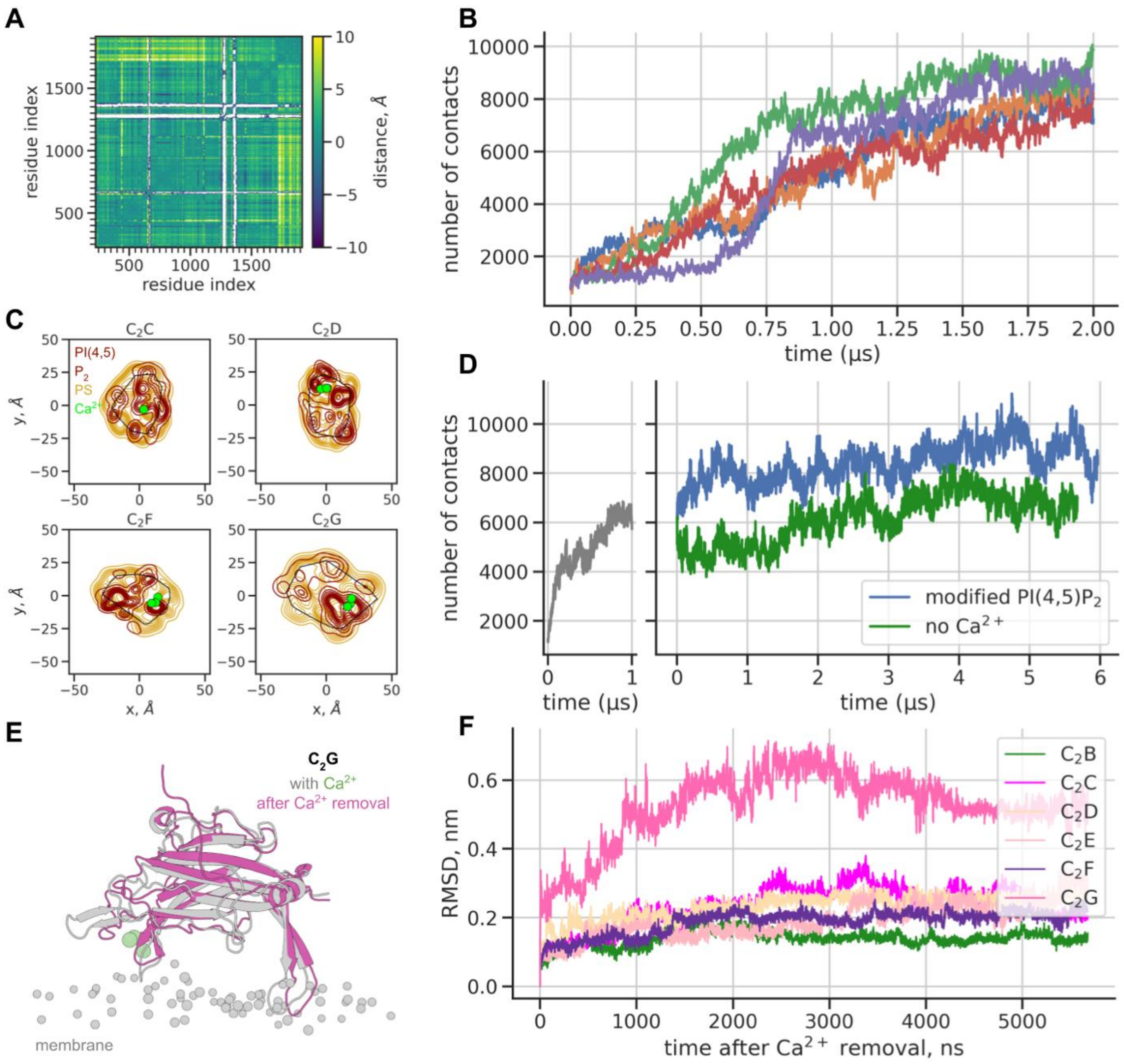

Figure S10. MD simulations provide evidence that electrostatic interactions contribute to the otoferlin lipid binding, but are not the only source of protein-membrane interaction.

(A) Differences between the performed MD simulations and the cryo-EM structure. The matrix shows, color coded, the difference between the average C_α contact matrix calculated for the last 200 ns of the five simulation replicates and the C_α contact matrix calculated for the initial cryo-EM structure. For visualization purposes, all the values below -10 Å or above 10 Å, corresponding to disordered regions of the protein, were masked out.

(B) Increased binding interface. Shown are the time traces of the number of interatomic contacts between otoferlin and membrane in five simulation replicates which started from the cryo-EM structure of lipid-bound otoferlin.

(C) Kernel density estimates for PS (yellow) and $PI(4,5)P_2$ (in red) together with contours of calcium-binding C_2 domains projected onto the membrane plane. Positions of calcium ions are

indicated by green dots. The origin of the plots is located at the projection of the center of mass of every C_2 domain. Densities were scaled by the number of observations for the lipid type separately for every C_2 domain.

(D) Lipid interface of otoferlin during initial binding (starting from an MD-equilibrated structure with a modelled transmembrane domain which was removed prior to system assembly for the simulation run) and in two experimental conditions in MD simulations. The last frame of the simulation of initial binding was used as the starting point for both conditions.

(E) Conformations of the C_2G domain before (gray) and after (magenta) Ca^{2+} removal in the simulations described in D. Coordinates were aligned using only the C_α atoms of the C_2G domain. The three calcium ions bound to C_2G before simulations are indicated in green. The phosphorus atoms of lipid headgroups before Ca^{2+} removal are depicted as spheres. After Ca^{2+} removal, C_2G partly detached from the lipid membrane, which is not displayed for clarity of visualization.

(F) Root mean square deviation (RMSD) traces of C_2 domains after Ca^{2+} removal. Domains were aligned to their starting conformations using the C_α atoms, and the RMSDs of C_α atoms were plotted over simulation time.

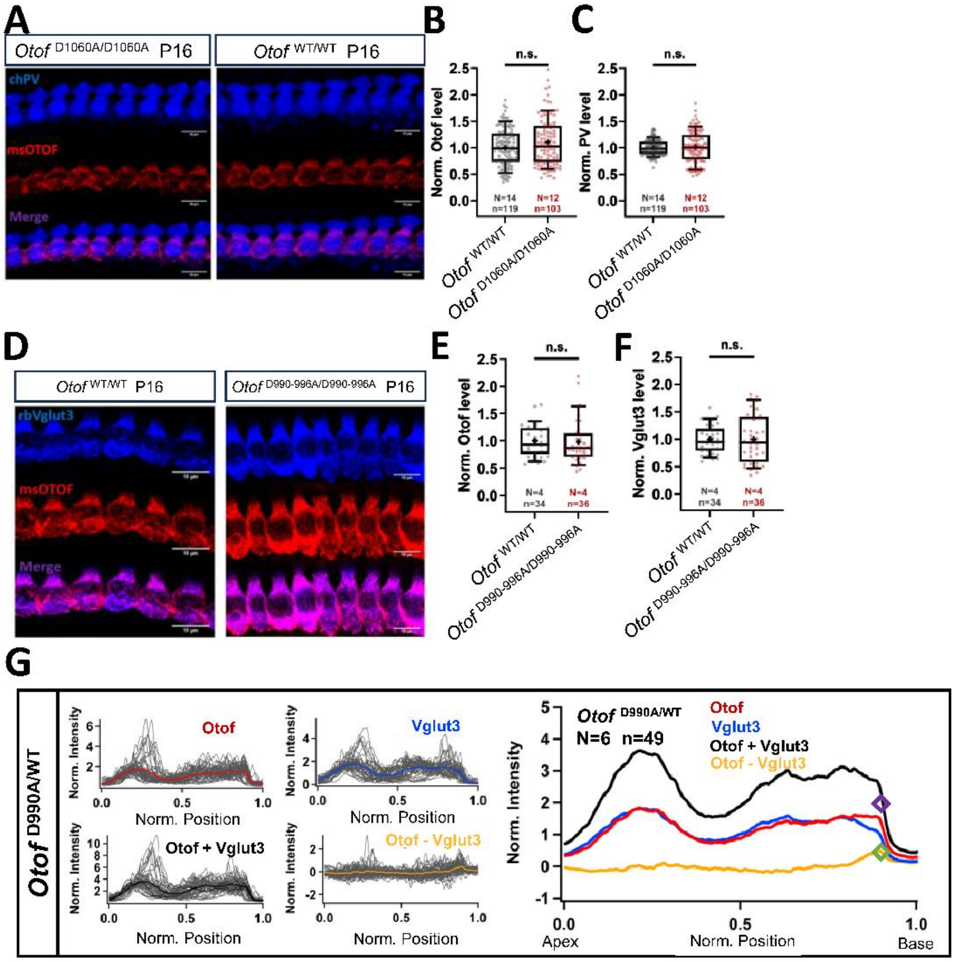

Figure S11. Substantial expression and normal subcellular distribution of otoferlin in otoferlin mutant mice

(A, D) Staining IHCs with otoferlin (OTOF) and parvalbumin (PV) / Vglut3 antibodies. Scale bar, 10 µm.

(B, E) Otoferlin immunofluorescence intensity indicated unchanged otoferlin levels in *Otof*^{D1060A} and *Otof*$^{D990\text{-}996A}$ IHCs compared to littermate control IHCs (Wilcoxon signed-rank test). Box and whisker plots represent median, 25th and 75th as well as 10th and 90th percentiles.

(C, F) Parvalbumin (PV) / Vglut3 immunofluorescence intensity indicated unchanged parvalbumin / Vglut3 levels in otoferlin mutant IHCs compared to littermate control IHCs (Wilcoxon signed-rank test). Box and whisker plots represent median, 25th and 75th as well as 10th and 90th percentiles.

(G) Line profile analysis. The line is indicated as a white dashed line in Fig.7A. The 0 and 1 indicate the start and end point of the line for line scans. For quantification of membrane staining, the fluorescence was normalized to the cellular fluorescence for each fluorophore. The sum of both

fluorescence values (black line) was used to determine the position of the basal membrane. At the most basal cellular point along this line which exceeds the threshold value of 2 (purple diamond), the otoferlin-Vglut3 fluorescence difference (yellow line) gave the value for relative otoferlin plasma membrane levels (green diamond).

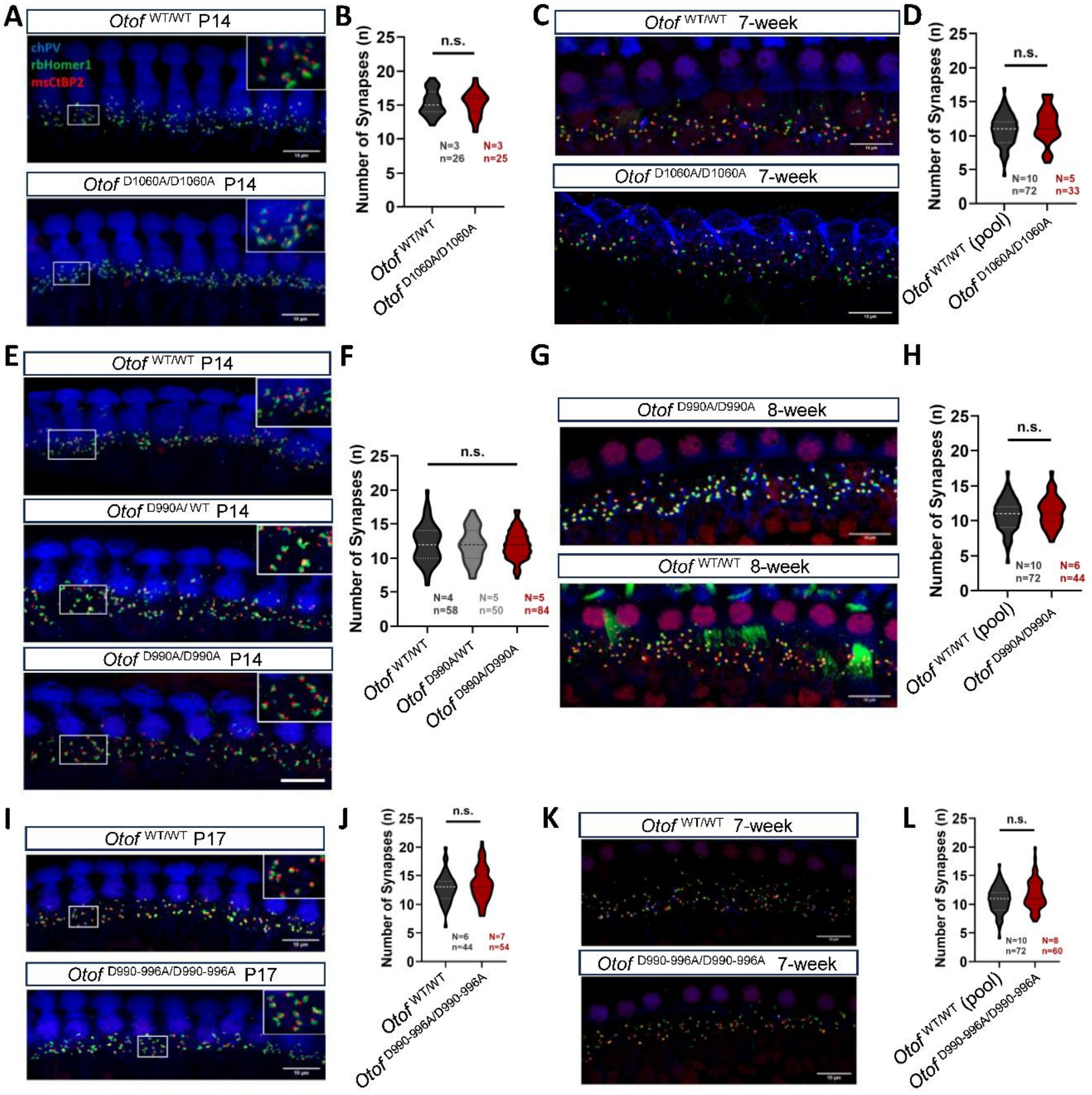

Figure S12. The number of ribbon synapses in otoferlin mutant IHCs remain unchanged

(A, E, I) Maximum-intensity projections of confocal stacks of P 2-week mouse IHCs of different genotypes following immunolabeling for parvalbumin (blue), ribeye (red) and homer-1 (green). Only juxtaposed red and green dots are counted as ribbon synapses. Scale bar, 10 µm.

(B, F, J) Statistics showed that the numbers of ribbon synapses per IHC are similar between otoferlin mutant mice and littermate control mice at 2 weeks of age.

(C, G, K) Maximum-intensity projections of confocal stacks of P 8-week mouse IHCs of different genotypes following immunolabeling for parvalbumin (blue), ribeye (red) and homer-1 (green). Only juxtaposed red and green pairs are counted as ribbon synapses. Scale bar, 10 µm.

(D, H, L) The numbers of ribbon synapses per IHC are similar between otoferlin mutant mice and littermate control mice at 8 weeks of age.

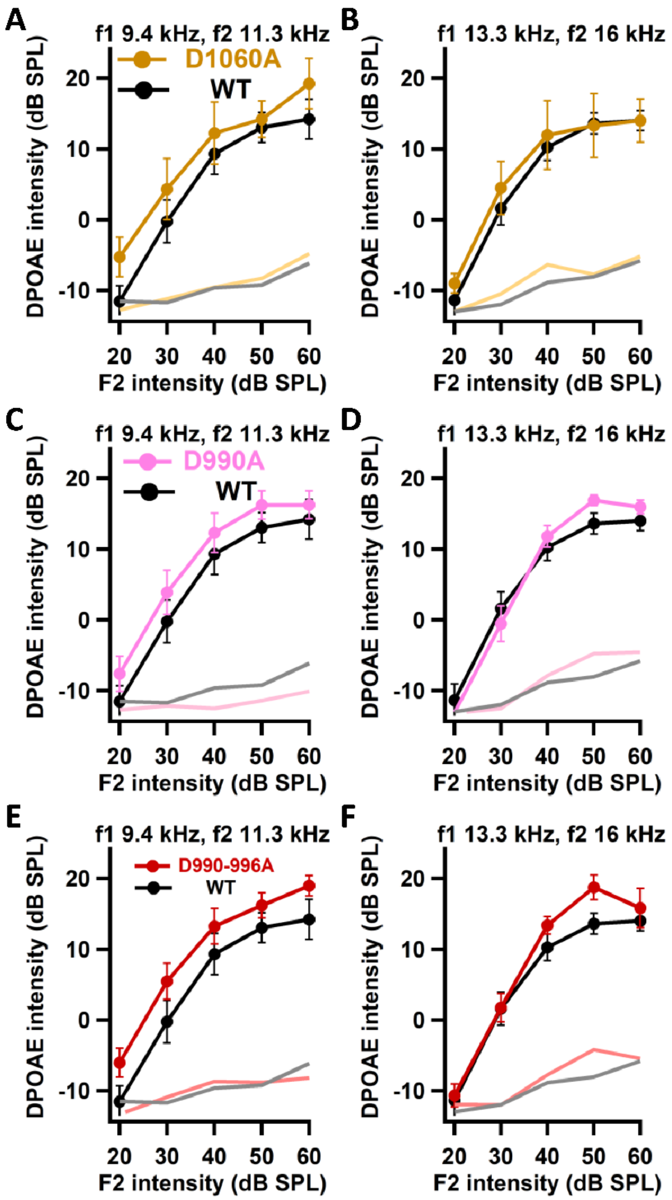

Figure S13. Otoacoustic emissions indicate intact outer hair cell mediated cochlear amplification

(A-B) Robust DPOAE in homozygous *Otof*^{D1060A} mice (yellow, n=7) indicate normal OHC function across the cochlear frequency range. DPOAE growth function for two different pairs of stimulating primary tones: 9.4/11.3 kHz (A) and 13.3/16kHz (B).

C-D) Similarly, homozygous *Otof*^{D990A} mice (magenta, n=8) and homozygous *Otof*$^{D990-996A}$ mice (E-F, red, n=9) have normal DPOAE across all tested frequency/intensity combinations. Data from the same 8 wildtype controls are shown in all panels in black. All data are displayed as mean±SEM. Light yellow/pink/grey lines indicate the average noise floor. No significant differences between mutant and wildtype mice were detected.

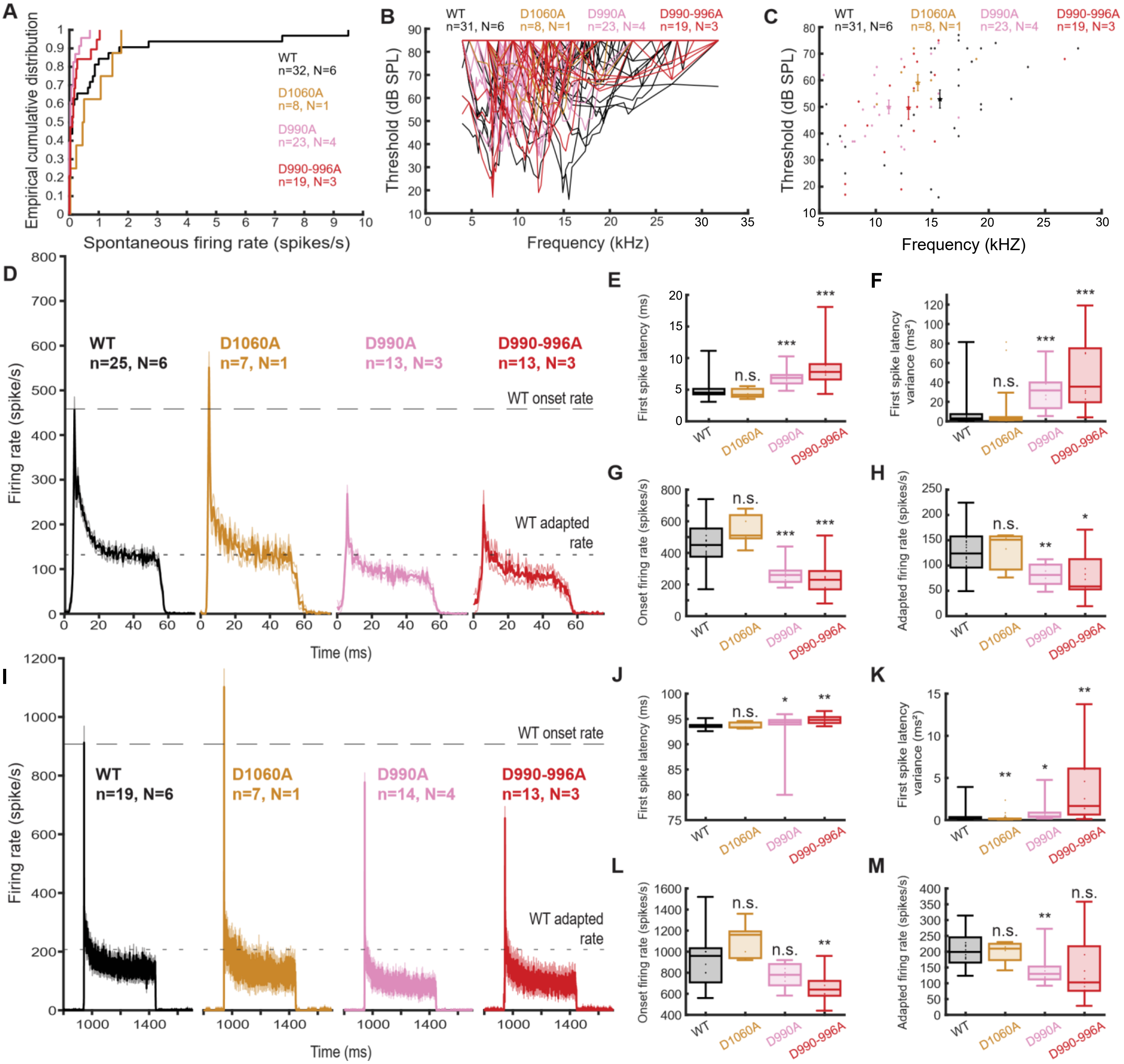

Figure S14. Spontaneous and sound-evoked firing of spiral ganglion neurons indicate a reduced rate of initial release but largely intact vesicle replenishment at the afferent synapse of C$_2$D mutants

(A) Spontaneous firing rates of SGNs for *Otof*^{wt} (black, N = 6, n = 32), homozygous *Otof*^{D1060A} (yellow N = 1, n = 8), *Otof*^{D990A} (magenta, N = 4, n = 24), and *Otof*$^{D990\text{-}996A}$ (red, N = 3, n = 19) mice displayed as an empirical cumulative distribution function.

(B) Tuning curves of SGNs for *Otof*^{wt} (black, N = 6, n = 31), homozygous *Otof*^{D1060A} (yellow N = 1, n = 8), *Otof*^{D990A} (magenta, N = 4, n = 23), and *Otof*$^{D990-996A}$ (red, N = 3, n = 19) mice showing the sound pressure level (dB) at which a response above spontaneous firing rate was elicited for tone bursts of varying frequencies.

(C) Thresholds and characteristic frequencies of the SGNs from the tuning curves in b. Characteristic frequency was defined as the pure tone frequency that elicited a response above spontaneous firing at the lowest sound pressure level (dB). Means thresholds are indicated with stars and error bars represent the standard error of the mean (SEM).

(D) Peristimulus time histograms of SGN firing in response to 50 ms tonebursts at characteristic frequency and 30 dB above threshold with an 100ms interstimulus interval for *Otof*^{wt} (black, N = 6, n = 25), homozygous *Otof*^{D1060A} (yellow N = 1, n = 7), *Otof*^{D990A} (magenta, N = 3, n = 13), and *Otof*$^{D990-996A}$ (red, N = 3, n = 13) mice. Mean traces are plotted with SEM indicated by fainter lines.

(E) Median first spike latency, (F) first spike latency variance, (G) onset firing rate, and (H) adapted firing rate of the SGNs shown in d.

(I) Peristimulus time histograms of SGN firing in response to 50 ms tonebursts at characteristic frequency and 30 dB above threshold with an 2000ms interstimulus interval for *Otof*^{wt} (black, N = 6, n = 19), homozygous *Otof*^{D1060A} (yellow N = 1, n = 7), *Otof*^{D990A} (magenta, N = 4, n = 14), and *Otof*$^{D990-996A}$ (red, N = 3, n = 13) mice. Mean traces are plotted with standard error of the mean indicated by fainter lines.

(J) Median first spike latency, (K) first spike latency variance, (L) onset firing rate, and (M) adapted firing rate of the SGNs shown in d. One variance in first spike latency data point was excluded from the *Otof*^{D990A} set (k) because it was more than 700 IQRs beyond the 3rd quartile. The removal of this data point did not affect the significance level of the statistical analyses. The unit from which this value was recorded was not removed from the dataset as other parameters we recorded were within acceptable ranges.

Data in E-H, and J-M, and P-R are shown as box and whisker plots with data points overlaid, median, 25th and 75th percentiles (box), 10th and 90th percentiles (whiskers) displayed.

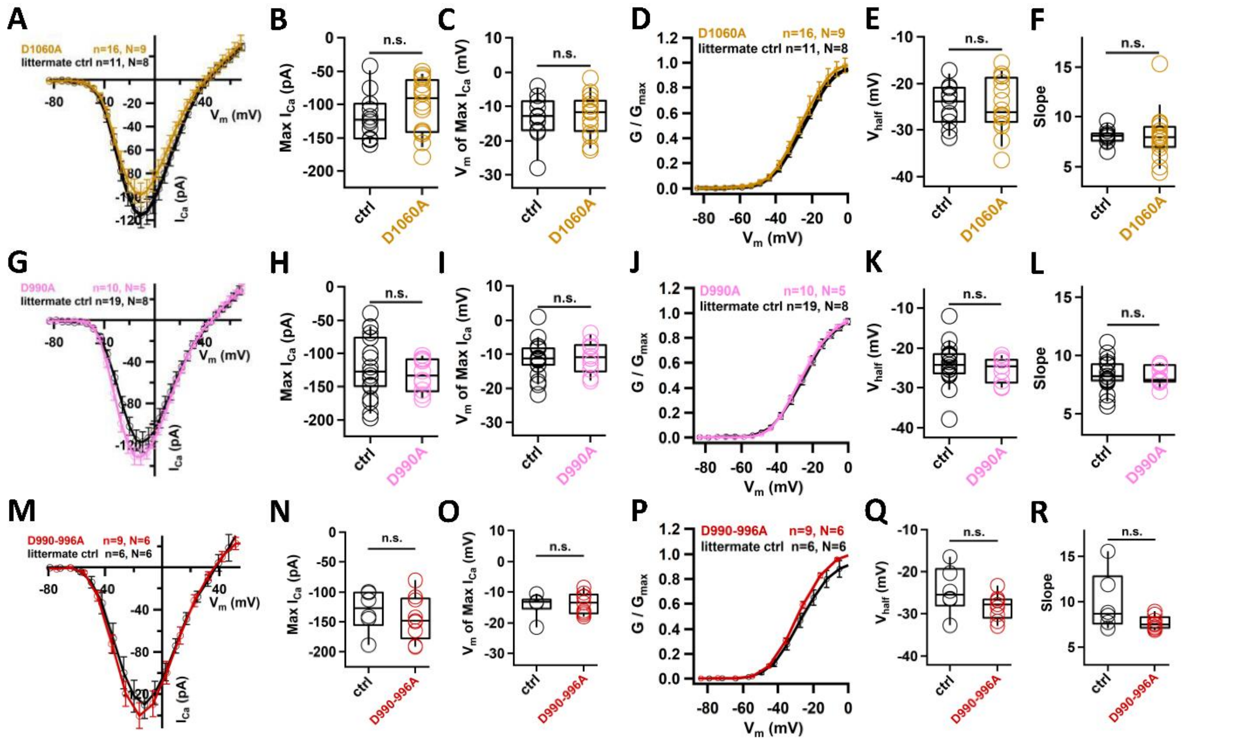

Figure S15. Unchanged amplitude and voltage-dependence of activation of Ca^{2+}-influx in mutant IHCs

(A-C, G-I, M-O) Current-voltage relationships of voltage-dependent Ca^{2+} currents (A, G, M) do not show significant differences in current amplitude (B, H, N) or voltage-dependence (C, I, O).

(D-F, J-L, P-R) Fractional activation of Ca^{2+} channels (D, J, P) extracted from the current-voltage relationships does not show significant difference in the voltage of half-maximal activation (V_{half}, E, K, Q) or voltage-sensitivity (slope, F, L, R).

Table S1. Data collection, refinement and validation statistics for the otoferlin (216-1931)-nanodisc complexes

	Map M1 (30% PS, 10% PI(4,5)P_2) PDB 9QE2, EMD-53046	Map M2 (25% PS, 5% PI(4,5)P_2) PDB 9SEA, EMD-54805	Map M5 (merged datasets) PDB 9SFL, EMD-54827
**Data collection and processing**			
Electron gun	X-FEG	X-FEG	
Detector	Falcon4i	Falcon4i	
Magnification	165,000	165,000	
Energy filter slit width (eV)	10	10	
Voltage (kV)	300.0	300.0	
Dose rate ($e^-/A^2/s$)	15.2	15.8	
Electron exposure on sample (e^-/A^2)	37.85	38.08	
Target defocus range (µm)	1.1-2.3	0.8-2.0	
Calibrated pixel size (Å)	0.72	0.72	
Symmetry imposed	C1	C1	
Collected movies (no.)	12,448	11,324	
Initial particle images (no.)	3,390,520	3,659,311	
Final particle images (no.)	751,004	394,677	
Map resolution at FSC=0.143 (Å)	2.3	2.23	
**Refinement**			
Initial model used	AlphaFold3	otoferlin (216-1931)	otoferlin (216-1931)
Model resolution (Å)	2.5	2.3	2.3
Model resolution at FSC=0.5 (Å)	2.53	2.43	2.32
Map sharpening B factor (Å^2)	-85.7	-69.0	-60.0
Model composition			
Non-hydrogen atoms	12,479	12,380	12,381
Protein residues	1,547	1,533	1,533
Ligands	2x PS	2x PS	2x PS
Waters	-	-	1
Ions	9x Ca^{2+}	9x Ca^{2+}	9x Ca^{2+}
B factors (Å^2)			
Protein (min/max/mean)	52.28/210.98/103.85	57.65/195.91/102.11	35.99/204.73/83.36
Ligand (min/max/mean)	71.89/142.79/100.18	79.82/138.83/107.02	56.33/130.25/92.17
Water	-	-	55.54/55.54/55.54
R.m.s. deviations			
Bond lengths (Å)	0.005	0.004	0.005
Bond angles (°)	0.823	0.795	0.806
**Validation**			
Molprobity score	1.04	1.12	1.01
Clashscore	1.70	1.75	1.47
Rotamer outliers (%)	0.59	0.81	0.66
Cβ outliers (%)	-	-	-
CaBLAM outliers (%)	1.77	1.52	1.52
Ramachandran plot			
Favored (%)	97.4	96.91	97.37
Allowed (%)	2.54	2.96	2.56
Disallowed (%)	0.07	0.13	0.07

Table S2. Data collection, refinement and validation statistics for the lipid-free otoferlin (216-1931) structures

	**Map M6 (class 1)**<br>**Ca^{2+}-bound**<br>**PDB 9SEG, EMD-54809**	**Map M7**<br>**("closed"-like)**<br>**Ca^{2+}-bound**	**Map M9 (class 2)**<br>**Ca^{2+}-bound**<br>**PDB 9SE5, EMD-54802**	**Map M10**<br>**Ca^{2+}-free**<br>**PDB 9SH0, EMD-54883**	
**Data collection and processing**					
Electron gun	X-FEG			X-FEG	X-FEG
Detector	Falcon4i			Falcon4i	Falcon4i
Magnification	165,000			165,000	165,000
Energy filter slit width (eV)	10			10	10
Voltage (kV)	300.0			300.0	300.0
Dose rate ($e^-/A^2/s$)	15.5			15.03	15.41
Electron exposure on sample (e^-/A^2)	38.13			37.88	37.91
Target defocus range (μm)	1.1-2.3			1.1-2.0	1.1-2.3
Calibrated pixel size (Å)	0.72			0.72	0.72
Symmetry imposed	C1			C1	C1
Collected movies (no.)	11,981			3,489	8,187
Initial particle images (no.)	5,201,261			1,211,666	2,222,540
Final particle images (no.)	189,916	66,665	91,966	163,771	
Map resolution at FSC=0.143 (Å)	2.91	3.07	3.41	3.43	
**Refinement**					
Initial model used	otoferlin (216-1931)	otoferlin (216-1931)	otoferlin (216-1931)	otoferlin (216-1931)	
Model resolution (Å)	3.5	3.1	3.6	3.5	
Model resolution at FSC=0.5 (Å)	3.51	4.03	3.7	4.09	
Map sharpening B factor ($Å^2$)	-95.0	-60.7	-75.58	-113.7	
Model composition					
Non-hydrogen atoms	11,977	12,199	11,944	10,297	
Protein residues	1,489	1,517	1,483	1,284	
Ligands	-	-	-	-	
Waters	-	-	-	-	
Ions	7x Ca^{2+}	9x Ca^{2+}	5x Ca^{2+}	-	
B factors ($Å^2$)					
Protein (min/max/mean)	35.86/247.35/112.52	30.35/213.45/103.75	26.06/241.84/102.82	84.0/325.01/156.15	
Ligand (min/max/mean)	62.47/224.90/146.67	97.01/155.05/126.82	63.30/176.17/123.57	-	
Water	-	-	-	-	
R.m.s. deviations					
Bond lengths (Å)	0.005	0.005	0.004	0.004	
Bond angles (°)	0.976	1.060	0.951	1.031	
**Validation**					
Molprobity score	1.67	1.82	1.47	1.59	
Clashscore	3.20	4.55	3.08	2.88	
Rotamer outliers (%)	2.66	2.16	1.60	1.76	
Cβ outliers (%)	-	-	-	0.08	
CaBLAM outliers (%)	1.57	2.81	1.51	2.54	
Ramachandran plot					
Favored (%)	96.47	95.22	96.66	95.36	
Allowed (%)	3.46	4.52	3.20	4.56	
Disallowed (%)	0.07	0.27	0.14	0.08	

Table S3. List of patient mutations, their phenotypes and mapping on the otoferlin structure

Nucleotide change	Amino acid change	Domain	Severity	Zygosity	Refer-ence
c.650A>G	p.D217G	C_2A-C_2B linker	Mild	Comp het (p.A1802V)	(*117*)
c.1469C>A	p.P490Q	C_2C	Profound	Comp het (p.I515T)	(*118*)
c.1544T>C	p.I515T	C_2C	Profound	Comp het (p.P490Q)	(*118*)
c.1550T>C	p.L517P	C_2C	Profound	Comp het (p.I1967del)	(*119*)
c.1841G>A	p.G614E	C_2CD	Mild	Comp het (p.R1080P)	(*120*)
c.3127G>A	p.G1043S	C_2D	Unreported	Hom	--
c.3239G>C	p.R1080P	C_2D	Mild	Comp het (p.G614E)	(*120*)
c.4718T>C	p.I1573T	C_2F	Mild to profound	Hom and Comp het (p. Ala1377Argfs*142)	(*121–123*)
c.4882C>A	p.P1628T	C_2F (dsRBD-like)	Mild	Comp het (p.E1700G)	(*124*)
c.5332G>T	p.V1778F	C_2G	Mild to moderate	Hom	(*125*)
c.5405C>T	p.A1802V	C_2G	Mild	Comp het (p.D217G)	(*117*)
c.5524G>A	p.D1842N	C_2G	Moderate	Het	(*82*)
c.5900_5902delTCA	p.I1967del	TM proximal	Mild to moderate, 15% speech perception	Comp het (p.L517P)	(*119*)
Abbreviations: Hom: homozygous, Comp het: compound heterozygous, Het: heterozygous					

Table S4. Statistical analysis of electron tomography of Otof^{TDA} mice.

***N* of RA-SVs**	
Wt (*n* = 7 ribbons)	19.1 ± 2.2
Otof^{TDA} (*n* = 9 ribbons)	24.4 ± 1.6
p	> 0.05
Mice: N = 1 WT; *N* = 2 Otof^{TDA}	Wilcoxon signed-rank test
***N* of MP-SVs**	
Wt (*n* = 7 ribbons)	14.9 ± 1.1
Otof^{TDA} (*n* = 9 ribbons)	16.7 ± 1.0
p	> 0.05
Mice: N = 1 WT; *N* = 2 Otof^{TDA}	Wilcoxon signed-rank test
**Fraction of docked SV in MP-SV pool**	
Wt (*n* = 7 ribbons)	0.334 ± 0.051
Otof^{TDA} (*n* = 9 ribbons)	0.298 ± 0.052
p	> 0.05
Mice: N = 1 WT; *N* = 2 Otof^{TDA}	Wilcoxon signed-rank test
**Average distance of MP-SVs to the membrane**	
Wt (*n* = 104 MP-SVs)	13.8 ± 1.4
Otof^{TDA} (*n* = 150 MP-SVs)	13.8 ± 1.3
P	> 0.05
Mice: N = 1 WT; *N* = 2 Otof^{TDA}	Wilcoxon signed-rank test
**Average distance of docked SV to the PD**	
Wt (*n* = 36 docked SVs)	25.4 ± 5.0
Otof^{TDA} (*n* = 150 docked SVs)	22.7 ± 4.3
p	> 0.05
Mice: N = 1 WT; *N* = 2 TDA KO	Wilcoxon signed-rank test

Movie Legends

Movie S1. Cryo-EM maps of membrane-bound otoferlin (residues 216-1931).
The final cryo-EM map of otoferlin (residues 216-1931) bound to an MSP2N2 lipid nanodisc has been coloured after the modelled domains. A low-pass filtered (to 8 Å) cryo-EM density map (grey) has been contoured around the high-resolution map of otoferlin.

Movie S2. The membrane-binding interfaces resolved in the otoferlin (216-1931)-nanodisc complex.
The cryo-EM density map of otoferlin (216-1931) has been low-passed to 8 Å to enable the visualization of ordered protein-membrane contacts and is contoured (grey) around the final cryo-EM model. The resolved structural motifs of otoferlin are coloured.

Movie S3. Morphing between the lipid-free and nanodisc-bound structures of otoferlin.
The two structures were superimposed based on the rigid C_2C-C_2D region and the movement of the otoferlin domains between the two models (morphing) were estimated in ChimeraX v.1.8.

Movie S4. Superposition of the lipid-free and nanodisc-bound structures of otoferlin (216-1931).
The two cryo-EM structures of otoferlin were aligned based on the C_2C-C_2D region. The domains of otoferlin are colour-coded as in **Fig. 1**.

REFERENCES AND NOTES

1. T. Moser, C. P. Grabner, F. Schmitz, Sensory processing at ribbon synapses in the retina and the cochlea. *Physiol. Rev.* **100**, 103–144 (2020).

2. M. A. Rutherford, H. Von Gersdorff, J. D. Goutman, Encoding sound in the cochlea: From receptor potential to afferent discharge. *J. Physiol.* **599**, 2527–2557 (2021).

3. S. L. Johnson, S. Safieddine, M. Mustapha, W. Marcotti, Hair cell afferent synapses: Function and dysfunction. *Cold Spring Harb. Perspect. Med.* **9**, a033175 (2019).

4. S. Safieddine, R. J. Wenthold, SNARE complex at the ribbon synapses of cochlear hair cells: Analysis of synaptic vesicle- and synaptic membrane-associated proteins. *Eur. J. Neurosci.* **11**, 803–812 (1999).

5. A. Brandt, J. Striessnig, T. Moser, CaV1.3 channels are essential for development and presynaptic activity of cochlear inner hair cells. *J. Neurosci.* **23**, 10832–10840 (2003).

6. I. Roux, S. Safieddine, R. Nouvian, M. Grati, M.-C. Simmler, A. Bahloul, I. Perfettini, M. Le Gall, P. Rostaing, G. Hamard, A. Triller, P. Avan, T. Moser, C. Petit, Otoferlin, defective in a human deafness form, is essential for exocytosis at the auditory ribbon synapse. *Cell* **127**, 277–289 (2006).

7. R. P. Seal, O. Akil, E. Yi, C. M. Weber, L. Grant, J. Yoo, A. Clause, K. Kandler, J. L. Noebels, E. Glowatzki, L. R. Lustig, R. H. Edwards, Sensorineural deafness and seizures in mice lacking vesicular glutamate transporter 3. *Neuron* **57**, 263–275 (2008).

8. N. Strenzke, S. Chanda, C. Kopp-Scheinpflug, D. Khimich, K. Reim, A. V. Bulankina, A. Neef, F. Wolf, N. Brose, M. A. Xu-Friedman, T. Moser, Complexin-I is required for high-fidelity transmission at the endbulb of held auditory synapse. *J. Neurosci.* **29**, 7991–8004 (2009).

9. R. C. Uthaiah, A. J. Hudspeth, Molecular anatomy of the hair cell's ribbon synapse. *J. Neurosci.* **30**, 12387–12399 (2010).

10. E. Reisinger, C. Bresee, J. Neef, R. Nair, K. Reuter, A. Bulankina, R. Nouvian, M. Koch, J. Bückers, L. Kastrup, I. Roux, C. Petit, S. W. Hell, N. Brose, J.-S. Rhee, S. Kügler, J. V. Brigande, T. Moser, Probing the functional equivalence of otoferlin and synaptotagmin 1 in exocytosis. *J. Neurosci.* **31**, 4886–4895 (2011).

11. R. Nouvian, J. Neef, A. V. Bulankina, E. Reisinger, *T. Pan*gršič, T. Frank, S. Sikorra, N. Brose, T. Binz, T. Moser, Exocytosis at the hair cell ribbon synapse apparently operates without neuronal SNARE proteins. *Nat. Neurosci.* **14**, 411–413 (2011).

12. C. Vogl, B. H. Cooper, J. Neef, S. M. Wojcik, K. Reim, E. Reisinger, N. Brose, J.-S. Rhee, T. Moser, C. Wichmann, Unconventional molecular regulation of synaptic vesicle replenishment in cochlear inner hair cells. *J. Cell Sci.* **128**, 638–644 (2015).

13. A. P. Cepeda, M. Ninov, J. Neef, I. Parfentev, K. Kusch, E. Reisinger, R. Jahn, T. Moser, H. Urlaub, Proteomic analysis reveals the composition of glutamatergic organelles of auditory inner hair cells. *Mol. Cell. Proteomics* **23**, 100704 (2024).

14. J. Rizo, Molecular mechanisms underlying neurotransmitter release.. *Annu. Rev. Biophys.* **51**, 377–408 (2022).

15. R. Jahn, D. C. Cafiso, L. K. Tamm, Mechanisms of SNARE proteins in membrane fusion. *Nat. Rev. Mol. Cell Biol.* **25**, 101–118 (2024).

16. C. Calvet, T. Peineau, N. Benamer, M. Cornille, A. Lelli, B. Plion, G. Lahlou, J. Fanchette, S. Nouaille, J. B. de Monvel, A. Estivalet, P. Jean, V. Michel, M. Sachse, N. Michalski, P. Avan, C. Petit, D. Dulon, S. Safieddine, The SNARE protein SNAP-25 is required for normal exocytosis at auditory hair cell ribbon synapses. *iScience* **25**, 105628 (2022).

17. T. Moser, A. Starr, Auditory neuropathy—Neural and synaptic mechanisms. *Nat. Rev. Neurol.* **12**, 135–149 (2016).

18. S. Yasunaga, M. Grati, M. Cohen-Salmon, A. El-Amraoui, M. Mustapha, N. Salem, E. El-Zir, J. Loiselet, C. Petit, A mutation in OTOF, encoding otoferlin, a FER-1-like protein, causes DFNB9, a nonsyndromic form of deafness. *Nat. Genet.* **21**, 363–369 (1999).

19. S. M. Baig, A. Koschak, A. Lieb, M. Gebhart, C. Dafinger, G. Nürnberg, *A. Ali*, I. Ahmad, M. J. Sinnegger-Brauns, N. Brandt, J. Engel, M. E. Mangoni, M. Farooq, H. U. Khan, P. Nürnberg, J. Striessnig, H. J. Bolz, Loss of Ca(v)1.3 (CACNA1D) function in a human channelopathy with bradycardia and congenital deafness. *Nat. Neurosci.* **14**, 77–84 (2011).

20. I. Schrauwen, S. Helfmann, A. Inagaki, F. Predoehl, M. A. Tabatabaiefar, M. M. Picher, M. Sommen, C. Z. Seco, J. Oostrik, H. Kremer, A. Dheedene, C. Claes, E. Fransen, M. H. Chaleshtori, P. Coucke, A. Lee, T. Moser, G. Van Camp, A mutation in CABP2, expressed in cochlear hair cells, causes autosomal-recessive hearing impairment. *Am. J. Hum. Genet.* **91**, 636–645 (2012).

21. J. Platzer, J. Engel, A. Schrott-Fischer, K. Stephan, S. Bova, H. Chen, H. Zheng, J. Striessnig, Congenital deafness and sinoatrial node dysfunction in mice lacking class D L-type Ca^{2+} channels. *Cell* **102**, 89–97 (2000).

22. A. Brandt, D. Khimich, T. Moser, Few CaV1.3 channels regulate the exocytosis of a synaptic vesicle at the hair cell ribbon synapse. *J. Neurosci.* **25**, 11577–11585 (2005).

23. *T. Pan*gršič, M. Gabrielaitis, S. Michanski, B. Schwaller, F. Wolf, N. Strenzke, T. Moser, EF-hand protein Ca^{2+} buffers regulate Ca^{2+} influx and exocytosis in sensory hair cells. *Proc. Natl. Acad. Sci. U.S.A.* **112**, E1028–E1037 (2015).

24. L. M. Jaime Tobón, T. Moser, Ca^{2+} regulation of glutamate release from inner hair cells of hearing mice. *Proc. Natl. Acad. Sci. U.S.A.* **120**, e2311539120 (2023).

25. *T. Pan*grsic, E. Reisinger, T. Moser, Otoferlin: A multi-C2 domain protein essential for hearing. *Trends Neurosci.* **35**, 671–680 (2012).

26. S. T. Cooper, P. L. McNeil, Membrane repair: Mechanisms and pathophysiology. *Physiol. Rev.* **95**, 1205–1240 (2015).

27. T. Pangrsic, L. Lasarow, K. Reuter, H. Takago, M. Schwander, D. Riedel, T. Frank, L. M. Tarantino, J. S. Bailey, N. Strenzke, N. Brose, U. Müller, E. Reisinger, T. Moser, Hearing

requires otoferlin-dependent efficient replenishment of synaptic vesicles in hair cells. *Nat. Neurosci.* **13**, 869–876 (2010).

28. N. H. Revelo, D. Kamin, S. Truckenbrodt, A. B. Wong, K. Reuter-Jessen, E. Reisinger, T. Moser, S. O. Rizzoli, A new probe for super-resolution imaging of membranes elucidates trafficking pathways. *J. Cell Biol.* **205**, 591–606 (2014).

29. S. Jung, T. Maritzen, C. Wichmann, Z. Jing, A. Neef, N. H. Revelo, H. Al-Moyed, S. Meese, S. M. Wojcik, I. Panou, H. Bulut, P. Schu, R. Ficner, E. Reisinger, S. O. Rizzoli, J. Neef, N. Strenzke, V. Haucke, T. Moser, Disruption of adaptor protein 2μ (AP-2μ) in cochlear hair cells impairs vesicle reloading of synaptic release sites and hearing. *EMBO J.* **34**, 2686–2702 (2015).

30. C. P. Johnson, E. R. Chapman, Otoferlin is a calcium sensor that directly regulates SNARE-mediated membrane fusion. *J. Cell Biol.* **191**, 187–197 (2010).

31. N. Michalski, J. D. Goutman, S. M. Auclair, J. Boutet de Monvel, M. Tertrais, A. Emptoz, A. Parrin, S. Nouaille, M. Guillon, M. Sachse, D. Ciric, A. Bahloul, J.-P. Hardelin, R. B. Sutton, P. Avan, S. S. Krishnakumar, J. E. Rothman, D. Dulon, S. Safieddine, C. Petit, Otoferlin acts as a Ca^{2+} sensor for vesicle fusion and vesicle pool replenishment at auditory hair cell ribbon synapses. *eLife* **6**, e31013 (2017).

32. R. Fernández-Chacón, A. Königstorfer, S. H. Gerber, J. García, M. F. Matos, C. F. Stevens, N. Brose, J. Rizo, C. Rosenmund, T. C. Südhof, Synaptotagmin I functions as a calcium regulator of release probability. *Nature* **410**, 41–49 (2001).

33. O. Kochubey, R. Schneggenburger, Synaptotagmin increases the dynamic range of synapses by driving Ca^{2+}–evoked release and by clamping a near-linear remaining Ca^{2+} sensor. *Neuron* **69**, 736–748 (2011).

34. H. Chen, M. Monga, Q. Fang, L. Slitin, J. Neef, S. S. Chepurwar, R. C. M. Netto, K. Lezirovitz, A. Tabith, F. Benseler, N. Brose, K. Kusch, C. Wichmann, N. Strenzke, B. Vona, J. Preobraschenski, T. Moser, Ca^{2+} binding to the C_2E domain of otoferlin is required for hair cell exocytosis and hearing. *Protein Cell* **15**, 305–312 (2024).

35. D. Beutner, T. Voets, E. Neher, T. Moser, Calcium dependence of exocytosis and endocytosis at the cochlear inner hair cell afferent synapse. *Neuron* **29**, 681–690 (2001).

36. C. Vogl, I. Panou, G. Yamanbaeva, C. Wichmann, S. J. Mangosing, F. Vilardi, A. A. Indzhykulian, *T. Pan*gršič, R. Santarelli, M. Rodriguez-Ballesteros, T. Weber, S. Jung, E. Cardenas, X. Wu, S. M. Wojcik, K. Y. Kwan, I. Del Castillo, B. Schwappach, N. Strenzke, D. P. Corey, S.-Y. Lin, T. Moser, Tryptophan-rich basic protein (WRB) mediates insertion of the tail-anchored protein otoferlin and is required for hair cell exocytosis and hearing. *EMBO J.* **35**, 2536–2552 (2016).

37. N. Strenzke, R. Chakrabarti, H. Al-Moyed, A. Müller, G. Hoch, *T. Pan*grsic, G. Yamanbaeva, C. Lenz, K.-T. Pan, E. Auge, R. Geiss-Friedlander, H. Urlaub, N. Brose, C. Wichmann, E. Reisinger, Hair cell synaptic dysfunction, auditory fatigue and thermal sensitivity in otoferlin Ile515Thr mutants. *EMBO J.* **35**, 2519–2535 (2016).

38. S. V. Duncker, C. Franz, S. Kuhn, U. Schulte, D. Campanelli, N. Brandt, B. Hirt, B. Fakler, N. Blin, P. Ruth, J. Engel, W. Marcotti, U. Zimmermann, M. Knipper, Otoferlin couples to clathrin-mediated endocytosis in mature cochlear inner hair cells. *J. Neurosci.* **33**, 9508–9519 (2013).

39. M. Tertrais, Y. Bouleau, A. Emptoz, S. Belleudy, R. B. Sutton, C. Petit, S. Safieddine, D. Dulon, Viral transfer of mini-otoferlins partially restores the fast component of exocytosis and uncovers ultrafast endocytosis in auditory hair cells of otoferlin knock-out mice. *J. Neurosci.* **39**, 3394–3411 (2019).

40. N. Hams, M. Padmanarayana, W. Qiu, C. P. Johnson, Otoferlin is a multivalent calcium-sensitive scaffold linking SNAREs and calcium channels. *Proc. Natl. Acad. Sci. U.S.A.* **114**, 8023–8028 (2017).

41. S. Helfmann, P. Neumann, K. Tittmann, T. Moser, R. Ficner, E. Reisinger, The crystal structure of the C_2A domain of otoferlin reveals an unconventional top loop region. *J. Mol. Biol.* **406**, 479–490 (2011).

42. A. H. Shaib, A. A. Chouaib, R. Chowdhury, J. Altendorf, D. Mihaylov, C. Zhang, D. Krah, V. Imani, R. K. W. Spencer, S. V. Georgiev, N. Mougios, M. Monga, S. Reshetniak, T. Mimoso, H. Chen, P. Fatehbasharzad, D. Crzan, K.-A. Saal, M. M. Alawieh, N. Alawar, J. Eilts, J. Kang, A. Soleimani, M. Müller, C. Pape, L. Alvarez, C. Trenkwalder, B. Mollenhauer, T. F. Outeiro, S. Köster, J. Preobraschenski, U. Becherer, T. Moser, E. S. Boyden, A. R. Aricescu, M. Sauer, F. Opazo, S. O. Rizzoli, One-step nanoscale expansion microscopy reveals individual protein shapes. *Nat. Biotechnol.*, **43**, 1539–1547 (2025).

43. H.-L. Huang, G. Grandinetti, S. M. Heissler, K. Chinthalapudi, Cryo-EM structures of the membrane repair protein dysferlin. *Nat. Commun.* **15**, 9650 (2024).

44. C. Cretu, A. Chernev, C. Z. Kibédi Szabó, V. Pena, H. Urlaub, T. Moser, J. Preobraschenski, Structural insights into lipid membrane binding by human ferlins. *EMBO J.* **44**, 3926–3958 (2025).

45. B. Vona, A. Rad, E. Reisinger, The many faces of DFNB9: Relating OTOF variants to hearing impairment. *Genes* **11**, 1411 (2020).

46. H. Al-Moyed, A. P. Cepeda, S. Jung, T. Moser, S. Kügler, E. Reisinger, A dual-AAV approach restores fast exocytosis and partially rescues auditory function in deaf otoferlin knock-out mice. *EMBO Mol. Med.* **11**, e9396 (2019).

47. O. Akil, F. Dyka, C. Calvet, A. Emptoz, G. Lahlou, S. Nouaille, J. B. de Monvel, J.-P. Hardelin, W. W. Hauswirth, P. Avan, C. Petit, S. Safieddine, L. R. Lustig, Dual AAV-mediated gene therapy restores hearing in a DFNB9 mouse model. *Proc. Natl. Acad. Sci. U.S.A.* **116**, 4496–4501 (2019).

48. J. Lv, H. Wang, X. Cheng, Y. Chen, D. Wang, L. Zhang, Q. Cao, H. Tang, S. Hu, K. Gao, M. Xun, J. Wang, Z. Wang, B. Zhu, C. Cui, Z. Gao, L. Guo, S. Yu, L. Jiang, Y. Yin, J. Zhang, B. Chen, W. Wang, R. Chai, Z.-Y. Chen, H. Li, Y. Shu, AAV1-hOTOF gene therapy for autosomal recessive deafness 9: A single-arm trial. *Lancet* **403**, 2317–2325 (2024).

49. J. Qi, F. Tan, L. Zhang, L. Lu, S. Zhang, Y. Zhai, Y. Lu, X. Qian, W. Dong, Y. Zhou, Z. Zhang, X. Yang, L. Jiang, C. Yu, J. Liu, T. Chen, L. Wu, C. Tan, S. Sun, H. Song, Y. Shu, L. Xu, X. Gao, H. Li, R. Chai, AAV-mediated gene therapy restores hearing in patients with DFNB9 deafness. *Adv. Sci.* **11**, e2306788 (2024).

50. H. Wang, Y. Chen, J. Lv, X. Cheng, Q. Cao, D. Wang, L. Zhang, B. Zhu, M. Shen, C. Xu, M. Xun, Z. Wang, H. Tang, S. Hu, C. Cui, L. Jiang, Y. Yin, L. Guo, Y. Zhou, L. Han, Z. Gao, J. Zhang, S. Yu, K. Gao, J. Wang, B. Chen, W. Wang, Z.-Y. Chen, H. Li, Y. Shu, Bilateral gene therapy in children with autosomal recessive deafness 9: Single-arm trial results. *Nat. Med.* **30**, 1898–1904 (2024).

51. H. Liu, H. Liu, L. Wang, *L. Song*, G. Jiang, Q. Lu, T. Yang, H. Peng, R. Cai, X. Zhao, T. Zhao, H. Wu, Cochlear transcript diversity and its role in auditory functions implied by an otoferlin short isoform. *Nat. Commun.* **14**, 3085 (2023).

52. K. S Cannon, R. D. Sarsam, T. Tedamrongwanish, K. Zhang, R. W. Baker, Lipid nanodiscs as a template for high-resolution cryo-EM structures of peripheral membrane proteins. *J. Struct. Biol.* **215**, 107989 (2023).

53. H. Chen, Q. Fang, F. Benseler, N. Brose, T. Moser, Probing the role of the C_2F domain of otoferlin. *Front. Mol. Neurosci.* **16**, 1299509 (2023).

54. M. Padmanarayana, N. Hams, L. C. Speight, E. J. Petersson, R. A. Mehl, C. P. Johnson, Characterization of the lipid binding properties of otoferlin reveals specific interactions between PI(4,5)P2 and the C2C and C2F domains. *Biochemistry* **53**, 5023–5033 (2014).

55. N. J. Marty, C. L. Holman, N. Abdullah, C. P. Johnson, The C2 domains of otoferlin, dysferlin, and myoferlin alter the packing of lipid bilayers. *Biochemistry* **52**, 5585–5592 (2013).

56. E. Neher, N. Brose, Dynamically primed synaptic vesicle states: Key to understand synaptic short-term plasticity. *Neuron* **100**, 1283–1291 (2018).

57. T. Moser, D. Beutner, Kinetics of exocytosis and endocytosis at the cochlear inner hair cell afferent synapse of the mouse. *Proc. Natl. Acad. Sci. U.S.A.* **97**, 883–888 (2000).

58. J. D. Goutman, E. Glowatzki, Time course and calcium dependence of transmitter release at a single ribbon synapse. *Proc. Natl. Acad. Sci. U.S.A.* **104**, 16341–16346 (2007).

59. L. M. Jaime Tobón, T. Moser, Bridging the gap between presynaptic hair cell function and neural sound encoding. *eLife* **12**, RP93749 (2024).

60. D. Bansal, K. Miyake, S. S. Vogel, S. Groh, C.-C. Chen, R. Williamson, P. L. McNeil, K. P. Campbell, Defective membrane repair in dysferlin-deficient muscular dystrophy. *Nature* **423**, 168–172 (2003).

61. S. Jo, T. Kim, V. G. Iyer, W. Im, CHARMM-GUI: A web-based graphical user interface for CHARMM. *J. Comput. Chem.* **29**, 1859–1865 (2008).

62. J. Ruel, S. Emery, R. Nouvian, T. Bersot, B. Amilhon, J. M. Van Rybroek, G. Rebillard, M. Lenoir, M. Eybalin, B. Delprat, T. A. Sivakumaran, B. Giros, S. El Mestikawy, T. Moser, R. J. H. Smith, M. M. Lesperance, J.-L. Puel, Impairment of SLC17A8 encoding vesicular glutamate transporter-3, VGLUT3, underlies nonsyndromic deafness DFNA25 and inner hair cell dysfunction in null mice. *Am. J. Hum. Genet.* **83**, 278–292 (2008).

63. D. Khimich, R. Nouvian, R. Pujol, S. T. Dieck, A. Egner, E. D. Gundelfinger, T. Moser, Hair cell synaptic ribbons are essential for synchronous auditory signalling. *Nature* **434**, 889–894 (2005).

64. T. Moser, "Presynaptic physiology of cochlear inner hair cells," in *The Senses: A Comprehensive Reference* (Elsevier, 2020), pp. 441–467; https://linkinghub.elsevier.com/retrieve/pii/B9780128093245241850.

65. Y. Hua, X. Ding, H. Wang, F. Wang, Y. Lu, J. Neef, Y. Gao, T. Moser, H. Wu, Electron microscopic reconstruction of neural circuitry in the cochlea. *Cell Rep.* **34**, 108551 (2021).

66. P. Heil, H. Neubauer, D. R. F. Irvine, M. Brown, Spontaneous activity of auditory-nerve fibers: insights into stochastic processes at ribbon synapses. *J. Neurosci.* **27**, 8457–8474 (2007).

67. B. N. Buran, N. Strenzke, A. Neef, E. D. Gundelfinger, T. Moser, M. C. Liberman, Onset coding is degraded in auditory nerve fibers from mutant mice lacking synaptic ribbons. *J. Neurosci.* **30**, 7587–7597 (2010).

68. M. Avissar, J. H. Wittig, J. C. Saunders, T. D. Parsons, Refractoriness enhances temporal coding by auditory nerve fibers. *J. Neurosci.* **33**, 7681–7690 (2013).

69. T. Frank, M. A. Rutherford, N. Strenzke, A. Neef, *T. Pan*gršič, D. Khimich, A. Fejtova, E. D. Gundelfinger, M. C. Liberman, B. Harke, K. E. Bryan, A. Lee, A. Egner, D. Riedel, T. Moser, Bassoon and the synaptic ribbon organize Ca^{2+} channels and vesicles to add release sites and promote refilling. *Neuron* **68**, 724–738 (2010).

70. A. Neef, D. Khimich, P. Pirih, D. Riedel, F. Wolf, T. Moser, Probing the mechanism of exocytosis at the hair cell ribbon synapse. *J. Neurosci.* **27**, 12933–12944 (2007).

71. C. P. Grabner, T. Moser, Individual synaptic vesicles mediate stimulated exocytosis from cochlear inner hair cells. *Proc. Natl. Acad. Sci. U.S.A.* **115**, 12811–12816 (2018).

72. B. D. Winegar, J. B. Lansman, Voltage-dependent block by zinc of single calcium channels in mouse myotubes. *J. Physiol.* **425**, 563–578 (1990).

73. M. Beurg, N. Michalski, S. Safieddine, Y. Bouleau, R. Schneggenburger, E. R. Chapman, C. Petit, D. Dulon, Control of exocytosis by synaptotagmins and otoferlin in auditory hair cells. *J. Neurosci.* **30**, 13281–13290 (2010).

74. Y. Lai, U. B. Choi, J. Leitz, H. J. Rhee, C. Lee, B. Altas, M. Zhao, R. A. Pfuetzner, A. L. Wang, N. Brose, J. S. Rhee, A. T. Brunger, Molecular mechanisms of synaptic vesicle priming by Munc13 and Munc18. *Neuron* **95**, 591–607.e10 (2017).

75. B. Quade, M. Camacho, X. Zhao, M. Orlando, T. Trimbuch, J. Xu, W. Li, D. Nicastro, C. Rosenmund, J. Rizo, Membrane bridging by Munc13–1 is crucial for neurotransmitter release. *eLife* **8**, e42806 (2019).

76. M. Verhage, A. S. Maia, J. J. Plomp, A. B. Brussaard, J. H. Heeroma, H. Vermeer, R. F. Toonen, R. E. Hammer, T. K. van den Berg, M. Missler, H. J. Geuze, T. C. Südhof, Synaptic assembly of the brain in the absence of neurotransmitter secretion. *Science* **287**, 864–869 (2000).

77. F. Varoqueaux, A. Sigler, J.-S. Rhee, N. Brose, C. Enk, K. Reim, C. Rosenmund, Total arrest of spontaneous and evoked synaptic transmission but normal synaptogenesis in the absence of Munc13-mediated vesicle priming. *Proc. Natl. Acad. Sci. U.S.A.* **99**, 9037–9042 (2002).

78. A. T. Brunger, J. Leitz, The core complex of the Ca^{2+}−triggered presynaptic fusion machinery. *J. Mol. Biol.* **435**, 167853 (2023).

79. Q. Zhou, P. Zhou, A. L. Wang, D. Wu, M. Zhao, T. C. Südhof, A. T. Brunger, The primed SNARE-complexin-synaptotagmin complex for neuronal exocytosis. *Nature* **548**, 420–425 (2017).

80. M. Schwander, A. Sczaniecka, N. Grillet, J. S. Bailey, M. Avenarius, H. Najmabadi, B. M. Steffy, G. C. Federe, E. A. Lagler, R. Banan, R. Hice, L. Grabowski-Boase, E. M. Keithley, A. F. Ryan, G. D. Housley, T. Wiltshire, R. J. H. Smith, L. M. Tarantino, U. Müller, A forward genetics screen in mice identifies recessive deafness traits and reveals that pejvakin is essential for outer hair cell function. *J. Neurosci.* **27**, 2163–2175 (2007).

81. R. Chakrabarti, S. Michanski, C. Wichmann, Vesicle sub-pool organization at inner hair cell ribbon synapses. *EMBO Rep.* **19**, e44937 (2018).

82. T. Matsunaga, H. Mutai, S. Kunishima, K. Namba, N. Morimoto, Y. Shinjo, Y. Arimoto, Y. Kataoka, T. Shintani, N. Morita, T. Sugiuchi, S. Masuda, A. Nakano, H. Taiji, K. Kaga, A prevalent founder mutation and genotype-phenotype correlations of OTOF in Japanese patients with auditory neuropathy. *Clin. Genet.* **82**, 425–432 (2012).

83. N. Karagulyan, A. Thirumalai, S. Michanski, Y. Qi, Q. Fang, H. Wang, N. J. Ortner, J. Striessnig, N. Strenzke, C. Wichmann, Y. Hua, T. Moser, Gating of hair cell Ca^{2+} channels governs the activity of cochlear neurons. *Sci. Adv.* **11**, eadu7898 (2025).

84. J. Schmitzová, C. Cretu, C. Dienemann, H. Urlaub, V. Pena, Structural basis of catalytic activation in human splicing. *Nature* **617**, 842–850 (2023).

85. C. Cretu, A. A. Agrawal, A. Cook, C. L. Will, P. Fekkes, P. G. Smith, R. Lührmann, N. Larsen, S. Buonamici, V. Pena, Structural basis of splicing modulation by antitumor macrolide compounds. *Mol. Cell* **70**, 265–273.e8 (2018).

86. C. Cretu, P. Gee, X. Liu, A. Agrawal, T.-V. Nguyen, A. K. Ghosh, A. Cook, M. Jurica, N. A. Larsen, V. Pena, Structural basis of intron selection by U2 snRNP in the presence of covalent inhibitors. *Nat. Commun.* **12**, 4491 (2021).

87. C. Cretu, J. Schmitzová, A. Ponce-Salvatierra, O. Dybkov, E. I. De Laurentiis, K. Sharma, C. L. Will, H. Urlaub, R. Lührmann, V. Pena, Molecular architecture of SF3b and structural consequences of its cancer-related mutations. *Mol. Cell* **64**, 307–319 (2016).

88. D. S. Brandt, M. D. Coffman, J. J. Falke, J. D. Knight, Hydrophobic contributions to the membrane docking of Synaptotagmin 7 C2A domain: Mechanistic contrast between isoforms 1 and 7. *Biochemistry* **51**, 7654–7664 (2012).

89. D. Kimanius, K. Jamali, M. E. Wilkinson, S. Lövestam, V. Velazhahan, T. Nakane, S. H. W. Scheres, Data-driven regularization lowers the size barrier of cryo-EM structure determination. *Nat Methods* **21**, 1216–1221 (2024).

90. A. J. Jakobi, M. Wilmanns, C. Sachse, Model-based local density sharpening of cryo-EM maps. *eLife* **6**, e27131 (2017).

91. J. Jumper, R. Evans, A. Pritzel, T. Green, M. Figurnov, O. Ronneberger, K. Tunyasuvunakool, R. Bates, A. Žídek, A. Potapenko, A. Bridgland, C. Meyer, S. A. A. Kohl, A. J. Ballard, A. Cowie, B. Romera-Paredes, S. Nikolov, R. Jain, J. Adler, T. Back, S. Petersen, D. Reiman, E. Clancy, M. Zielinski, M. Steinegger, M. Pacholska, T. Berghammer, S. Bodenstein, D. Silver, O. Vinyals, A. W. Senior, K. Kavukcuoglu, *P. Kohli*, D. Hassabis, Highly accurate protein structure prediction with AlphaFold. *Nature* **596**, 583–589 (2021).

92. T. I. Croll, ISOLDE: A physically realistic environment for model building into low-resolution electron-density maps. *Acta Crystallogr. D Struct. Biol.* **74**, 519–530 (2018).

93. P. Emsley, K. Cowtan, Coot: Model-building tools for molecular graphics. *Acta Crystallogr. D Biol. Crystallogr.* **60**, 2126–2132 (2004).

94. P. V. Afonine, B. K. Poon, R. J. Read, O. V. Sobolev, T. C. Terwilliger, A. Urzhumtsev, P. D. Adams, Real-space refinement in PHENIX for cryo-EM and crystallography. *Acta Crystallogr. D Struct. Biol.* **74**, 531–544 (2018).

95. R. D. Oeffner, T. I. Croll, C. Millán, B. K. Poon, C. J. Schlicksup, R. J. Read, T. C. Terwilliger, Putting AlphaFold models to work with phenix.process_predicted_model and ISOLDE. *Acta Crystallogr. D Struct. Biol.* **78**, 1303–1314 (2022).

96. K. Yamashita, C. M. Palmer, T. Burnley, G. N. Murshudov, Cryo-EM single-particle structure refinement and map calculation using Servalcat. *Acta Crystallogr. D Struct. Biol.* **77**, 1282–1291 (2021).

97. E. Krissinel, K. Henrick, Inference of macromolecular assemblies from crystalline state. *J. Mol. Biol.* **372**, 774–797 (2007).

98. M. Lindau, E. Neher, Patch-clamp techniques for time-resolved capacitance measurements in single cells. *Pflügers Arch.* **411**, 137–146 (1988).

99. Z. Jing, M. A. Rutherford, H. Takago, T. Frank, A. Fejtova, D. Khimich, T. Moser, N. Strenzke, Disruption of the presynaptic cytomatrix protein bassoon degrades ribbon anchorage, multiquantal release, and sound encoding at the hair cell afferent synapse. *J. Neurosci.* **33**, 4456–4467 (2013).

100. A. M. Taberner, M. C. Liberman, Response properties of single auditory nerve fibers in the mouse. *J. Neurophysiol.* **93**, 557–569 (2005).

101. A. B. Wong, M. A. Rutherford, M. Gabrielaitis, *T.* Pangršič, F. Göttfert, T. Frank, S. Michanski, S. Hell, F. Wolf, C. Wichmann, T. Moser, Developmental refinement of hair cell synapses tightens the coupling of Ca^{2+} influx to exocytosis. *EMBO J.* **33**, 247–264 (2014).

102. P. Jean, D. L. de la Morena, S. Michanski, L. M. J. Tobón, R. Chakrabarti, M. M. Picher, J. Neef, S. Jung, M. Gültas, S. Maxeiner, A. Neef, C. Wichmann, N. Strenzke, C. Grabner, T. Moser, The synaptic ribbon is critical for sound encoding at high rates and with temporal precision. *eLife* **7**, e29275 (2018).

103. D. N. Mastronarde, Automated electron microscope tomography using robust prediction of specimen movements. *J. Struct. Biol.* **152**, 36–51 (2005).

104. J. R. Kremer, D. N. Mastronarde, J. R. McIntosh, Computer visualization of three-dimensional image data using IMOD. *J. Struct. Biol.* **116**, 71–76 (1996).

105. S. Muth, F. Moschref, L. Freckmann, S. Mutschall, I. Hojas-Garcia-Plaza, J. N. Bahr, A. Petrovic, T. T. Do, V. Schwarze, A. Archit, K. Weyand, S. Michanski, L. Maus, C. Imig, N. Brose, C. Wichmann, R. Fernandez-Busnadiego, T. Moser, S. O. Rizzoli, B. H. Cooper, C. Pape, SynapseNet: Deep learning for automatic synapse reconstruction. *Mol. Biol. Cell*, mbcE24110519 (2025).

106. N. Sofroniew, T. Lambert, G. Bokota, J. Nunez-Iglesias, P. Sobolewski, A. Sweet, L. Gaifas, K. Evans, A. Burt, D. Doncila Pop, K. Yamauchi, M. Weber Mendonça, G. Buckley, W.-M. Vierdag, L. Royer, A. Can Solak, K. I. S. Harrington, J. Ahlers, D. Althviz Moré, O. Amsalem, A. Anderson, A. Annex, P. Boone, J. Bragantini, M. Bussonnier, C. Caporal, J. Eglinger, A. Eisenbarth, J. Freeman, C. Gohlke, K. Gunalan, H. Har-Gil, M. Harfouche, V. Hilsenstein, K. Hutchings, J. Lauer, G. Lichtner, Z. Liu, L. Liu, A. Lowe, L. Marconato, S. Martin, A. McGovern, L. Migas, N. Miller, H. Muñoz, J.-H. Müller, C. Nauroth-Kreß, D. Palecek, C. Pape, E. Perlman, K. Pevey, G. Peña-Castellanos, A. Pierré, D. Pinto, J. Rodríguez-Guerra, D. Ross, C. T. Russell, J. Ryan, G. Selzer, M. Smith, P. Smith, K. Sofiiuk, J. Soltwedel, D. Stansby, J. Vanaret, P. Wadhwa, M. Weigert, J. Windhager, P. Winston, R. Zhao, napari: A multi-dimensional image viewer for Python, version v0.5.5 (Zenodo, 2024); https://doi.org/10.5281/zenodo.14427406.

107. S. Krinner, T. Butola, S. Jung, C. Wichmann, T. Moser, RIM-binding protein 2 promotes a large number of CaV1.3 Ca^{2+}-channels and contributes to fast synaptic vesicle replenishment at hair cell active zones. *Front. Cell Neurosci.* **11**, 334 (2017).

108. A. Zhang, H. Yu, C. Liu, C. Song, The Ca^{2+} permeation mechanism of the ryanodine receptor revealed by a multi-site ion model. *Nat. Commun.* **11**, 922 (2020).

109. J. Huang, S. Rauscher, G. Nawrocki, T. Ran, M. Feig, B. L. de Groot, H. Grubmüller, A. D. MacKerell, CHARMM36m: An improved force field for folded and intrinsically disordered proteins. *Nat. Methods* **14**, 71–73 (2017).

110. M. J. Abraham, T. Murtola, R. Schulz, S. Páll, J. C. Smith, B. Hess, E. Lindahl, GROMACS: High performance molecular simulations through multi-level parallelism from laptops to supercomputers. *SoftwareX* **1–2**, 19–25 (2015).

111. G. Bussi, D. Donadio, M. Parrinello, Canonical sampling through velocity rescaling. *J. Chem. Phys.* **126**, 014101 (2007).

112. M. Bernetti, G. Bussi, Pressure control using stochastic cell rescaling. *J. Chem. Phys.* **153**, 114107 (2020).

113. B. Hess, P-LINCS: A parallel linear constraint solver for molecular simulation. *J. Chem. Theory Comput.* **4**, 116–122 (2008).

114. U. Essmann, L. Perera, M. L. Berkowitz, T. Darden, H. Lee, L. G. Pedersen, A smooth particle mesh Ewald method. *J. Chem. Phys.* **103**, 8577–8593 (1995).

115. E. Jurrus, D. Engel, K. Star, K. Monson, J. Brandi, L. E. Felberg, D. H. Brookes, L. Wilson, J. Chen, K. Liles, M. Chun, P. Li, D. W. Gohara, T. Dolinsky, R. Konecny, D. R. Koes, J. E. Nielsen, T. Head-Gordon, W. Geng, R. Krasny, G.-W. Wei, M. J. Holst, J. A. McCammon, N. A. Baker, Improvements to the APBS biomolecular solvation software suite. *Protein Sci.* **27**, 112–128 (2018).

116. F. B. Naughton, I. Alibay, J. Barnoud, E. Barreto-Ojeda, O. Beckstein, C. Bouysset, O. Cohen, R. J. Gowers, H. MacDermott-Opeskin, M. Matta, M. N. Melo, T. Reddy, L. Wang, Y. Zhuang, MDAnalysis 2.0 and beyond: Fast and interoperable, community driven simulation analysis. *Biophys. J.* **121**, 272a–273a (2022).

117. Y.-I. Iwasa, S.-Y. Nishio, A. Sugaya, Y. Kataoka, Y. Kanda, M. Taniguchi, K. Nagai, Y. Naito, T. Ikezono, R. Horie, Y. Sakurai, R. Matsuoka, H. Takeda, S. Abe, C. Kihara, T. Ishino, S.-Y. Morita, S. Iwasaki, M. Takahashi, T. Ito, Y. Arai, S.-I. Usami, OTOF mutation analysis with massively parallel DNA sequencing in 2,265 Japanese sensorineural hearing loss patients. *PLOS ONE* **14**, e0215932 (2019).

118. F. Mirghomizadeh, M. Pfister, F. Apaydin, C. Petit, S. Kupka, C. M. Pusch, H. P. Zenner, N. Blin, Substitutions in the conserved C2C domain of otoferlin cause DFNB9, a form of nonsyndromic autosomal recessive deafness. *Neurobiol. Dis.* **10**, 157–164 (2002).

119. Y. Wang, Y. Lu, J. Cheng, L. Zhang, D. Han, H. Yuan, Novel OTOF gene mutations identified using a massively parallel DNA sequencing technique in DFNB9 deafness. *Acta Otolaryngol.* **138**, 865–870 (2018).

120. J. Romanos, L. Kimura, M. L. Fávero, F. A. R. Izarra, M. T. B. de Mello Auricchio, A. C. Batissoco, K. Lezirovitz, R. S. Abreu-Silva, R. C. Mingroni-Netto, Novel OTOF mutations in Brazilian patients with auditory neuropathy. *J. Hum. Genet.* **54**, 382–385 (2009).

121. M. Yildirim-Baylan, G. Bademci, D. Duman, H. Ozturkmen-Akay, S. Tokgoz-Yilmaz, M. Tekin, Evidence for genotype-phenotype correlation for OTOF mutations. *Int. J. Pediatr. Otorhinolaryngol.* **78**, 950–953 (2014).

122. C. L. Ford, W. J. Riggs, T. Quigley, O. P. Keifer, J. P. Whitton, V. Valayannopoulos, The natural history, clinical outcomes, and genotype–phenotype relationship of otoferlin-related hearing loss: A systematic, quantitative literature review. *Hum. Genet.* **142**, 1429–1449 (2023).

123. Y.-I. Iwasa, S.-Y. Nishio, H. Yoshimura, A. Sugaya, Y. Kataoka, Y. Maeda, Y. Kanda, K. Nagai, Y. Naito, H. Yamazaki, T. Ikezono, H. Matsuda, M. Nakai, R. Tona, Y. Sakurai, R.

Motegi, H. Takeda, M. Kobayashi, C. Kihara, T. Ishino, S.-Y. Morita, S. Iwasaki, M. Takahashi, S. Furutate, S.-I. Oka, T. Kubota, Y. Arai, Y. Kobayashi, D. Kikuchi, T. Shintani, N. Ogasawara, Y. Honkura, S. Izumi, M. Hyogo, Y. Ninoyu, M. Suematsu, J. Nakayama, N. Tsuchihashi, M. Okami, H. Sakata, H. Yoshihashi, T. Kobayashi, K. Kumakawa, T. Yoshida, T. Esaki, S.-I. Usami, Detailed clinical features and genotype-phenotype correlation in an OTOF-related hearing loss cohort in Japan. *Hum. Genet.* **141**, 865–875 (2022).

124. Y.-M. Zhu, Q. Li, X. Gao, Y.-F. Li, Y.-L. Liu, P. Dai, X.-P. Li, Familial temperature-sensitive auditory neuropathy: Distinctive clinical courses caused by variants of the OTOF gene. *Front. Cell Dev. Biol.* **9**, 732930 (2021).

125. A. M. Fedick, C. Jalas, A. Swaroop, E. E. Smouha, B. D. Webb, Identification of a novel pathogenic OTOF variant causative of nonsyndromic hearing loss with high frequency in the Ashkenazi Jewish population. *Appl. Clin. Genet.* **9**, 141–146 (2016).
